# The global, regional, and national burden of colorectal cancer and its attributable risk factors in 195 countries and territories, 1990–2017: a systematic analysis for the Global Burden of Disease Study 2017

**DOI:** 10.1016/S2468-1253(19)30345-0

**Published:** 2019-10-21

**Authors:** GBD 2017 Colorectal Cancer Collaborators, Saeid Safiri, Sadaf G Sepanlou, Kevin S Ikuta, Catherine Bisignano, Hamideh Salimzadeh, Alireza Delavari, Reza Ansari, Gholamreza Roshandel, Shahin Merat, Christina Fitzmaurice, Lisa M Force, Molly R Nixon, Hedayat Abbastabar, Kedir Hussein Abegaz, Mohsen Afarideh, Ayat Ahmadi, Muktar Beshir Ahmed, Tomi Akinyemiju, Fares Alahdab, Raghib Ali, Mahtab Alikhani, Vahid Alipour, Syed Mohamed Aljunid, Majid Abdulrahman Hamad Almadi, Amir Almasi-Hashiani, Rajaa M Al-Raddadi, Nelson Alvis-Guzman, Saeed Amini, Nahla Hamed Anber, Alireza Ansari-Moghaddam, Jalal Arabloo, Zohreh Arefi, Mohammad Asghari Jafarabadi, Abbas Azadmehr, Alaa Badawi, Nafiseh Baheiraei, Till Winfried Bärnighausen, Huda Basaleem, Masoud Behzadifar, Meysam Behzadifar, Yaschilal Muche Belayneh, Kidanemaryam Berhe, Krittika Bhattacharyya, Belete Biadgo, Ali Bijani, Antonio Biondi, Tone Bjørge, Antonio M Borzì, Cristina Bosetti, Ibrahim R. Bou-Orm, Hermann Brenner, Andrey Nikolaevich Briko, Nikolay Ivanovich Briko, Giulia Carreras, Félix Carvalho, Carlos A Castañeda-Orjuela, Ester Cerin, Peggy Pei-Chia Chiang, Onyema Greg Chido-Amajuoyi, Ahmad Daryani, Dragos Virgil Davitoiu, Gebre Teklemariam Demoz, Rupak Desai, Mostafa Dianati nasab, Aziz Eftekhari, Iman El Sayed, Iffat Elbarazi, Mohammad Hassan Emamian, Aman Yesuf Endries, Firooz Esmaeilzadeh, Alireza Esteghamati, Arash Etemadi, Farshad Farzadfar, Eduarda Fernandes, João C Fernandes, Irina Filip, Florian Fischer, Masoud Foroutan, Mohamed M Gad, Silvano Gallus, Fatemeh Ghaseni-Kebria, Ahmad Ghashghaee, Giuseppe Gorini, Nima Hafezi-Nejad, Arvin Haj-Mirzaian, Arya Haj-Mirzaian, Susan Hasanpour-Heidari, Amir Hasanzadeh, Soheil Hassanipour, Simon I Hay, Chi Linh Hoang, Mihaela Hostiuc, Mowafa Househ, Olayinka Stephen Ilesanmi, Milena D. Ilic, Kaire Innos, Seyed Sina Naghibi Irvani, Farhad Islami, Anelisa Jaca, Nader Jafari Balalami, Nastaran Jafari delouei, Morteza Jafarinia, Mohammad Ali Jahani, Mihajlo Jakovljevic, Spencer L. James, Mehdi Javanbakht, Ensiyeh Jenabi, Ravi Prakash Jha, Farahnaz Joukar, Amir Kasaeian, Tesfaye Dessale Kassa, Mesfin Wudu Kassaw, Andre Pascal Kengne, Yousef Saleh Khader, Mojtaba Khaksarian, Rovshan Khalilov, Ejaz Ahmad Khan, Maryam Khayamzadeh, Maryam Khazaee-Pool, Salman Khazaei, Fatemeh Khosravi Shadmani, Jagdish Khubchandani, Daniel Kim, Adnan Kisa, Sezer Kisa, Jonathan M Kocarnik, Hamidreza Komaki, Jacek A Kopec, Ai Koyanagi, Ernst J. Kuipers, Vivek Kumar, Carlo La Vecchia, Faris Hasan Lami, Alan D Lopez, Platon D Lopukhov, Raimundas Lunevicius, Azeem Majeed, Maryam Majidinia, Amir Manafi, Navid Manafi, Ana-Laura Manda, Fariborz Mansour-Ghanaei, Lorenzo Giovanni Mantovani, Dhruv Mehta, Toni Meier, Hagazi Gebre Meles, Walter Mendoza, Tomislav Mestrovic, Bartosz Miazgowski, Tomasz Miazgowski, Seyed Mostafa Mir, Hamed Mirzaei, Karzan Abdulmuhsin Mohammad, Naser Mohammad Gholi Mezerji, Abdollah Mohammadian-Hafshejani, Milad Mohammadoo-Khorasani, Shafiu Mohammed, Farnam Mohebi, Ali H Mokdad, Lorenzo Monasta, Maryam Moossavi, Ghobad Moradi, Farhad Moradpour, Rahmatollah Moradzadeh, Azin Nahvijou, Gurudatta Naik, Farid Najafi, Javad Nazari, Ionut Negoi, Cuong Tat Nguyen, Trang Huyen Nguyen, Dina Nur Anggraini Ningrum, Felix Akpojene Ogbo, Andrew T Olagunju, Tinuke O Olagunju, Adrian Pana, David M. Pereira, Majid Pirestani, Akram Pourshams, Hossein Poustchi, Mostafa Qorbani, Mohammad Rabiee, Navid Rabiee, Amir Radfar, Marveh Rahmati, Fatemeh Rajati, David Laith Rawaf, Salman Rawaf, Robert C Reiner, Andre M N Renzaho, Nima Rezaei, Aziz Rezapour, Anas M Saad, Seyedmohammad Saadatagah, Basema Saddik, Farkhonde Salehi, Saleh Salehi Zahabi, Inbal Salz, Abdallah M Samy, Juan Sanabria, Milena M Santric Milicevic, Arash Sarveazad, Maheswar Satpathy, Ione J C Schneider, Mario Sekerija, Faramarz Shaahmadi, Hosein Shabaninejad, Morteza Shamsizadeh, Zeinab Sharafi, Mehdi Sharif, Amrollah Sharifi, Sara Sheikhbahaei, Reza Shirkoohi, Sudeep K Siddappa Malleshappa, Diego Augusto Santos Silva, Mekonnen Sisay, Catalin-Gabriel Smarandache, Moslem Soofi, Kjetil Soreide, Sergey Soshnikov, Vladimir I. Starodubov, Michelle L. Subart, Mark JM Sullman, Rafael Tabarés-Seisdedos, Amir Taherkhani, Berhe etsay Tesfay, Roman Topor-Madry, Eugenio Traini, Bach Xuan Tran, Khanh Bao Tran, Irfan Ullah, Olalekan A Uthman, Marco Vacante, Amir Vahedian-Azimi, Alessandro Valli, Elena Varavikova, Isidora S Vujcic, Ronny Westerman, Vahid Yazdi-Feyzabadi, Engida Yisma, Chuanhua Yu, Vesna Zadnik, Telma Zahirian Moghadam, Leila Zaki, Hamed Zandian, Zhi-Jiang Zhang, Christopher J L Murray, Mohsen Naghavi, Reza Malekzadeh

## Abstract

**Background:**

Data about the global, regional, and country-specific variations in the levels and trends of colorectal cancer are required to understand the impact of this disease and the trends in its burden to help policy makers allocate resources. Here we provide a status report on the incidence, mortality, and disability caused by colorectal cancer in 195 countries and territories between 1990 and 2017.

**Methods:**

Vital registration, sample vital registration, verbal autopsy, and cancer registry data were used to generate incidence, death, and disability-adjusted life-year (DALY) estimates of colorectal cancer at the global, regional, and national levels. We also determined the association between development levels and colorectal cancer age-standardised DALY rates, and calculated DALYs attributable to risk factors that had evidence of causation with colorectal cancer. All of the estimates are reported as counts and age-standardised rates per 100 000 person-years, with some estimates also presented by sex and 5-year age groups.

**Findings:**

In 2017, there were 1·8 million (95% UI 1·8–1·9) incident cases of colorectal cancer globally, with an age-standardised incidence rate of 23·2 (22·7–23·7) per 100 000 person-years that increased by 9·5% (4·5–13·5) between 1990 and 2017. Globally, colorectal cancer accounted for 896 000 (876 300–915 700) deaths in 2017, with an age-standardised death rate of 11·5 (11·3–11·8) per 100 000 person-years, which decreased between 1990 and 2017 (−13·5% [–18·4 to −10·0]). Colorectal cancer was also responsible for 19·0 million (18·5–19·5) DALYs globally in 2017, with an age-standardised rate of 235·7 (229·7–242·0) DALYs per 100 000 person-years, which decreased between 1990 and 2017 (−14·5% [–20·4 to −10·3]). Slovakia, the Netherlands, and New Zealand had the highest age-standardised incidence rates in 2017. Greenland, Hungary, and Slovakia had the highest age-standardised death rates in 2017. Numbers of incident cases and deaths were higher among males than females up to the ages of 80–84 years, with the highest rates observed in the oldest age group (≥95 years) for both sexes in 2017. There was a non-linear association between the Socio-demographic Index and the Healthcare Access and Quality Index and age-standardised DALY rates. In 2017, the three largest contributors to DALYs at the global level, for both sexes, were diet low in calcium (20·5% [12·9–28·9]), alcohol use (15·2% [12·1–18·3]), and diet low in milk (14·3% [5·1–24·8]).

**Interpretation:**

There is substantial global variation in the burden of colorectal cancer. Although the overall colorectal cancer age-standardised death rate has been decreasing at the global level, the increasing age-standardised incidence rate in most countries poses a major public health challenge across the world. The results of this study could be useful for policy makers to carry out cost-effective interventions and to reduce exposure to modifiable risk factors, particularly in countries with high incidence or increasing burden.

**Funding:**

Bill & Melinda Gates Foundation.

## Introduction

In 2016, cancer accounted for more than 213 million disability-adjusted life-years (DALYs) and 8·9 million deaths globally.^1^, ^2^ The burden of cancer is usually reported in aggregated form,^1^, ^3^ but cancer-specific reports allow a more detailed exploration of the problem by providing information that is useful for the development and evaluation of cancer-specific prevention programmes, screening strategies, treatment, and resource allocation. An understanding of the geographical and temporal trends in colorectal cancer is important because it was the second leading cause of death (age-standardised and all ages) among cancers globally in 2017 and the 16th leading cause of death among all diseases and injuries.^4^ Trends in the burden of colorectal cancer have been subject to substantial changes across the world because of the expansion of screening programmes, with wide recommendation of colonoscopy in the late 1990s, as well as changes in risk factors associated with colorectal cancer.^5^, ^6^

**Research in context**

**Evidence before this study**

This study is part of the Global Burden of Diseases, Injuries, and Risk Factors Study (GBD), which is the most comprehensive effort to date to measure epidemiological levels and trends. In its most up-to-date iteration, 359 diseases and injuries; 282 causes of death; and 84 behavioural, environmental and occupational, and metabolic risk factors were studied. The International Agency for Research on Cancer generates periodically updated estimates for all cancers including colorectal cancer in the Global Cancer Incidence, Mortality and Prevalence (GLOBOCAN) project. The burden of colorectal cancer has been investigated in previous research using GLOBOCAN data, but these studies have several limitations. The global burden of colorectal cancer is reported in terms of incidence and mortality, but important measures such as years of life lost, years lived with disability, and disability-adjusted life-years are not reported. The measures GLOBOCAN produces do not allow for comparability of the burden of disability or premature mortality between countries or with other causes. The temporal trends in GLOBOCAN estimates begin in 2002 and have occurred globally at 4-year or 6-year intervals with 95% uncertainty intervals provided only for the 2018 estimates. Using a consistent methodology to produce annual estimates dating back to 1990 provides a rich context for the burden estimates. Finally, the burden of colorectal cancer attributable to risk factors has not previously been calculated.

**Added value of this study**

To our knowledge, this study is the first to report the incidence, mortality, and disability from colorectal cancer and its attributable risk factors from 1990 to 2017 in 195 countries and territories, by age, sex, Socio-demographic Index (a composite of sociodemographic factors), and Healthcare Access and Quality Index, an indicator of health system performance.

**Implications of all the available evidence**

Colorectal cancer remains a substantial public health challenge across the globe. Age-standardised incidence rates increased in most countries from 1990 to 2017, and the age-standardised death rate decreased at the global level and decreased particularly in countries high on the Socio-demographic Index. The burden of colorectal cancer was mainly attributed to dietary risks, alcohol use, and smoking. Further research is required to better understand the increases in incidence of colorectal cancer and to improve prevention, early detection, and treatment of this disease.

Whereas colorectal cancer age-standardised death rates have stabilised or declined in many high-income countries, which historically had the highest burden of colorectal cancer in the world,^7^ the burden is increasing in most low-income and middle-income countries,^8^ possibly as a result of ageing populations, urbanisation, and increased prevalence of westernised lifestyle risk factors, such as alcohol consumption, obesity, smoking, and suboptimal diet.^9^, ^10^ The global burden of colorectal cancer attributable to various modifiable risk factors has not been described elsewhere and is an important estimate to report because it has implications for policy making and prevention efforts.

Studies reporting the global burden of colorectal cancer have been published previously but have several limitations. Specifically, previous estimates reported the global burden of colorectal cancer in terms of incidence and mortality but did not aim to calculate important measures such as years of life lost (YLLs), years lived with disability (YLDs), and DALYs.^3^, ^7^, ^11^, ^12^, ^13^, ^14^ Moreover, although the burden of colorectal cancer and trends associated with this disease have been reported up to 2018, the temporal trends occur at 4-year or 6-year intervals for most countries and 95% uncertainty intervals (UIs) have been provided only for the most recent global estimates in 2018.^7^, ^11^, ^12^, ^13^, ^15^ Finally, the association between countries' development status and colorectal cancer burden has previously been described using Global Cancer Incidence, Mortality and Prevalence (GLOBOCAN) data from only a subset of countries.^16^ We aimed to report the incidence, mortality, and disability due to colorectal cancer and its attributable risk factors from 1990 to 2017 in 195 countries and territories, by age, sex, Socio-demographic Index (SDI; a composite of socio-demographic factors), and Healthcare Access and Quality (HAQ) Index, an indicator of health system performance.

## Methods

### Overview

This study is part of the Global Burden of Diseases, Injuries, and Risk Factors Study (GBD), which covers seven super-regions, consisting of 21 regions containing 195 countries and territories. The most up-to-date iteration, GBD 2017, reported estimates for 359 diseases and injuries; 282 causes of death; and 84 behavioural, environmental and occupational, and metabolic risk factors. The general methodology used and updates to the methodology have been previously presented in GBD 2017 papers.^4^, ^17^, ^18^, ^19^, ^20^, ^21^ Briefly, the mortality-to-incidence ratio (MIR) estimation was updated from GBD 2016, with use of the HAQ Index rather than the SDI in the data cleaning and modelling process, and the spatiotemporal Gaussian process regression approach was also updated. Covariate inputs for the Cause of Death Ensemble model (CODEm) were updated and changed on the basis of recommendations from GBD collaborators. The rates were standardised according to the GBD world population and reported per 100 000 person-years.^17^ The method for propagating uncertainty in this paper is similar to that used in previous GBD 2017 papers.^4^, ^19^ The distribution of every step in the computation process is stored in 1000 draws that are used for every other step in the process. The distributions are characterised from the sampling error of data inputs, the uncertainty of the model coefficients, MIRs, and age-specific death rates. GBD assumes that uncertainty in the MIR is independent of uncertainty in the estimated age-specific death rates. Final estimates were computed using the mean estimate across 1000 draws, and the 95% UIs were specified on the basis of the 25th and 975th ranked values across all 1000 draws. The GBD study is compliant with the Guidelines for Accurate and Transparent Health Estimates Reporting (GATHER).

### Data sources

All cancers coded as C18–C21, D01.0–D01.2, and D12–D12.8 in the 10th revision of the International Classification of Diseases were considered to be colorectal cancer.^19^ Vital registration (18 857 site-years of data), sample vital registration (761 site-years), verbal autopsy (660 site-years), and cancer registry (4474 site-years) data from GBD 2017 were used in this study.^4^ Vital registration is the system by which governments record the vital events of their residents, including causes of death. In sample vital registration, vital events are recorded in nationally representative cluster samples to estimate birth rates, deaths rates, and causes of death for the total population in countries where high coverage of vital registration is not available. Verbal autopsy is a method by which trained interviewers collect information about the signs, symptoms, and demographic characteristics of a recently deceased person from an individual familiar with the deceased to determine individuals' causes of death and cause-specific mortality fractions in populations without a complete vital registration system. Finally, a cancer registry gathers data on every person with cancer in a defined population, usually comprising residents in a well defined geographical region. The details on data quality rating for 195 countries and territories are provided in the appendix (pp 11–17). More detailed information about the data sources used for each country can be found on the GBD 2017 Data Input Sources Tool website.

### Mortality estimates

Mortality data from vital registration, sample vital registration, and verbal autopsy were sparse. Therefore, incidence data from cancer registries were converted into mortality data by modelling the MIRs independently. We modelled MIRs using the locations that had both incidence and mortality data available for the same year. The initial MIR model used a linear-step mixed-effects model with logit link functions, as well as the HAQ Index, age, and sex as covariates. The resulting estimates were then smoothed over space and time, and adjusted with spatiotemporal Gaussian process regression.^18^ We used the observed mortality (from vital registration and verbal autopsy) and mortality estimates (computed from the MIRs and incidence data) as inputs for a CODEm.^4^ Country-level covariates used for the CODEm and the assumed directions are described in the appendix (p 18). We used CODEm to select which predictors produce the best fit to the data. We used the CoDCorrect algorithm to adjust the sum of predicted single-cause mortalities in an age–sex–location–year group to be consistent with the results from all-cause mortality estimation.^4^

### Non-fatal estimates

The final mortality estimates were divided by the MIR to compute colorectal cancer incidence.^19^ Colorectal cancer prevalence was calculated by estimating 10-year survival based on MIRs and adjusting for expected background mortality. The cohort members who had survived more than 10 years were assumed to be cured, and one of the two sequelae were assigned to them: the diagnosis and primary therapy phase or the controlled phase. The controlled phase included all patients who survived more than 10 years and who had finished primary therapy. The prevalence for the cohort in which people died during the 10-year period was categorised into four sequelae (appendix p 20). The diagnosis and primary therapy phase was defined as 4·0 months, the metastatic phase as 9·7 months, and terminal phase as 1 month.^22^, ^23^ The remaining time was assigned to the controlled phase. The duration of sequela one (diagnosis and primary therapy) described by Allgar and colleagues^22^ was used and 2 months were added to account for the average treatment duration. Duration of sequela two (controlled phase) was 10 years for the survivors minus the duration of the other sequelae. Duration of sequela three (metastatic phase) was based on Surveillance, Epidemiology, and End Results (SEER) data for median survival of patients with stage IV disease. A duration of 1 month for sequela four (terminal phase) was used for all cancers.^22^

To estimate procedure-related disability for all locations by age, sex, and year, we used hospital data on the proportion of patients that undergo ostomies (ie, the procedure proportion) as our input for a DisMod-MR 2.1 proportion model.^19^ We determined through a literature review that an average of 58% of all ostomies are for colorectal cancer, so we multiplied the all-cause ostomies by 0·58.^24^, ^25^, ^26^ We applied these procedure proportions to the number of incident cases of colorectal cancer and multiplied that by the proportion of the incident population that had survived for 10 years. This process gave us the number of incident cases of colorectal cancer that involved an ostomy procedure and survived beyond 10 years. We then input these cases into DisMod-MR 2.1. This model produced estimates of incidence and lifetime prevalent cases of people with colorectal cancer-related stomas who have survived beyond 10 years.^19^

Following this process, to estimate the sequela-specific YLDs, procedure sequelae prevalence and general sequela prevalence rates were multiplied by the sequela-specific disability weight. The disability weights for four sequelae and one procedure can be found in the appendix (p 19).^19^ The disability weights ranged from 0 (perfect health) to 1 (equivalent to death). GBD uses different disability weights for the four phases of colorectal cancer, but these weights are the same for all cancers.

YLLs were calculated by multiplying the estimated number of deaths by age with a standard life expectancy at that age. Finally, DALYs were calculated by summing YLDs and YLLs.

### SDI and HAQ Index

We used the GBD 2017 SDI and GBD 2016 HAQ Index to determine the association a country's development level had with colorectal cancer age-standardised DALY rates. Examining the association of development level (SDI) and health system performance (HAQ Index) with colorectal cancer burden is important because these factors affect the prevalence of cancer risk factors. In GBD 2017, the SDI was revised to better reflect the development status of each country.^4^, ^18^, ^19^, ^20^, ^21^ The SDI ranges from 0 (worst) to 1 (best) and incorporates the total fertility rate in women under the age of 25 years, mean education for individuals aged 15 years and older, and lag-distributed income per person. The HAQ Index reflects the personal health-care access and quality for 195 countries and territories and was calculated on the basis of amenable mortality (ie, deaths from causes that should not occur in the presence of effective medical care). The HAQ Index ranges from 0 (worst) to 100 (best). Further details on the HAQ Index are presented elsewhere.^27^

### Risk factors

We selected risk factors that had evidence of causation with colorectal cancer. We extracted the relative risks and exposure estimates from all available data sources. We calculated a population attributable fraction as the proportional reduction in a health outcome that would occur if exposure to a risk factor was reduced to the theoretical minimum exposure level. We reported the proportion of DALYs due to colorectal cancer that were attributable to smoking, high body-mass index, high fasting plasma glucose, low physical activity, and five dietary risks (diets low in calcium, milk, and fibre, and diets high in red meat and processed meat). Details on definitions of these risk factors and their relative risk for colorectal cancer, prevalence of risk factors, and methods for quantifying the proportion of the burden of colorectal cancer attributable to these risk factors are described elsewhere.^18^ The DALYs due to colorectal cancer that were attributable to each risk factor were estimated by multiplying the total DALYs for colorectal cancer by the population attributable fraction for the risk–outcome pair for each age group, sex, location, and year.

### Role of the funding source

The funder of the study had no role in study design; the collection, analysis, or interpretation of the data; or the writing of the report. The corresponding authors had full access to the data and had responsibility for final submission of the manuscript.

## Results

In 2017, there were 1·8 million (95% UI 1·8–1·9) incident cases of colorectal cancer, with an age-standardised incidence rate of 23·2 (22·7–23·7) per 100 000 person-years. The age-standardised incidence rate showed an increase of 9·5% (4·5–13·5) from 1990 to 2017 (table). Colorectal cancer also accounted for 896 000 (876 300–915 700) deaths globally, with an age-standardised death rate of 11·5 (11·3–11·8) per 100 000 person-years and a decrease in age-standardised death rates from 1990 to 2017 (−13·5% [–18·4 to −10·0]; appendix pp 21–29). Colorectal cancer was responsible for 19·0 million (18·5–19·5) DALYs globally, with an age-standardised rate of 235·7 (229·7–242·0) DALYs per 100 000 person-years. The age-standardised DALY rate decreased from 1990 to 2017 (−14·5% [–20·4 to −10·3]; appendix pp 30–39).

**Table:** Incident cases of colorectal cancer for both sexes and percentage change in age-standardised rates by location, 1990–2017

			**1990**		**2017**		**Percentage change in age-standardised incidence rates, 1990–2017**
			Incident cases	Age-standardised incidence rate (per 100 000 person-years)	Incident cases	Age-standardised incidence rate (per 100 000 person-years)	
**Global**			**826 357 (807 380 to 854 834)**	**21·2 (20·7 to 21·9)**	**1 833 451 (1 791 865 to 1 873 464)**	**23·2 (22·7 to 23·7)**	**9·5% (4·5 to 13·5)**
**Central Europe, eastern Europe, and central Asia**							
	Central Asia		5534 (5430 to 5645)	11·2 (11·0 to 11·4)	8977 (8558 to 9410)	12·3 (11·8 to 12·9)	10·0% (5·3 to 14·8)
		Armenia	418 (397 to 441)	14·7 (14·0 to 15·5)	772 (728 to 815)	18·7 (17·7 to 19·8)	27·1% (18·1 to 36·5)
		Azerbaijan	536 (506 to 568)	9·9 (9·3 to 10·4)	1210 (1028 to 1383)	12·9 (11·0 to 14·6)	30·1% (11·6 to 49·0)
		Georgia	737 (698 to 779)	11·7 (11·1 to 12·4)	901 (836 to 964)	15·7 (14·6 to 16·8)	34·2% (23·4 to 46·0)
		Kazakhstan	2064 (1992 to 2146)	15·5 (15·0 to 16·1)	2773 (2566 to 3009)	16·4 (15·2 to 17·8)	6·2% (−1·9 to 13·6)
		Kyrgyzstan	387 (364 to 411)	12·4 (11·7 to 13·2)	372 (344 to 421)	8·5 (7·9 to 9·5)	−31·5% (−37·3 to −22·6)
		Mongolia	91 (83 to 102)	8·5 (7·7 to 9·4)	183 (158 to 206)	8·2 (7·1 to 9·3)	−2·6% (−19·4 to 14·1)
		Tajikistan	229 (216 to 242)	7·5 (7·1 to 7·9)	430 (385 to 482)	8·0 (7·2 to 8·9)	6·8% (−4·8 to 18·8)
		Turkmenistan	155 (148 to 163)	7·4 (7·1 to 7·8)	353 (325 to 384)	9·6 (8·8 to 10·4)	28·8% (16·4 to 42·3)
		Uzbekistan	917 (882 to 953)	7·4 (7·1 to 7·7)	1982 (1757 to 2219)	9·5 (8·4 to 10·6)	28·2% (14·1 to 42·8)
	Central Europe		41 719 (41 148 to 42 319)	27·7 (27·3 to 28·1)	72 984 (70 812 to 75 162)	34·6 (33·5 to 35·6)	24·8% (20·8 to 29·0)
		Albania	184 (171 to 218)	8·1 (7·5 to 10·0)	454 (371 to 552)	11·2 (9·2 to 13·5)	37·5% (10·6 to 69·3)
		Bosnia and Herzegovina	671 (627 to 778)	16·2 (15·2 to 18·9)	1735 (1584 to 1896)	29·4 (27·0 to 32·0)	81·8% (61·7 to 100·4)
		Bulgaria	3092 (2983 to 3202)	24·1 (23·3 to 24·9)	5156 (4765 to 5545)	35·5 (32·8 to 38·1)	47·5% (35·6 to 59·5)
		Croatia	2211 (2126 to 2297)	34·4 (33·1 to 35·7)	3993 (3720 to 4278)	45·9 (42·9 to 49·2)	33·6% (23·0 to 44·4)
		Czech Republic	6800 (6579 to 7013)	48·6 (47·1 to 50·1)	8320 (7750 to 8966)	40·1 (37·4 to 43·2)	−17·5% (−23·9 to −10·3)
		Hungary	6117 (5932 to 6300)	40·8 (39·6 to 41·9)	8454 (7883 to 9040)	44·7 (41·7 to 47·7)	9·8% (2·1 to 18·0)
		Macedonia	304 (284 to 335)	15·9 (14·8 to 17·8)	812 (722 to 914)	24·1 (21·4 to 27·1)	51·6% (26·6 to 74·6)
		Montenegro	124 (113 to 136)	19·7 (17·9 to 21·5)	238 (215 to 265)	23·8 (21·6 to 26·6)	21·1% (6·2 to 38·2)
		Poland	10 892 (10 582 to 11 200)	24·1 (23·4 to 24·8)	20 482 (19 092 to 22 015)	29·7 (27·7 to 31·8)	23·3% (14·4 to 32·6)
		Romania	4736 (4576 to 4913)	16·5 (16·0 to 17·1)	10 989 (10 254 to 11 753)	30·5 (28·5 to 32·6)	84·4% (70·8 to 98·9)
		Serbia	3475 (3188 to 3840)	30·1 (27·7 to 33·1)	5971 (5507 to 6494)	38·4 (35·4 to 41·7)	27·5% (14·4 to 41·3)
		Slovakia	2275 (2178 to 2372)	37·5 (36·0 to 39·1)	4739 (4289 to 5177)	52·4 (47·5 to 57·1)	39·8% (24·3 to 54·4)
		Slovenia	837 (801 to 877)	33·6 (32·2 to 35·1)	1639 (1508 to 1785)	39·4 (36·2 to 43·0)	17·4% (7·3 to 29·1)
	Eastern Europe		68 421 (66 610 to 71 088)	23·8 (23·2 to 24·7)	103 116 (100 177 to 106 623)	30·2 (29·3 to 31·2)	26·8% (23·4 to 30·6)
		Belarus	2904 (2798 to 2999)	21·9 (21·1 to 22·6)	4478 (4078 to 5121)	28·3 (25·7 to 32·5)	29·1% (17·1 to 47·1)
		Estonia	588 (563 to 613)	27·9 (26·8 to 29·1)	929 (801 to 1065)	34·8 (30·1 to 40·2)	24·8% (6·8 to 44·4)
		Latvia	892 (862 to 925)	24·2 (23·3 to 25·0)	1205 (1066 to 1360)	29·8 (26·2 to 33·7)	23·2% (7·9 to 40·5)
		Lithuania	1061 (1026 to 1098)	22·9 (22·1 to 23·6)	1683 (1558 to 1806)	29·2 (27·1 to 31·4)	27·9% (17·5 to 38·6)
		Moldova	981 (941 to 1018)	21·1 (20·2 to 21·9)	1437 (1349 to 1539)	25·2 (23·6 to 26·9)	19·3% (10·8 to 28·5)
		Russia	42 907 (41 400 to 45 268)	23·1 (22·3 to 24·4)	69 283 (67 424 to 71 061)	29·9 (29·2 to 30·7)	29·5% (23·5 to 34·9)
		Ukraine	19 089 (18 412 to 19 805)	26·0 (25·1 to 26·9)	24 101 (22 571 to 25 877)	31·5 (29·6 to 33·8)	21·5% (13·4 to 30·2)
**High income**							
	Australasia		11 968 (11 694 to 12 218)	50·2 (49·1 to 51·2)	22 266 (20 408 to 24 232)	46·4 (42·5 to 50·6)	−7·4% (−15·7 to 1·0)
		Australia	9497 (9253 to 9741)	47·8 (46·6 to 48·9)	18 429 (16 592 to 20 418)	45·7 (41·2 to 50·8)	−4·3% (−14·6 to 6·3)
		New Zealand	2472 (2365 to 2589)	62·1 (59·5 to 64·9)	3837 (3562 to 4144)	50·2 (46·6 to 54·2)	−19·1% (−25·9 to −12·1)
	High-income Asia Pacific		67 498 (66 180 to 68 809)	33·2 (32·6 to 33·9)	183 789 (175 950 to 193 063)	41·9 (40·2 to 44·1)	26·1% (20·8 to 32·1)
		Brunei	32 (28 to 38)	31·2 (26·7 to 36·4)	139 (127 to 154)	43·8 (39·8 to 48·6)	40·5% (16·6 to 66·7)
		Japan	62 351 (61 081 to 63 664)	36·4 (35·6 to 37·1)	153 905 (146 718 to 161 765)	45·0 (43·1 to 47·3)	23·8% (18·3 to 30·0)
		Singapore	778 (751 to 808)	34·1 (32·9 to 35·3)	2394 (2213 to 2622)	34·9 (32·2 to 38·1)	2·4% (−6·3 to 12·4)
		South Korea	4337 (4184 to 4495)	14·3 (13·8 to 14·8)	27 351 (24 820 to 30 076)	32·5 (29·5 to 35·7)	127·3% (105·1 to 150·7)
	High-income North America		165 322 (163 317 to 167 704)	45·6 (45·0 to 46·2)	234 927 (228 060 to 241 844)	39·1 (37·9 to 40·3)	−14·2% (−17·2 to −11·4)
		Canada	13 301 (12 833 to 13 790)	40·1 (38·7 to 41·5)	25 661 (23 835 to 27 580)	38·5 (35·8 to 41·4)	−3·8% (−11·7 to 4·3)
		Greenland	15 (13 to 16)	45·4 (40·4 to 50·3)	25 (23 to 28)	39·0 (35·5 to 42·4)	−14·2% (−26·5 to −1·2)
		USA	152 002 (150 137 to 154 241)	46·1 (45·6 to 46·8)	209 237 (203 167 to 215 912)	39·1 (38·0 to 40·4)	−15·1% (−18·2 to −12·0)
	Southern Latin America		9098 (8881 to 9339)	19·5 (19·1 to 20·1)	20 898 (19 394 to 22 657)	25·5 (23·6 to 27·6)	30·4% (20·2 to 41·5)
		Argentina	6650 (6439 to 6875)	20·4 (19·8 to 21·0)	13 927 (12 487 to 15 469)	26·1 (23·4 to 29·0)	28·1% (14·8 to 43·3)
		Chile	1341 (1285 to 1396)	13·4 (12·9 to 14·0)	5154 (4626 to 5746)	22·2 (19·9 to 24·8)	65·5% (46·7 to 86·6)
		Uruguay	1106 (1067 to 1145)	27·8 (26·8 to 28·7)	1817 (1620 to 2025)	33·3 (29·6 to 37·3)	20·0% (6·1 to 34·5)
	Western Europe		220 737 (217 920 to 223 500)	37·3 (36·8 to 37·7)	347 288 (332 898 to 361 454)	38·7 (37·1 to 40·3)	3·8% (−0·6 to 8·1)
		Andorra	21 (17 to 26)	36·1 (29·6 to 44·0)	52 (42 to 63)	38·3 (30·8 to 46·1)	6·1% (−14·8 to 28·9)
		Austria	4883 (4718 to 5065)	40·8 (39·4 to 42·2)	5592 (5201 to 6011)	31·5 (29·3 to 33·9)	−22·6% (−28·6 to −16·3)
		Belgium	6258 (6013 to 6521)	39·7 (38·2 to 41·2)	8141 (7518 to 8809)	35·5 (32·7 to 38·4)	−10·5% (−18·0 to −2·3)
		Cyprus	170 (150 to 197)	19·8 (17·5 to 23·0)	551 (492 to 620)	29·0 (26·0 to 32·6)	46·1% (20·5 to 75·4)
		Denmark	2330 (2261 to 2399)	28·4 (27·6 to 29·2)	5175 (4762 to 5593)	45·6 (42·0 to 49·3)	60·5% (47·4 to 74·7)
		Finland	1784 (1731 to 1838)	24·7 (23·9 to 25·4)	3437 (3197 to 3725)	28·9 (26·9 to 31·3)	17·3% (7·5 to 27·9)
		France	29 412 (28 397 to 30 488)	34·3 (33·1 to 35·5)	45 501 (41 853 to 49 486)	33·0 (30·4 to 36·0)	−3·7% (−11·7 to 5·4)
		Germany	59 179 (57 557 to 60 958)	45·4 (44·2 to 46·7)	76 179 (68 038 to 84 803)	41·1 (36·7 to 45·8)	−9·4% (−19·0 to 0·9)
		Greece	2661 (2540 to 2784)	17·3 (16·5 to 18·0)	6556 (6083 to 7025)	27·6 (25·6 to 29·6)	60·1% (46·8 to 73·3)
		Iceland	87 (82 to 92)	29·7 (28·0 to 31·5)	169 (157 to 182)	31·7 (29·3 to 34·0)	6·5% (−3·4 to 16·7)
		Ireland	1643 (1582 to 1705)	39·7 (38·2 to 41·1)	2948 (2661 to 3280)	40·6 (36·7 to 45·2)	2·5% (−8·2 to 14·0)
		Israel	1307 (1251 to 1380)	26·7 (25·6 to 28·1)	3165 (2921 to 3438)	27·9 (25·8 to 30·4)	4·5% (−4·1 to 13·6)
		Italy	30 748 (29 557 to 31 888)	34·2 (32·9 to 35·4)	52 228 (48 427 to 56 835)	37·2 (34·3 to 40·4)	8·8% (−0·5 to 18·7)
		Luxembourg	233 (221 to 247)	41·8 (39·7 to 44·2)	409 (359 to 475)	42·1 (37·0 to 49·3)	0·9% (−11·7 to 17·5)
		Malta	109 (104 to 116)	25·4 (24·1 to 27·0)	306 (281 to 333)	34·4 (31·7 to 37·2)	35·2% (22·9 to 48·4)
		Netherlands	8553 (8241 to 8849)	41·9 (40·4 to 43·4)	16 948 (15 727 to 18 222)	50·9 (47·1 to 54·7)	21·3% (11·9 to 31·7)
		Norway	2861 (2811 to 2917)	41·7 (40·9 to 42·5)	4556 (4316 to 4796)	48·4 (46·0 to 51·0)	16·2% (9·8 to 22·7)
		Portugal	4052 (3901 to 4207)	29·5 (28·5 to 30·6)	9390 (8696 to 10288)	41·4 (38·4 to 45·3)	40·3% (28·5 to 54·8)
		Spain	17 169 (16 664 to 17 708)	30·8 (29·9 to 31·7)	41 133 (38 218 to 44 436)	43·4 (40·2 to 47·0)	40·8% (29·2 to 53·7)
		Sweden	5106 (4972 to 5255)	33·0 (32·1 to 33·9)	7130 (6693 to 7575)	34·7 (32·7 to 36·8)	5·1% (−1·6 to 12·2)
		Switzerland	2227 (2137 to 2321)	20·9 (20·1 to 21·8)	5032 (4597 to 5547)	29·4 (26·9 to 32·5)	40·3% (26·6 to 56·1)
		UK	39 729 (39 124 to 40 372)	42·7 (42·1 to 43·4)	52 331 (51 067 to 53 737)	41·7 (40·7 to 42·9)	−2·3% (−5·2 to 1·0)
**Latin America and Caribbean**							
	Andean Latin America		1770 (1608 to 1997)	8·7 (7·9 to 9·7)	7635 (6901 to 8372)	14·2 (12·9 to 15·6)	64·3% (41·6 to 89·3)
		Bolivia	324 (201 to 527)	10·3 (6·5 to 16·5)	1092 (799 to 1460)	13·2 (9·7 to 17·6)	28·0% (−9·2 to 77·0)
		Ecuador	415 (400 to 432)	7·7 (7·4 to 8·0)	1954 (1769 to 2160)	13·4 (12·2 to 14·8)	73·8% (56·4 to 93·4)
		Peru	1031 (947 to 1121)	8·6 (7·9 to 9·4)	4589 (3917 to 5349)	15·0 (12·8 to 17·5)	73·6% (45·2 to 107·2)
	Caribbean		4453 (4299 to 4655)	17·1 (16·5 to 17·8)	11 943 (11 109 to 12 868)	23·5 (21·8 to 25·3)	37·6% (29·2 to 47·0)
		Antigua and Barbuda	7 (7 to 8)	13·9 (13·0 to 15·0)	20 (18 to 21)	19·9 (18·2 to 21·7)	42·9% (27·1 to 59·8)
		The Bahamas	33 (30 to 35)	20·5 (19·2 to 22·0)	95 (85 to 105)	25·8 (23·2 to 28·6)	25·7% (9·6 to 44·1)
		Barbados	66 (62 to 71)	22·0 (20·7 to 23·3)	153 (138 to 168)	31·8 (28·6 to 34·8)	44·6% (28·4 to 61·7)
		Belize	7 (7 to 8)	7·7 (7·0 to 8·5)	30 (27 to 32)	11·4 (10·5 to 12·5)	47·6% (29·9 to 65·3)
		Bermuda	20 (19 to 22)	32·3 (30·0 to 34·4)	46 (41 to 50)	36·0 (32·4 to 39·6)	11·4% (−1·7 to 28·7)
		Cuba	2285 (2210 to 2369)	22·0 (21·2 to 22·7)	5629 (4988 to 6293)	29·9 (26·5 to 33·4)	36·0% (21·3 to 52·9)
		Dominica	9 (8 to 10)	12·0 (11·2 to 12·9)	16 (15 to 18)	17·4 (15·9 to 19·1)	44·8% (27·8 to 61·8)
		Dominican Republic	283 (260 to 307)	7·4 (6·8 to 8·0)	1277 (1100 to 1467)	13·9 (11·9 to 16·0)	87·7% (57·2 to 120·5)
		Grenada	11 (10 to 12)	15·6 (14·6 to 16·6)	29 (27 to 32)	18·7 (17·1 to 20·3)	19·6% (7·5 to 32·1)
		Guyana	41 (39 to 44)	10·6 (10·0 to 11·2)	79 (70 to 89)	13·2 (11·7 to 14·8)	23·9% (8·2 to 41·6)
		Haiti	333 (236 to 517)	10·7 (7·8 to 16·1)	803 (577 to 1164)	12·8 (9·4 to 18·1)	19·2% (−4·1 to 51·4)
		Jamaica	243 (229 to 262)	13·3 (12·5 to 14·2)	639 (538 to 743)	22·0 (18·5 to 25·6)	66·1% (36·5 to 96·9)
		Puerto Rico	726 (694 to 757)	19·5 (18·7 to 20·3)	2084 (1921 to 2254)	30·4 (28·1 to 32·9)	55·5% (43·2 to 69·2)
		Saint Lucia	11 (11 to 12)	12·7 (12·0 to 13·4)	31 (29 to 34)	15·1 (13·9 to 16·4)	18·9% (7·5 to 31·4)
		Saint Vincent and the Grenadines	10 (9 to 10)	12·9 (12·1 to 13·9)	23 (21 to 25)	16·5 (15·1 to 18·0)	27·7% (13·9 to 43·0)
		Suriname	33 (30 to 35)	12·9 (12·0 to 13·8)	107 (96 to 119)	19·0 (17·1 to 21·0)	46·7% (29·5 to 66·4)
		Trinidad and Tobago	158 (150 to 167)	18·6 (17·7 to 19·6)	373 (308 to 447)	20·9 (17·3 to 24·9)	12·4% (−7·2 to 35·7)
		Virgin Islands	23 (21 to 25)	27·7 (25·1 to 30·5)	80 (69 to 90)	43·5 (37·6 to 49·1)	57·2% (31·8 to 83·3)
	Central Latin America		7618 (7492 to 7774)	8·9 (8·8 to 9·1)	35 294 (33 818 to 36 661)	15·2 (14·6 to 15·8)	70·4% (62·5 to 77·8)
		Colombia	2012 (1933 to 2094)	11·4 (10·9 to 11·8)	8683 (7757 to 9798)	16·1 (14·4 to 18·2)	41·5% (25·3 to 60·4)
		Costa Rica	267 (255 to 279)	15·1 (14·4 to 15·7)	1397 (1264 to 1518)	28·4 (25·7 to 30·9)	88·8% (69·4 to 108·3)
		El Salvador	184 (172 to 203)	6·1 (5·7 to 6·6)	869 (731 to 1022)	15·2 (12·8 to 17·8)	149·8% (105·5 to 197·4)
		Guatemala	185 (177 to 193)	5·2 (4·9 to 5·4)	1010 (906 to 1118)	9·3 (8·4 to 10·3)	79·4% (59·3 to 101·3)
		Honduras	125 (111 to 140)	5·8 (5·1 to 6·5)	582 (443 to 718)	9·7 (7·4 to 11·9)	67·1% (29·4 to 110·8)
		Mexico	3381 (3313 to 3458)	7·7 (7·5 to 7·8)	16 550 (15 933 to 17 026)	14·5 (13·9 to 14·9)	88·9% (80·2 to 95·3)
		Nicaragua	115 (105 to 126)	7·0 (6·4 to 7·6)	501 (439 to 573)	10·9 (9·6 to 12·5)	56·8% (34·3 to 82·6)
		Panama	194 (184 to 203)	12·8 (12·2 to 13·4)	737 (673 to 802)	18·6 (17·0 to 20·2)	45·0% (31·2 to 59·6)
		Venezuela	1155 (1109 to 1204)	11·8 (11·3 to 12·2)	4965 (4272 to 5735)	17·9 (15·4 to 20·5)	52·0% (29·8 to 77·8)
	Tropical Latin America		9583 (9343 to 9871)	10·5 (10·3 to 10·8)	37 656 (36 473 to 38 850)	16·2 (15·7 to 16·8)	54·2% (46·9 to 60·5)
		Brazil	9426 (9184 to 9708)	10·6 (10·4 to 10·9)	36 934 (35 748 to 38 099)	16·3 (15·8 to 16·8)	53·5% (46·3 to 59·9)
		Paraguay	157 (143 to 171)	7·2 (6·6 to 7·9)	722 (599 to 860)	13·9 (11·5 to 16·5)	91·7% (56·2 to 131·7)
**North Africa and Middle East**							
	North Africa and Middle East		15 515 (13 256 to 19 992)	8·8 (7·6 to 11·2)	52 224 (49 748 to 54 659)	12·4 (11·8 to 12·9)	39·9% (7·3 to 65·8)
		Afghanistan	747 (306 to 1691)	10·6 (4·6 to 23·4)	1458 (806 to 2773)	12·9 (7·7 to 22·9)	21·0% (−10·0 to 101·4)
		Algeria	857 (763 to 956)	6·9 (6·1 to 7·7)	2821 (2488 to 3132)	8·6 (7·6 to 9·6)	25·7% (3·0 to 48·8)
		Bahrain	22 (20 to 25)	11·6 (10·0 to 13·6)	110 (96 to 126)	11·3 (10·0 to 12·6)	−2·8% (−20·8 to 22·0)
		Egypt	1476 (1357 to 1618)	4·9 (4·6 to 5·5)	4500 (3742 to 5195)	7·3 (6·1 to 8·4)	48·6% (16·9 to 76·2)
		Iran	2280 (1950 to 2824)	8·6 (7·4 to 10·5)	9784 (8702 to 10304)	14·0 (12·5 to 14·7)	63·3% (27·5 to 94·0)
		Iraq	631 (505 to 809)	8·1 (6·5 to 10·1)	1309 (1183 to 1430)	5·6 (5·0 to 6·0)	−31·1% (−47·4 to −12·1)
		Jordan	191 (156 to 230)	12·6 (10·2 to 15·0)	940 (782 to 1095)	15·9 (13·3 to 18·5)	26·8% (−5·6 to 65·9)
		Kuwait	63 (59 to 68)	8·2 (7·7 to 8·9)	290 (249 to 344)	10·9 (9·3 to 12·9)	32·9% (15·8 to 58·7)
		Lebanon	383 (323 to 449)	17·3 (14·6 to 20·2)	1692 (1404 to 1994)	28·1 (23·4 to 33·2)	62·7% (26·4 to 101·4)
		Libya	281 (227 to 357)	14·2 (11·6 to 17·8)	1053 (889 to 1240)	21·8 (18·4 to 25·4)	52·8% (15·2 to 102·0)
		Morocco	897 (768 to 1063)	6·2 (5·3 to 7·3)	2754 (2248 to 3298)	8·8 (7·2 to 10·5)	41·8% (3·5 to 85·6)
		Oman	53 (42 to 68)	7·5 (5·9 to 9·4)	222 (180 to 265)	10·9 (9·1 to 12·8)	46·3% (2·3 to 95·9)
		Palestine	149 (117 to 191)	16·1 (12·7 to 20·5)	432 (387 to 474)	17·0 (15·2 to 18·6)	5·5% (−22·2 to 40·8)
		Qatar	19 (15 to 24)	17·1 (14·3 to 20·8)	158 (132 to 189)	17·8 (15·0 to 21·0)	4·1% (−21·0 to 36·4)
		Saudi Arabia	438 (339 to 576)	6·7 (5·2 to 8·7)	3000 (2539 to 3528)	16·6 (14·2 to 18·9)	149·2% (76·9 to 242·9)
		Sudan	627 (407 to 1087)	6·6 (4·4 to 11·1)	1509 (1111 to 2104)	8·3 (6·3 to 11·4)	25·8% (−11·2 to 81·4)
		Syria	384 (318 to 477)	6·9 (5·8 to 8·6)	1237 (1018 to 1501)	9·7 (8·0 to 11·7)	39·5% (2·1 to 79·1)
		Tunisia	431 (378 to 499)	8·8 (7·7 to 10·1)	1476 (1164 to 1833)	12·3 (9·7 to 15·2)	40·5% (0·8 to 86·2)
		Turkey	5162 (4082 to 6691)	14·0 (11·2 to 18·0)	15 436 (13 838 to 17 433)	17·6 (15·8 to 20·0)	26·1% (−6·9 to 58·6)
		United Arab Emirates	60 (43 to 83)	13·3 (9·4 to 18·6)	759 (598 to 940)	19·9 (16·3 to 24·1)	50·3% (−0·1 to 121·4)
		Yemen	354 (189 to 634)	6·9 (4·0 to 11·8)	1234 (868 to 1820)	9·5 (6·9 to 13·5)	38·2% (−5·6 to 123·3)
**South Asia**							
	South Asia		36 162 (31 934 to 43 729)	6·2 (5·5 to 7·4)	104 958 (93 845 to 113 041)	8·1 (7·2 to 8·7)	31·6% (1·8 to 55·6)
		Bangladesh	4935 (4048 to 6317)	9·6 (8·0 to 12·3)	10 188 (8726 to 12 073)	8·4 (7·2 to 10·0)	−12·5% (−35·5 to 12·9)
		Bhutan	17 (12 to 25)	6·6 (4·9 to 9·7)	48 (36 to 62)	8·1 (6·2 to 10·3)	22·1% (−18·4 to 79·2)
		India	26 950 (23 572 to 33 017)	5·8 (5·1 to 7·0)	82 775 (74 559 to 89 201)	7·9 (7·1 to 8·6)	37·5% (6·0 to 64·0)
		Nepal	547 (373 to 843)	5·8 (4·0 to 8·8)	1438 (1157 to 1841)	7·0 (5·6 to 8·9)	20·1% (−13·0 to 63·7)
		Pakistan	3713 (3334 to 4094)	6·4 (5·7 to 7·0)	10 509 (7826 to 12 968)	9·4 (7·0 to 11·4)	47·1% (10·5 to 82·3)
**Southeast Asia, east Asia, and Oceania**							
	East Asia		114 366 (107 795 to 125 264)	12·3 (11·6 to 13·5)	462 088 (438 223 to 483 591)	22·8 (21·6 to 23·9)	85·2% (63·9 to 102·6)
		China	107 038 (100 408 to 117 587)	12·2 (11·4 to 13·4)	431 951 (408 225 to 452 721)	22·4 (21·2 to 23·5)	84·1% (62·0 to 102·2)
		North Korea	2095 (1683 to 2551)	12·2 (9·9 to 14·8)	4483 (3552 to 5524)	14·3 (11·3 to 17·5)	16·8% (−9·7 to 51·5)
		Taiwan (province of China)	3327 (3242 to 3418)	19·9 (19·4 to 20·4)	18 209 (17 062 to 19 442)	48·0 (45·1 to 51·3)	141·9% (126·3 to 158·5)
	Oceania		308 (252 to 447)	10·0 (8·5 to 14·2)	745 (617 to 1031)	11·2 (9·8 to 14·8)	12·1% (−3·2 to 27·8)
		American Samoa	4 (3 to 4)	15·9 (14·1 to 17·6)	8 (7 to 9)	18·5 (16·5 to 20·7)	16·7% (−0·5 to 38·5)
		Federated States of Micronesia	6 (5 to 8)	11·9 (9·7 to 15·5)	9 (7 to 12)	13·7 (10·9 to 16·9)	15·3% (−6·7 to 39·9)
		Fiji	34 (29 to 41)	9·3 (7·9 to 10·9)	82 (68 to 95)	11·8 (9·8 to 13·5)	26·6% (−0·4 to 57·5)
		Guam	16 (14 to 18)	18·8 (16·9 to 21·0)	43 (38 to 47)	23·8 (21·6 to 26·4)	26·5% (7·3 to 50·9)
		Kiribati	4 (3 to 4)	9·5 (8·4 to 10·5)	7 (6 to 8)	10·3 (8·5 to 12·2)	9·0% (−14·1 to 33·0)
		Marshall Islands	2 (2 to 3)	14·1 (10·6 to 20·0)	6 (4 to 7)	17·2 (13·7 to 22·2)	21·9% (2·0 to 47·8)
		Northern Mariana Islands	3 (3 to 4)	16·5 (14·2 to 19·9)	9 (8 to 10)	17·8 (15·7 to 20·0)	7·3% (−12·7 to 28·8)
		Papua New Guinea	187 (139 to 298)	9·3 (7·2 to 14·6)	469 (355 to 737)	10·0 (7·9 to 15·3)	8·0% (−11·0 to 31·5)
		Samoa	9 (7 to 11)	10·2 (8·3 to 12·9)	15 (12 to 18)	11·2 (9·2 to 13·5)	9·6% (−14·4 to 39·0)
		Solomon Islands	11 (9 to 17)	7·9 (6·2 to 11·8)	29 (23 to 38)	9·0 (7·4 to 11·7)	13·8% (−7·9 to 37·3)
		Tonga	4 (4 to 5)	7·9 (7·0 to 9·1)	7 (6 to 8)	9·4 (7·9 to 10·9)	19·4% (−6·8 to 46·8)
		Vanuatu	8 (6 to 11)	11·5 (8·9 to 16·0)	21 (16 to 28)	12·9 (9·8 to 17·1)	12·5% (−15·1 to 45·0)
	Southeast Asia		27 105 (23 553 to 32 801)	10·4 (9·1 to 12·5)	85 149 (80 680 to 90 557)	14·7 (14·0 to 15·6)	40·9% (15·4 to 63·2)
		Cambodia	572 (339 to 1013)	12·4 (7·5 to 21·6)	1445 (1086 to 1940)	13·1 (10·0 to 17·4)	5·6% (−25·7 to 56·9)
		Indonesia	7946 (6669 to 10 070)	7·8 (6·6 to 9·9)	18 739 (17 443 to 20 172)	9·3 (8·6 to 10·0)	18·0% (−9·8 to 43·7)
		Laos	251 (154 to 408)	11·9 (7·5 to 19·1)	488 (377 to 650)	11·7 (9·2 to 15·4)	−1·6% (−29·0 to 40·8)
		Malaysia	1800 (1555 to 2184)	20·5 (17·5 to 24·7)	6605 (5777 to 7568)	26·9 (23·6 to 30·6)	31·1% (5·5 to 55·9)
		Maldives	7 (5 to 12)	7·8 (5·3 to 11·9)	27 (24 to 31)	9·1 (7·9 to 10·2)	15·6% (−31·1 to 78·3)
		Mauritius	74 (70 to 78)	9·9 (9·4 to 10·5)	322 (294 to 351)	19·4 (17·6 to 21·1)	95·4% (76·0 to 115·6)
		Myanmar	3328 (1899 to 5522)	14·2 (8·3 to 23·3)	6560 (4900 to 9053)	14·9 (11·2 to 20·6)	5·4% (−21·4 to 55·0)
		Philippines	2039 (1903 to 2160)	6·5 (6·1 to 6·9)	13 472 (11 799 to 15 373)	18·4 (16·1 to 20·8)	181·9% (145·4 to 227·0)
		Sri Lanka	654 (610 to 705)	6·0 (5·6 to 6·4)	2451 (1929 to 2976)	10·1 (8·0 to 12·2)	69·4% (32·7 to 110·5)
		Seychelles	9 (8 to 10)	15·0 (13·6 to 17·9)	39 (35 to 42)	36·4 (32·6 to 39·8)	142·4% (84·6 to 176·7)
		Thailand	4671 (4283 to 5179)	12·4 (11·3 to 13·7)	15 598 (13 999 to 17 415)	16·0 (14·3 to 17·8)	29·3% (10·2 to 47·1)
		Timor-Leste	20 (15 to 32)	7·1 (5·4 to 10·6)	79 (63 to 102)	10·2 (8·1 to 13·0)	43·1% (1·8 to 94·3)
		Vietnam	5697 (4925 to 6498)	13·8 (12·0 to 15·8)	19 210 (16 530 to 22 243)	21·0 (18·2 to 24·1)	51·9% (21·3 to 87·3)
**Sub-Saharan Africa**							
	Central sub-Saharan Africa		1904 (1499 to 2534)	8·7 (7·2 to 11·1)	4416 (3711 to 5434)	9·2 (7·9 to 10·9)	5·2% (−11·9 to 25·7)
		Angola	373 (246 to 563)	9·7 (6·8 to 14·0)	1049 (857 to 1289)	10·3 (8·4 to 12·4)	6·0% (−26·2 to 53·2)
		Central African Republic	111 (64 to 180)	9·8 (6·1 to 15·2)	202 (115 to 335)	9·8 (6·1 to 15·4)	0·0% (−20·2 to 24·5)
		Congo (Brazzaville)	129 (96 to 171)	12·2 (9·5 to 15·4)	300 (234 to 386)	12·8 (10·3 to 15·7)	5·2% (−18·2 to 38·8)
		Democratic Republic of the Congo	1205 (953 to 1563)	8·0 (6·6 to 10·1)	2676 (2098 to 3493)	8·4 (6·8 to 10·5)	4·2% (−15·7 to 29·5)
		Equatorial Guinea	19 (11 to 31)	9·9 (6·4 to 15·2)	54 (35 to 78)	12·2 (8·0 to 17·1)	22·1% (−35·7 to 107·4)
		Gabon	66 (51 to 88)	12·0 (9·4 to 15·5)	134 (101 to 168)	13·2 (9·9 to 16·3)	10·2% (−21·1 to 42·6)
	Eastern sub-Saharan Africa		7703 (6131 to 9924)	10·5 (8·6 to 13·3)	16 007 (14 839 to 17 000)	10·7 (9·9 to 11·3)	1·2% (−20·0 to 26·6)
		Burundi	184 (141 to 247)	8·7 (6·7 to 11·4)	328 (258 to 429)	8·4 (6·8 to 10·8)	−2·8% (−22·1 to 22·1)
		Comoros	24 (19 to 31)	11·8 (9·4 to 15·4)	52 (43 to 64)	12·0 (9·8 to 14·6)	1·9% (−20·9 to 34·3)
		Djibouti	20 (13 to 30)	13·4 (8·9 to 19·9)	77 (53 to 109)	14·4 (10·2 to 19·9)	7·2% (−25·2 to 60·7)
		Eritrea	124 (87 to 180)	13·0 (9·5 to 18·7)	320 (248 to 412)	14·3 (11·4 to 17·9)	9·9% (−20·3 to 58·5)
		Ethiopia	2566 (1412 to 3859)	14·1 (8·3 to 20·6)	4375 (3873 to 4821)	11·7 (10·4 to 12·8)	−17·1% (−44·2 to 45·3)
		Kenya	680 (565 to 832)	8·2 (6·8 to 10·1)	1966 (1754 to 2229)	9·6 (8·6 to 10·9)	17·5% (2·2 to 32·4)
		Madagascar	527 (403 to 726)	10·1 (7·7 to 13·7)	1021 (771 to 1385)	10·0 (7·6 to 13·4)	−1·1% (−19·1 to 22·1)
		Malawi	194 (128 to 235)	5·0 (3·5 to 5·9)	438 (352 to 523)	6·1 (4·9 to 7·2)	22·3% (−5·5 to 79·8)
		Mozambique	697 (599 to 803)	12·1 (10·4 to 13·9)	1503 (1236 to 1830)	14·4 (12·1 to 17·3)	19·2% (−9·8 to 55·4)
		Rwanda	239 (179 to 322)	8·4 (6·2 to 11·2)	448 (285 to 604)	8·2 (5·3 to 11·0)	−2·0% (−25·6 to 28·8)
		Somalia	281 (144 to 472)	11·2 (6·4 to 18·3)	766 (495 to 1188)	12·7 (8·4 to 19·4)	13·6% (−17·4 to 72·5)
		South Sudan	244 (146 to 383)	10·5 (6·7 to 15·9)	402 (283 to 588)	11·1 (7·8 to 16·0)	5·7% (−22·6 to 52·3)
		Tanzania	1055 (805 to 1321)	10·2 (8·2 to 12·7)	2307 (1954 to 2715)	10·1 (8·6 to 11·8)	−1·3% (−25·3 to 27·9)
		Uganda	504 (430 to 588)	7·7 (6·6 to 9·0)	1263 (1044 to 1499)	9·5 (7·9 to 11·2)	22·1% (−2·8 to 53·2)
		Zambia	361 (287 to 444)	13·1 (10·7 to 15·8)	731 (622 to 841)	12·2 (10·4 to 13·9)	−7·0% (−30·2 to 21·9)
	Southern sub-Saharan Africa		2591 (2398 to 2816)	9·3 (8·5 to 10·2)	6002 (5469 to 6404)	11·1 (10·1 to 11·8)	19·1% (10·4 to 26·8)
		Botswana	51 (42 to 61)	9·1 (7·6 to 10·7)	134 (111 to 170)	10·5 (8·8 to 13·0)	15·9% (−10·1 to 44·4)
		Lesotho	70 (58 to 95)	7·4 (6·1 to 9·8)	122 (95 to 153)	10·7 (8·4 to 13·3)	43·8% (11·1 to 81·6)
		Namibia	56 (46 to 72)	7·8 (6·5 to 10·0)	118 (100 to 139)	8·6 (7·3 to 10·1)	9·8% (−21·3 to 45·5)
		South Africa	1989 (1803 to 2212)	9·3 (8·3 to 10·5)	4774 (4223 to 5154)	11·1 (9·8 to 12·0)	18·7% (10·5 to 27·2)
		Swaziland (eSwatini)	30 (25 to 39)	10·7 (8·9 to 13·5)	72 (56 to 91)	13·3 (10·5 to 16·4)	24·8% (−6·0 to 59·6)
		Zimbabwe	395 (349 to 446)	9·7 (8·6 to 10·9)	782 (667 to 915)	11·7 (10·0 to 13·6)	21·0% (1·3 to 44·7)
		Western sub-Saharan Africa	6983 (5677 to 9178)	8·2 (6·7 to 10·7)	15 089 (12 862 to 17 883)	9·0 (7·7 to 10·5)	8·8% (−18·3 to 39·8)
		Benin	132 (105 to 159)	6·6 (5·3 to 7·9)	350 (280 to 444)	7·9 (6·4 to 9·9)	20·0% (−4·6 to 53·3)
		Burkina Faso	508 (408 to 592)	12·3 (10·1 to 14·3)	1146 (955 to 1366)	13·7 (11·5 to 16·1)	11·2% (−13·0 to 43·3)
		Cameroon	365 (308 to 425)	8·5 (7·2 to 9·9)	1015 (746 to 1290)	9·4 (7·0 to 11·9)	11·3% (−13·6 to 37·9)
		Cape Verde	9 (8 to 10)	4·0 (3·6 to 4·4)	40 (36 to 44)	8·9 (8·0 to 9·8)	124·9% (95·5 to 160·7)
		Chad	182 (141 to 252)	6·5 (5·0 to 8·9)	422 (320 to 564)	8·2 (6·3 to 10·8)	27·1% (5·0 to 54·9)
		Côte d'Ivoire	220 (193 to 250)	5·7 (5·0 to 6·4)	554 (438 to 698)	5·8 (4·7 to 7·3)	2·5% (−21·4 to 31·0)
		The Gambia	20 (17 to 24)	5·9 (5·0 to 6·9)	60 (43 to 80)	6·6 (4·8 to 8·8)	12·8% (−26·3 to 56·6)
		Ghana	484 (397 to 613)	7·9 (6·6 to 9·8)	1384 (1113 to 1656)	9·5 (7·6 to 11·2)	19·5% (−18·0 to 58·4)
		Guinea	198 (175 to 221)	6·0 (5·4 to 6·7)	403 (323 to 512)	7·7 (6·2 to 9·7)	28·7% (1·5 to 64·7)
		Guinea-Bissau	45 (24 to 72)	11·4 (6·4 to 17·9)	72 (50 to 98)	10·8 (7·8 to 14·5)	−5·0% (−29·9 to 37·4)
		Liberia	87 (69 to 115)	7·8 (6·2 to 10·2)	164 (122 to 230)	9·0 (6·8 to 12·5)	15·8% (−8·9 to 45·2)
		Mali	298 (264 to 339)	7·5 (6·7 to 8·5)	625 (454 to 856)	7·8 (5·7 to 10·6)	3·8% (−26·6 to 44·8)
		Mauritania	92 (68 to 126)	9·1 (6·8 to 12·4)	181 (138 to 234)	9·5 (7·3 to 12·2)	4·6% (−20·3 to 44·4)
		Niger	183 (127 to 267)	6·7 (4·7 to 9·7)	449 (327 to 650)	6·6 (4·9 to 9·5)	−1·0% (−18·3 to 19·3)
		Nigeria	3623 (2486 to 5445)	8·5 (5·9 to 12·7)	7096 (5194 to 9621)	9·2 (6·9 to 12·3)	8·5% (−27·9 to 65·9)
		São Tomé and Príncipe	6 (5 to 6)	8·4 (7·5 to 9·5)	13 (10 to 18)	13·8 (10·6 to 17·9)	63·1% (22·4 to 115·0)
		Senegal	293 (231 to 381)	9·3 (7·3 to 12·0)	564 (438 to 697)	8·3 (6·5 to 10·2)	−10·8% (−39·8 to 28·9)
		Sierra Leone	158 (113 to 220)	8·2 (5·9 to 11·3)	303 (229 to 403)	9·3 (7·1 to 12·3)	14·1% (−9·6 to 44·1)
		Togo	80 (63 to 96)	6·5 (5·2 to 7·9)	247 (190 to 316)	7·7 (6·0 to 9·6)	17·3% (−4·9 to 43·2)

Data in parentheses are 95% uncertainty intervals.

**Figure 1:**
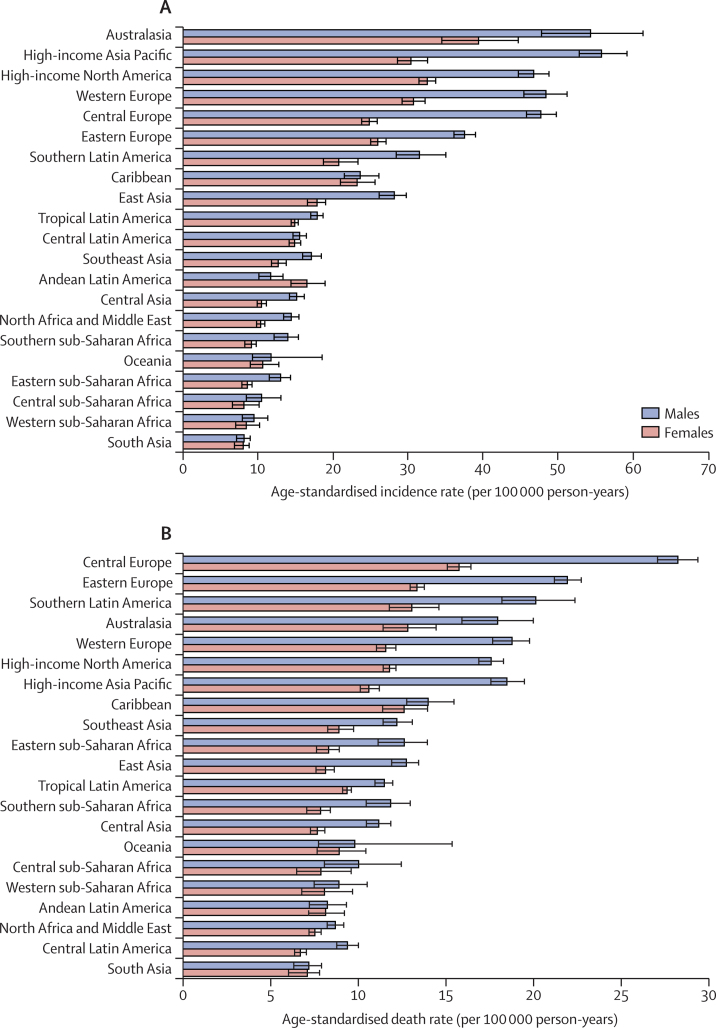
The age-standardised incidence (A) and death (B) rates of colorectal cancer for 21 GBD regions by sex, 2017 Error bars indicate 95% uncertainty intervals. GBD=Global Burden of Diseases, Injuries, and Risk Factors Study.

Australasia (46·4 [95% UI 42·5–50·6] per 100 000 person-years), high-income Asia Pacific (41·9 [40·2–44·1] per 100 000 person-years), and high-income North America (39·1 [37·9–40·3] per 100 000 person-years) had the highest age-standardised incidence rates in 2017. By contrast, south Asia (8·1 [7·2–8·7] per 100 000 person-years), western sub-Saharan Africa (9·0 [7·7–10·5] per 100 000 person-years), and central sub-Saharan Africa (9·2 [7·9–10·9] per 100 000 person-years) had the lowest age-standardised incidence rates in 2017 (table). In all regions except Andean Latin America, the age-standardised incidence rate was higher among males than females in 2017 (figure 1A). The age-standardised death rates in 2017 were highest in central Europe (20·9 [20·3–21·6] per 100 000 person-years), eastern Europe (16·4 [16·0–16·9] per 100 000 person-years), and southern Latin America (16·1 [14·9–17·4] per 100 000 person-years). By contrast, south Asia (7·1 [6·4–7·6] per 100 000 person-years), north Africa and the Middle East (8·0 [7·6–8·3] per 100 000 person-years), and central Latin America (8·0 [7·7–8·3] per 100 000 person-years) had the lowest age-standardised death rates in 2017 (appendix pp 21–29). The age-standardised death rates in 2017 were higher for males in all GBD regions (figure 1B).

**Figure 2:**
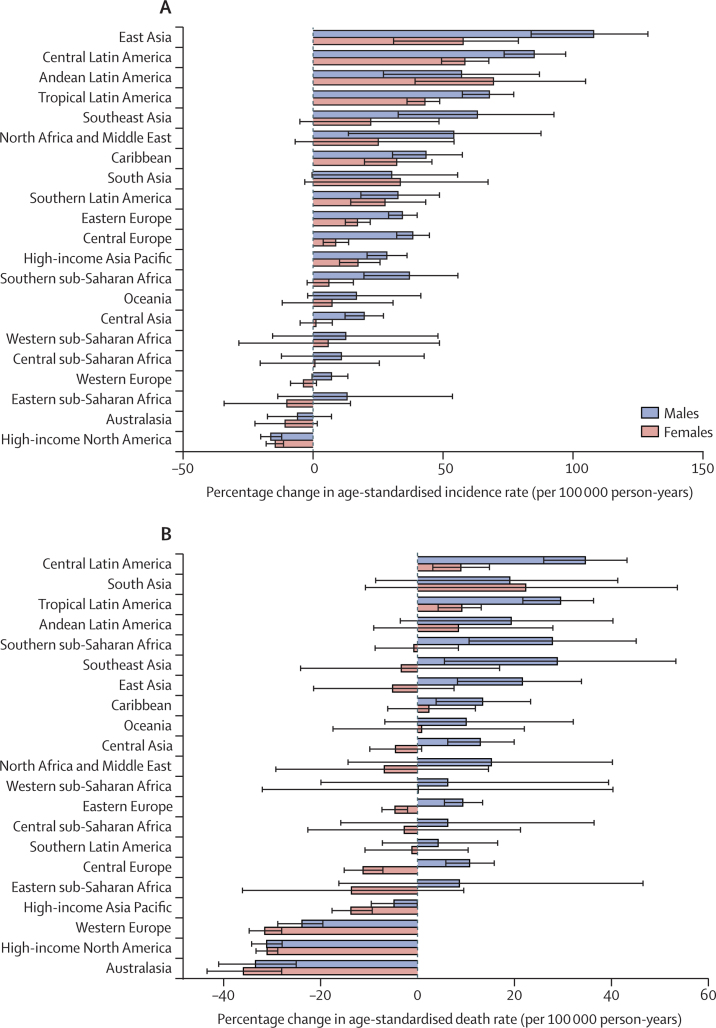
The percentage change in age-standardised incidence (A) and death (B) rates of colorectal cancer for 21 GBD regions by sex, 1990–2017 Error bars indicate 95% uncertainty intervals. GBD=Global Burden of Diseases, Injuries, and Risk Factors Study.

The percentage change in age-standardised incidence rates from 1990 to 2017 differed substantially between the GBD regions, with east Asia (85·2% [95% UI 63·9 to 102·6]), central Latin America (70·4% [62·5 to 77·8]), and Andean Latin America (64·3% [41·6 to 89·3]) showing the largest increases. By contrast, high-income North America (−14·2% [–17·2 to −11·4]) and Australasia (−7·4% [–15·7 to 1·0]) showed decreasing trends during this period, although the decrease for Australasia was not significant (table). The percentage change in age-standardised death rates from 1990 to 2017 also differed between the GBD regions. The largest increases were seen in south Asia (20·4% [–6·2 to 42·8]), central Latin America (20·4% [15·0 to 25·4]), and tropical Latin America (18·2% [12·9 to 22·6]). By contrast, the largest decreases during this period were found in Australasia (−34·0% [–39·2 to −28·6]), high-income North America (−30·0% [–32·2 to −27·8]), and western Europe (−26·1% [–29·1 to −23·1]; appendix pp 21–29). Percentage change increments in age-standardised incidence rates of colorectal cancer from 1990 to 2017 were higher among males in most regions except Andean Latin America and south Asia (figure 2A). Similarly, percentage change increments for colorectal cancer age-standardised death rates in this period were highest in males in most regions, except for south Asia (figure 2B). In 2017, the highest number of incident cases were found in east Asia, western Europe, and high-income North America (table; appendix p 1). The highest numbers of deaths were in east Asia, western Europe, and high-income North America in 2017 (appendix pp 2, 21–28).

**Figure 3:**
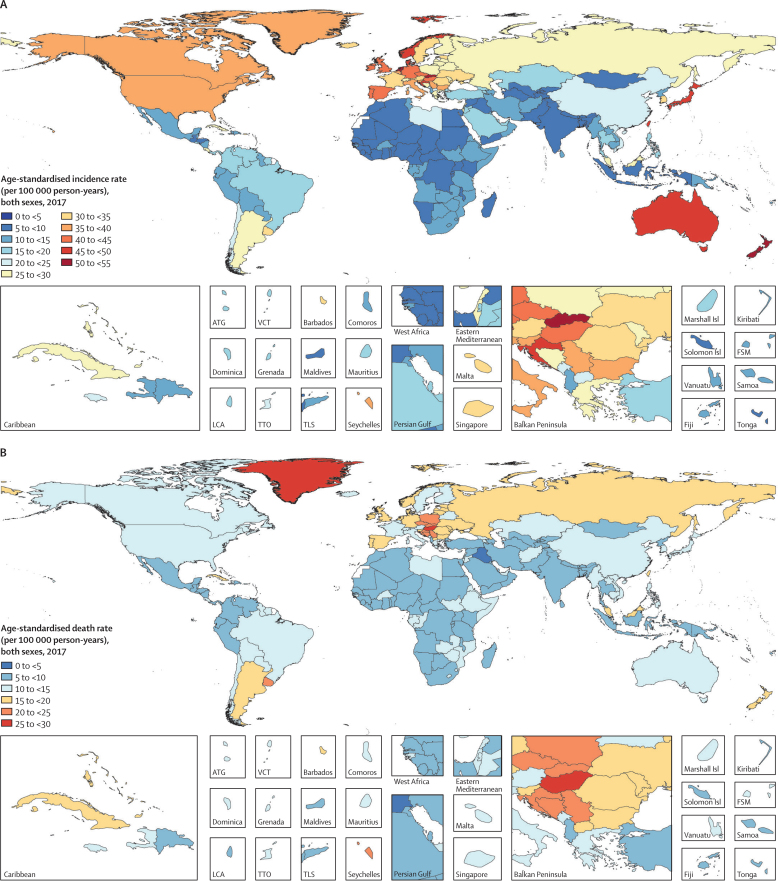
Age-standardised incidence (A) and death (B) rate of colorectal cancer per 100 000 person-years by country and territory, 2017 ATG=Antigua and Barbuda. FSM=Federated States of Micronesia. Isl=Islands. LCA=Saint Lucia. TLS=Timor-Leste. TTO=Trinidad and Tobago. VCT=Saint Vincent and the Grenadines.

In 2017, the age-standardised incidence rates for colorectal cancer were highest in Slovakia (52·4 [95% UI 47·5–57·1] per 100 000 person-years), the Netherlands (50·9 [47·1–54·7] per 100 000 person-years), and New Zealand (50·2 [46·6–54·2] per 100 000 person-years). The lowest age-standardised rates in 2017 were found in Iraq (5·6 [5·0–6·0] per 100 000 person-years), Côte d'Ivoire (5·8 [4·7–7·3] per 100 000 person-years), and Malawi (6·1 [4·9–7·2] per 100 000 person-years; figure 3A; table). In 2017, the age-standardised death rates were highest in Greenland (26·5 [24·2–28·8] per 100 000 person-years), Hungary (26·1 [24·5–27·8] per 100 000 person-years), and Slovakia (24·5 [21·9–26·4] per 100 000 person-years). Conversely, Iraq (4·5 [4·1–4·9] per 100 000 person-years), Maldives (5·1 [4·4–5·7] per 100 000 person-years), and Egypt (5·3 [4·3–6·1] per 100 000 person-years) had the lowest age-standardised death rates in 2017 (figure 3B; appendix pp 21–29).

The percentage change in age-standardised incidence rates from 1990 to 2017 differed substantially between countries, with the Philippines (181·9% [95% UI 145·4 to 227·0]), El Salvador (149·8% [105·5 to 197·4]), and Saudi Arabia (149·2% [76·9 to 242·9]) showing the largest increases. By contrast, Kyrgyzstan (−31·5% [–37·3 to −22·6]), Iraq (−31·1% [–47·4 to −12·1]), and Austria (−22·6% [–28·6 to −16·3]) showed the largest decreases in age-standardised incidence during this period (table). The percentage change in age-standardised death rates from 1990 to 2017 also differed between countries. The largest increases were seen in the Philippines (139·8% [109·4 to 176·2]), Cape Verde (108·5% [80·7 to 143·9]), and Seychelles (82·9% [40·7 to 107·9]). By contrast, the largest decreases during this period were found in Austria (−42·7% [–46·7 to −38·8]), the Czech Republic (−38·3% [–42·6 to −33·4]), and Singapore (−37·5% [–42·2 to −31·8]; appendix pp 21–29).

**Figure 4:**
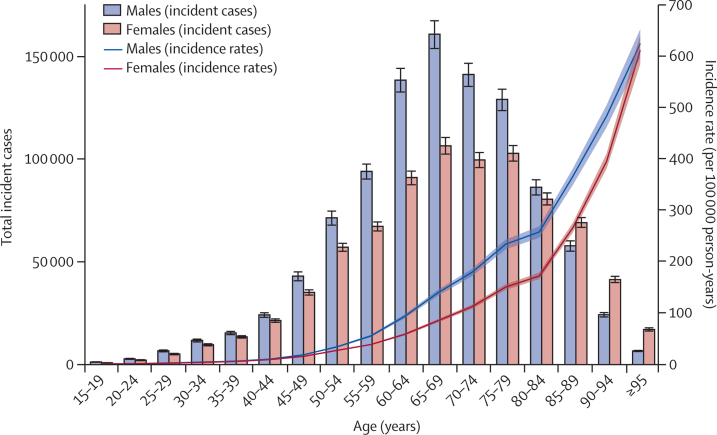
Global number of incident cases and incidence rate of colorectal cancer per 100 000 person-years by age and sex, 2017 Error bars indicate the 95% uncertainty interval for incident cases. Shading indicates the 95% uncertainty interval for the incidence rate.

Our study found that, in 2017, the incidence rate increased in a non-linear manner with increasing age and was higher in males than in females across all age groups (figure 4). The difference in incidence rates between males and females increased with each increasing age group up to the ages of 85–89 years, after which the gap started to decrease again. The number of incident cases was also higher in males than in females up to the ages of 80–84 years and peaked at ages 65–69 years (figure 4). A relatively similar pattern was also observed for death rates and death counts (appendix p 3). The highest rates of incidence and death observed were in the oldest age group (≥95 years) for both sexes in 2017. The pattern for DALY rates was slightly different, such that the age-standardised DALY rate started decreasing after the ages of 80–84 years for males and after the ages of 85–89 years for females (appendix p 4). The number of DALYs was also higher in males than in females up to the ages of 80–84 years, and then females had slightly higher numbers of DALYs for the older age groups. The number of DALYs followed a normal distribution and peaked at ages 65–69 years (appendix p 4). Decomposition of the DALY rate into YLLs and YLDs showed that YLLs were the primary contributor to DALYs, with the 2017 YLL rate peaking at the ages of 80–84 years (appendix p 5).

**Figure 5:**
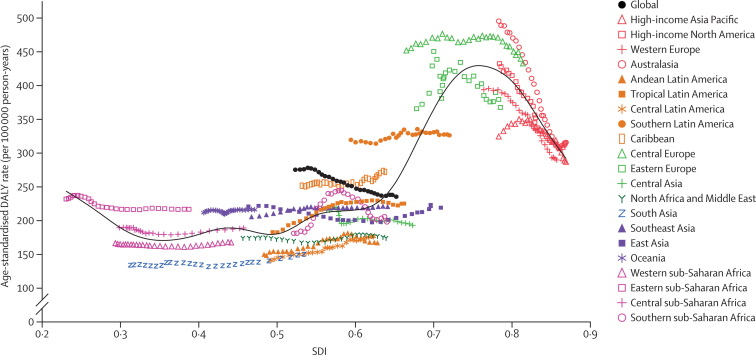
Age-standardised DALY rates per 100 000 person-years for colorectal cancer for 21 GBD regions by SDI, 1990–2017 Expected values based on SDI and age-standardised DALY rates in all locations are shown as the black line. For each region, points from left to right depict estimates from each year from 1990 to 2017. DALY=disability-adjusted life-year. GBD=Global Burden of Diseases, Injuries, and Risk Factors Study. SDI=Socio-demographic Index.

Figure 5 presents the global and regional-level observed age-standardised DALY rates from 1990 to 2017 versus the expected level based only on the SDI values of the global regions. The expected pattern was non-linear in nature, peaking at an SDI value of approximately 0·75, before decreasing with increasing SDI values. However, there were large regional differences. Australasia, central Europe, western Europe, and high-income North America showed the largest decreases in observed age-standardised DALY rates with increases in SDI value, whereas the Caribbean and central Latin American regions showed increases in observed age-standardised DALY rates with increasing SDI value. The observed age-standardised DALY rate for some regions, such as southern sub-Saharan Africa, initially increased and then decreased with an improvement in SDI value over time. At the global level, the age-standardised DALY rate dropped below the expected level for 2015–17.

**Figure 6:**
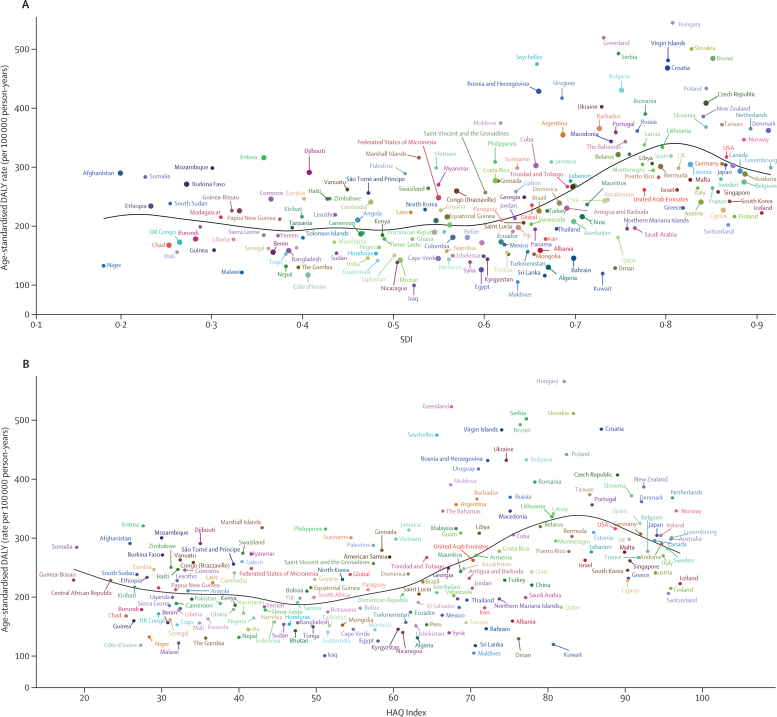
Age-standardised DALY rates of colorectal cancer for 195 countries and territories by SDI and HAQ Index, 2017 (A) Age-standardised DALY rates of colorectal cancer by 195 countries and the HAQ Index, 2016. (B) Expected values are shown as the black line. DALY=disability-adjusted life-year. HAQ Index=Healthcare Access and Quality Index. SDI=Socio-demographic Index.

Figure 6 shows the national-level observed age-standardised DALY rates and their association with the SDI and HAQ Index. The expected patterns were non-linear in nature, peaking at an SDI value of approximately 0·81 and HAQ Index value of approximately 84, before decreasing with increasing SDI and HAQ Index values. However, there were large national differences. Several countries, including Hungary, Greenland, Slovakia, Serbia, and Brunei, had a higher than expected age-standardised DALY rate, whereas others, such as Iraq, Maldives, Sri Lanka, Kuwait, and Oman, had much lower than expected age-standardised DALY rates based only on the SDI. This pattern was also observed based on the HAQ Index.

**Figure 7:**
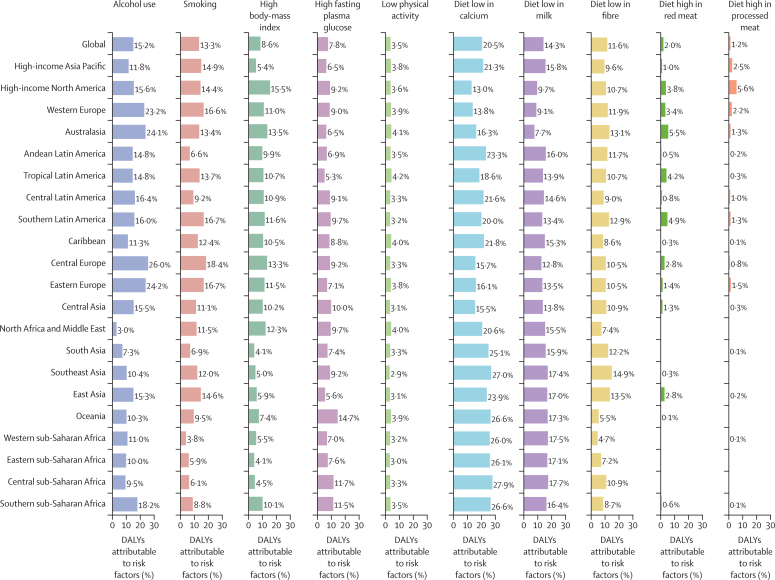
Percentage of age-standardised DALYs due to colorectal cancer attributable to risk factors for 21 GBD regions, both sexes, 2017 DALY=disability-adjusted life-year. GBD=Global Burden of Diseases, Injuries, and Risk Factors Study.

Although the proportions of age-standardised DALYs that were attributable to colorectal cancer risk factors differed in the GBD regions, diet low in calcium (20·5% [95% UI 12·9–28·9]), alcohol use (15·2% [12·1–18·3]), and diet low in milk (14·3% [5·1–24·8]) had the three highest percentages of attributable age-standardised DALYs for both sexes globally (figure 7; appendix p 11). This global pattern was different in males and females: alcohol use (21·5% [17·4–25·9]), diet low in calcium (19·8% [12·3–28·2]), and smoking (19·2% [12·8–25·3]) were the risk factors that contributed most to age-standardised DALYs in males, whereas diets low in calcium (21·3% [13·7–29·9]), milk (14·4% [5·1–24·0]), and fibre (12·5% [6·6–19·3]) were the risk factors that contributed most to age-standardised DALYs in females (appendix pp 6–7). The percentage of DALYs attributable to colorectal cancer risk factors also differed across age groups, especially for alcohol use, smoking, and high fasting plasma glucose. The highest percentage of global attributable DALYs were in the 55–59 years age group for alcohol use, 65–69 years age group for smoking, and 85–89 years age group for high fasting plasma glucose for both sexes combined (appendix p 8). The sex-specific estimates of global DALYs attributable to studied risk factors by age are reported in appendix (pp 9–10).

## Discussion

From 1990 to 2017, the age-standardised incidence rates of colorectal cancer increased globally, with substantial regional and national heterogeneity. By contrast, the age-standardised death and DALY rates decreased across the study period. On the basis of our DALY estimates, colorectal cancer is the 36th leading cause of disease burden globally for 2017, and is the fourth leading cause of cancer burden, behind only lung cancer, liver cancer, and stomach cancer.

The most recent GLOBOCAN report^3^ in 2018 estimated that there were 1 800 977 incident cases and 861 663 deaths from colorectal cancer, which are relatively consistent with our 2017 estimates (1 833 451 [95% UI 1 791 865–1 873 464] incident cases and 896 040 [876 279–915 720] deaths). Similar to the GLOBOCAN report,^3^ we found that the highest age-standardised incidence rates in 2017 were in Australasia, high-income Asia Pacific, and high-income North America, and the highest age-standardised death rates were found in central Europe, eastern Europe, and southern Latin America.

We also investigated heterogeneous trends in age-standardised incidence, death, and DALY rates from 1990 to 2017 at the national level. Most countries showed an increase in the age-standardised incidence rate of colorectal cancer during 1990–2017, such that only Australasia and high-income North America experienced a decrease in age-standardised incidence rate at the regional level. One potential explanation for this global increase in age-standardised incidence is that the introduction of screening tests might have led to increased detection and thus increased incidence, but this increase might be short-lived because of the removal of precancerous polyps during colonoscopies.^5^ Similarly, in countries where screening programmes were established two or three decades ago, reductions in death rates were observed that support the benefits attributable to screening interventions.^28^ Improving survival by adopting the best practices in cancer treatment and management can also lead to reduced death rates. On the basis of the data from high-income countries, several factors might have contributed to the decrease in the number of deaths due to colorectal cancer, such as enhanced access to screening colonoscopy and early stage detection, as well as improved surgical techniques, radiotherapy, chemotherapy, targeted therapy, and palliative care.^29^, ^30^, ^31^, ^32^ Key interventions to decrease deaths from colorectal cancer include the removal of polyps and early detection interventions, such as colonoscopy, flexible sigmoidoscopy, faecal occult blood testing, and faecal immunochemical testing.

Previous research has investigated the association between a country's development level and the incidence and mortality rates of colorectal cancer, using the Human Development Index.^7^ Because one of the components of the Human Development Index is health related, it is not optimal to use this index when comparing the health outcomes of countries. To avoid this problem, we used the SDI, which does not contain any health-related measures. Our analysis of the association between the SDI and age-standardised DALY rate of colorectal cancer produced results that have not been previously reported. In some regions, such as Australasia and central Europe, the age-standardised DALY rates were higher than expected from 1990 to 2017, whereas in other regions they were lower than expected, such as in central Latin America and south Asia. Several regions also fluctuated between higher and lower than expected age-standardised DALY rates during the study period. Therefore, regional trends in age-standardised rates of deaths, DALYs, and incidence should not just be considered in isolation. Instead, their observed rates should be compared with their expected rates to determine whether regions have managed colorectal cancer better or worse than expected. Additionally, dividing regions or countries into developed and undeveloped regions to evaluate the association between development and colorectal cancer burden might be an oversimplification, since the findings from this study suggest that the association is complex and non-linear in nature. Furthermore, at the national level, age-standardised DALY rates of countries on both ends of the SDI range were at higher than expected levels based on the SDI, so countries at all development levels need to enhance their prevention programmes.

Our report indicates that the colorectal cancer burden attributable to risk factors is different in males and females, and this difference should be considered in national policy makers' prevention programmes. Alcohol use, smoking, and diets low in calcium, milk, and fibre had considerable attributable colorectal cancer burden in males. By contrast, dietary risks, but not alcohol use or smoking, were found to have considerable attributable burden in females. The results of this study highlight the role of certain dietary risk factors, which are responsible for a greater burden than smoking or alcohol use globally. Specifically, diet low in calcium has been previously described as a risk factor for colorectal cancer.^33^ However, the large burden attributable to this dietary risk factor has not been described before at the global level. This large burden attributable to a diet low in calcium is likely due in part to the high prevalence of this risk factor. The results of our study underscore the importance of improving diet through public health interventions.

A previous study showed that alcohol use is responsible for nearly 10% of global deaths in the population aged 15–49 years and will lead to remarkable health loss in the absence of appropriate policy action.^34^ The same study also reported that the safest level of alcohol consumption is zero, which is in contrast to current health guidelines.^34^ Decreasing population-level alcohol consumption should be considered in prevention strategies to effectively minimise the corresponding health loss.^34^

Smoking is another important risk factor. The global prevalence of smoking has decreased by 28% in males and 34% in females since 1990.^18^ Taxation, advertising bans, and educational programmes about smoking and its toll on health are suggested as strategies to decrease smoking prevalence more substantially.^18^

Diets low in calcium, milk, and fibre should also be addressed in colorectal cancer prevention strategies along with addressing low physical activity. To improve diet, increase physical activity, and reduce smoking, the American Heart Association suggests following population-based approaches, such as media and educational campaigns; labelling and consumer information; taxation, subsidies, and other economic incentives; school and workplace approaches; local environmental changes; and direct restrictions and mandates.^35^

Our findings indicate that although high body-mass index was not among the top three risk factors for attributable DALYs, it is an important risk factor that has a higher attributable percentage of DALYs due to colorectal cancer in males than in females. One study showed that the prevalence of obesity has doubled in more than 70 countries and has continuously increased in most other countries since 1980.^36^ Effective prevention programmes are needed to decrease exposure to this important risk factor through appropriate strategies, such as restricting the advertisement of unhealthy foods, using taxation to reduce consumption of unhealthy foods, providing subsidies to increase intake of healthy foods, and using supply-chain incentives to increase the production of healthy foods.^37^ Fasting plasma glucose can also be controlled mainly through physical activity and healthy diets.^38^

This study had several limitations, including the fact that some of the variations in age-standardised incidence and mortality rates might be due to detection biases as well as changes in screening protocols. For example, the low age-standardised incidence and death rates in Iraq might be due to low detection rates. In addition, a major limitation of cancer burden research is the scarcity of data for many countries. Although different data sources, such as cancer registries, vital registration systems, and verbal autopsies, are used to produce cancer estimates, some countries do not have any of these sources available so their estimates are based on predictive covariates or trends from neighbouring countries. Moreover, estimates for the most recent years are usually based on past trends and covariates because there is a lag in data availability. As GBD is an iterative study, additional data sources for different locations will be added in future rounds and make the estimates more data driven, particularly in data-sparse locations.

This study found large country and regional variations in the burden of colorectal cancer in 2017. Whereas age-standardised incidence rates increased in most countries and territories over the measurement period, age-standardised death rates decreased at the global level, and in particular in high SDI countries, possibly due to fast improvement in diagnostics and interventions in these countries. Further research is required to expand our knowledge of additional factors associated with colorectal cancer incidence and to improve early detection and treatment of this disease, especially in developing countries. Clearly, colorectal cancer remains a substantial public health challenge across the globe. The results of GBD 2017 can be valuable for policy makers to implement cost-effective interventions and address modifiable risk factors and for researchers to design and carry out further research on proper modalities for prevention, early detection, and treatment of colorectal cancer.

**This online publication has been corrected. The corrected version first appeared at thelancet.com/gastrohep on Feb 12, 2020**
