# Correction to *Lancet Gastroenterol Hepatol* 2019; 4: 913–33

**DOI:** 10.1016/S2468-1253(20)30017-0

**Published:** 2020-02-12

*GBD 2017 Colorectal Cancer Collaborators. The global, regional, and national burden of colorectal cancer and its attributable risk factors in 195 countries and territories, 1990–2017: a systematic analysis for the Global Burden of Disease Study 2017*. Lancet Gastroenterol Hepatol *2019; **4:** 913–33*—In this Article, the affiliation for C La Vecchia should read “Clinical Medicine and Community Health, University of Milan, Milano, Italy” and the affiliation for M Jafarnia should read “Department of Immunology, School of Medicine, Isfahan University of Medical Sciences, Isfahan, Iran”. The affiliation for N Baheiraei should read “Faculty of Medical Sciences, Tarbiat Modares University, Tehran, Iran”. These changes have been made to the online version as of Feb 12, 2020.

