# Supplementary material for: The global, regional, and national burden of colorectal cancer and its attributable risk factors in 195 countries and territories, 1990–2017: a systematic analysis for the Global Burden of Disease Study 2017
Source: Lancet Gastroenterol Hepatol. 2019 Oct 21;4(12):913–33. doi: 10.1016/S2468-1253(19)30345-0 (PMC7026697; doi:10.1016/S2468-1253(19)30345-0)
Supplement: Supplementary appendix [file mmc1.pdf]

THE LANCET
Gastroenterology & Hepatology

Supplementary appendix

This appendix formed part of the original submission and has been peer reviewed. We post it as supplied by the authors.

Supplement to: GBD 2017 Colorectal Cancer Collaborators. The global, regional, and national burden of colorectal cancer and its attributable risk factors in 195 countries and territories, 1990–2017: a systematic analysis for the Global Burden of Disease Study 2017. *Lancet Gastroenterol Hepatol* 2019; published online Oct 21. http://dx.doi.org/10.1016/S2468-1253(19)30345-0.

Supplementary appendix

Supplement to: The global, regional, and national burden of colorectal cancer and its attributable risk factors in 195 countries and territories, 1990–2017: a systematic analysis for the Global Burden of Disease Study 2017

Table of Contents:

Appendix Figures

Appendix Figure 1: Number of incident cases of colorectal cancer from 1990 to 2017 for 21 Global Burden of Disease regions

Appendix Figure 2: Number of deaths of colorectal cancer from 1990 to 2017 for 21 Global Burden of Disease regions

Appendix Figure 3: Global number of deaths and death rate of colorectal cancer per 100 000 population by age and sex, 2017

Appendix Figure 4: Global number of DALYs and DALY rate of colorectal cancer per 100, 000 population by age and sex, 2017

Appendix Figure 5: Global number of YLLs and YLDs and rate of YLLs and YLDs per 100 000 population due to colorectal cancer by age, 2017

Appendix Figure 6: Percentage of DALYs due to colorectal cancer attributable to risk factors for 21 Global Burden of Disease regions, 2017, males.

Appendix Figure 7: Percentage of DALYs due to colorectal cancer attributable to risk factors for 21 Global Burden of Disease regions, 2017, females.

Appendix Figure 8: Percentage of DALYs due to colorectal cancer attributable to risk factors by age for both sexes, 2017

Appendix Figure 9: Percentage of DALYs due to colorectal cancer attributable to risk factors by age for males, 2017

Appendix Figure 10: Percentage of DALYs due to colorectal cancer attributable to risk factors by age for females, 2017

Appendix Figure 11: Percentage and UIs of DALYs due to colorectal cancer attributable to risk factors for 21 GBD regions, both sexes, 2017.

Appendix Tables

Appendix Table 1: Data quality rating from 0 to 5 stars, maximum percent well-certified per 5-year interval and percent well-certified across time series for 195 countries, 1980–2017

Appendix Table 2: Covariates used in Cause of Death Ensemble model and assumed direction of relationship with colorectal cancer

Appendix Table 3: Sequelae for colorectal cancer and associated disability weights from GBD 2017

Appendix Table 4: Deaths of colorectal cancer in 1990 and 2017 for both sexes and percentage change of age-standardised rates by 195 countries and territories

Appendix Table 5: DALYs of colorectal cancer in 1990 and 2017 for both sexes and percentage change of age-standardised rates by 195 countries and territories

Appendix Figure 1: Number of incident cases of colorectal cancer from 1990 to 2017 for 21 Global Burden of Disease regions

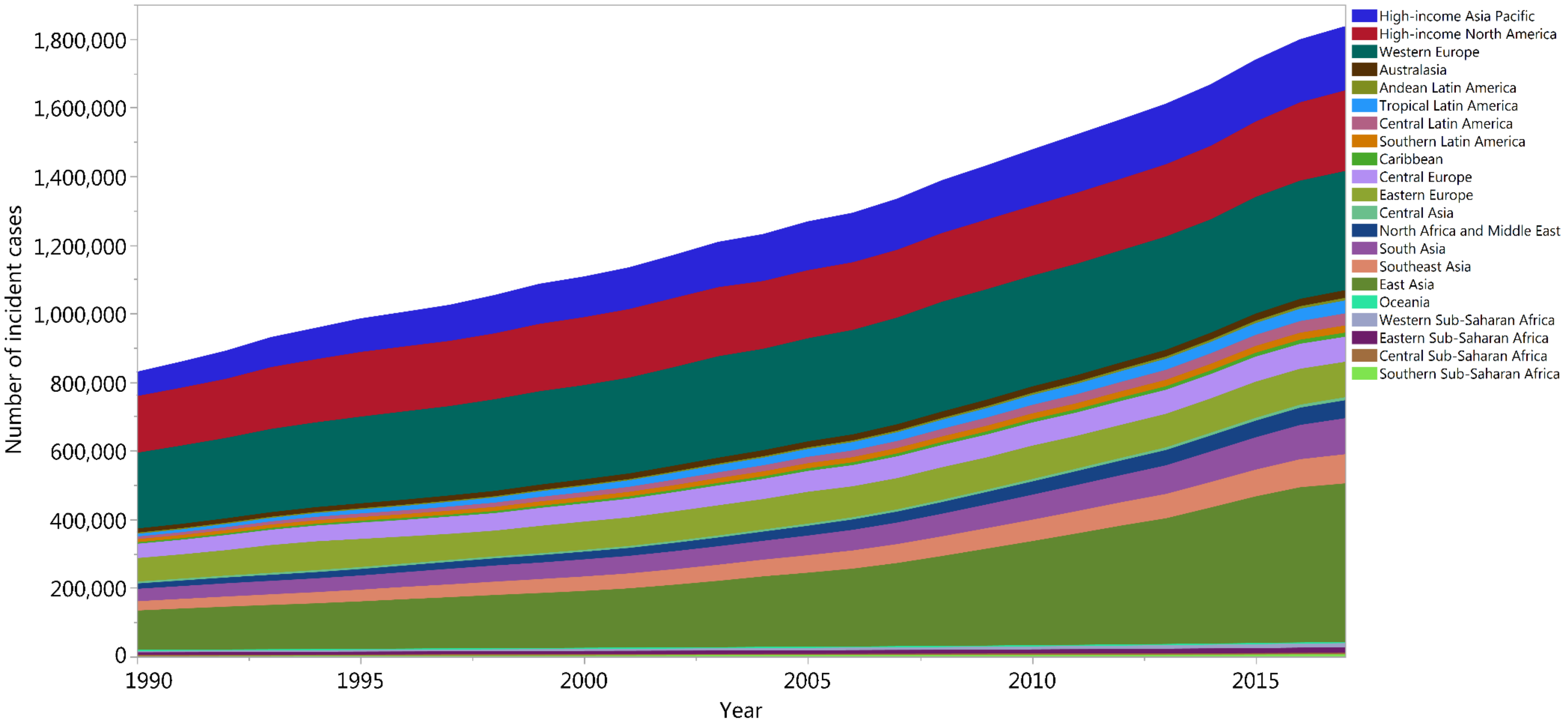

Appendix Figure 2: Number of deaths of colorectal cancer from 1990 to 2017 for 21 Global Burden of Disease regions

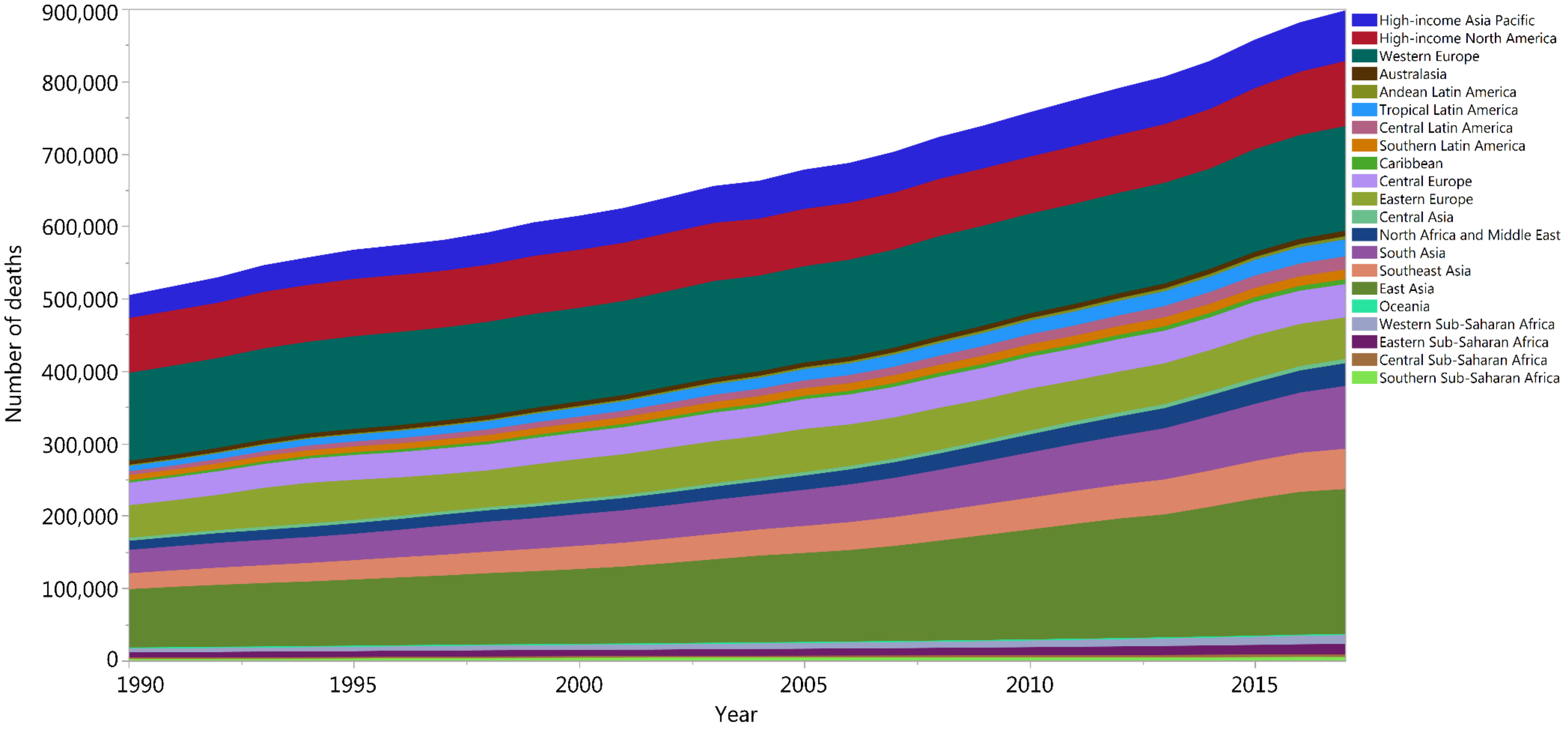

Appendix Figure 3: Global number of deaths and death rate of colorectal cancer per 100 000 population by age and sex, 2017

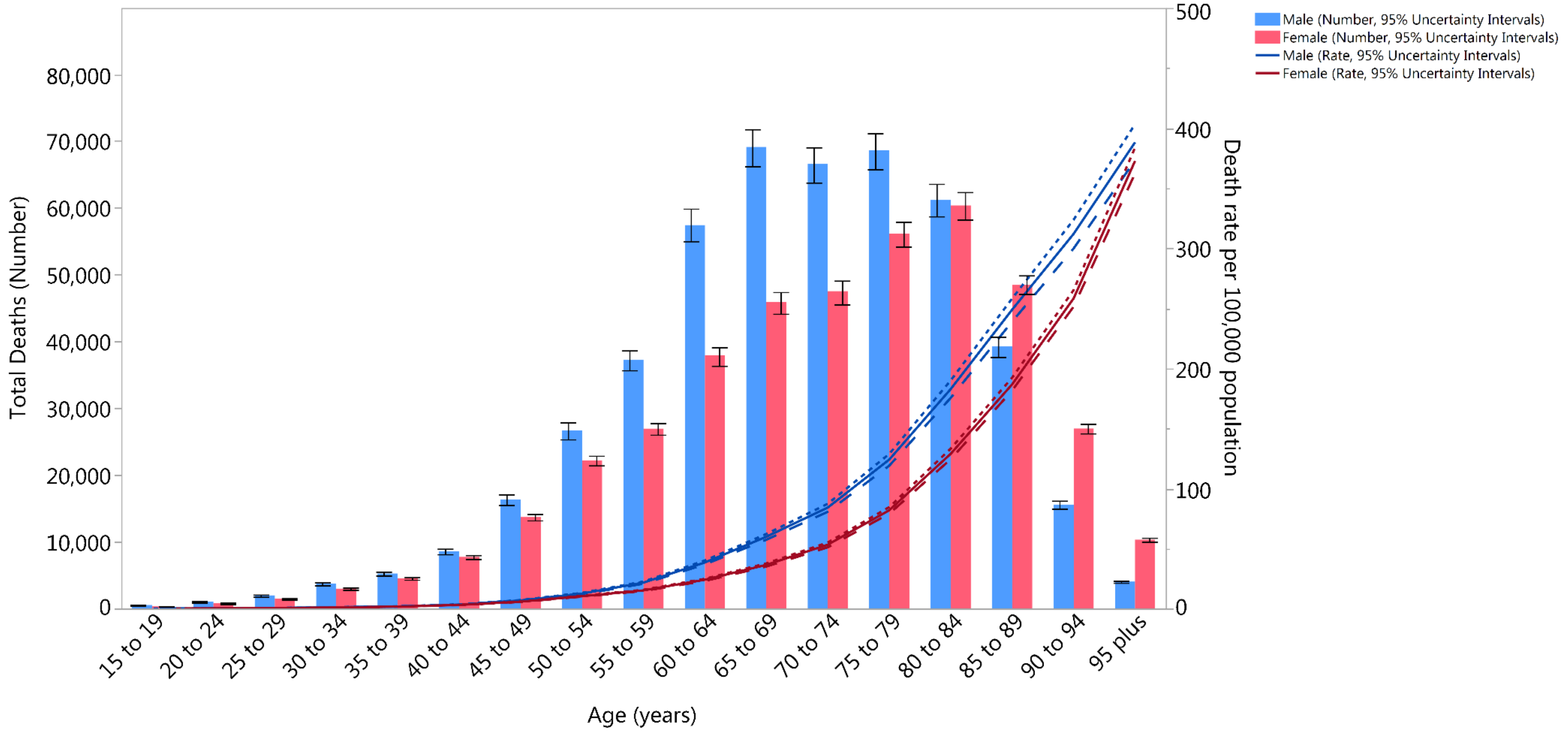

Dotted and dashed lines indicate 95% upper and lower uncertainty intervals, respectively.

Appendix Figure 4: Global number of DALYs and DALY rate of colorectal cancer per 100, 000 population by age and sex, 2017

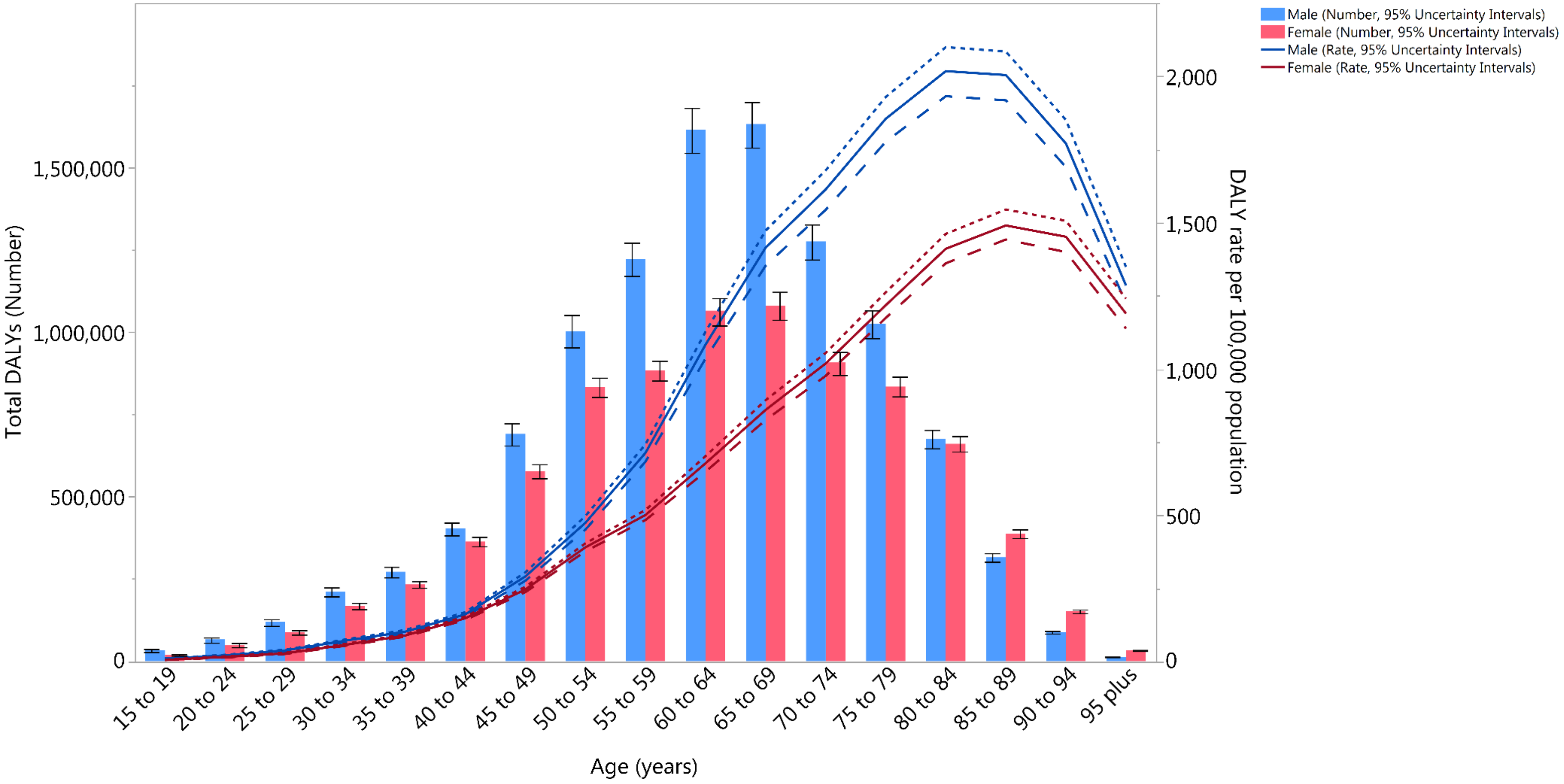

Dotted and dashed lines indicate 95% upper and lower uncertainty intervals, respectively.

Appendix Figure 5: Global number of YLLs and YLDs and rate of YLLs and YLDs per 100 000 population due to colorectal cancer by age, 2017

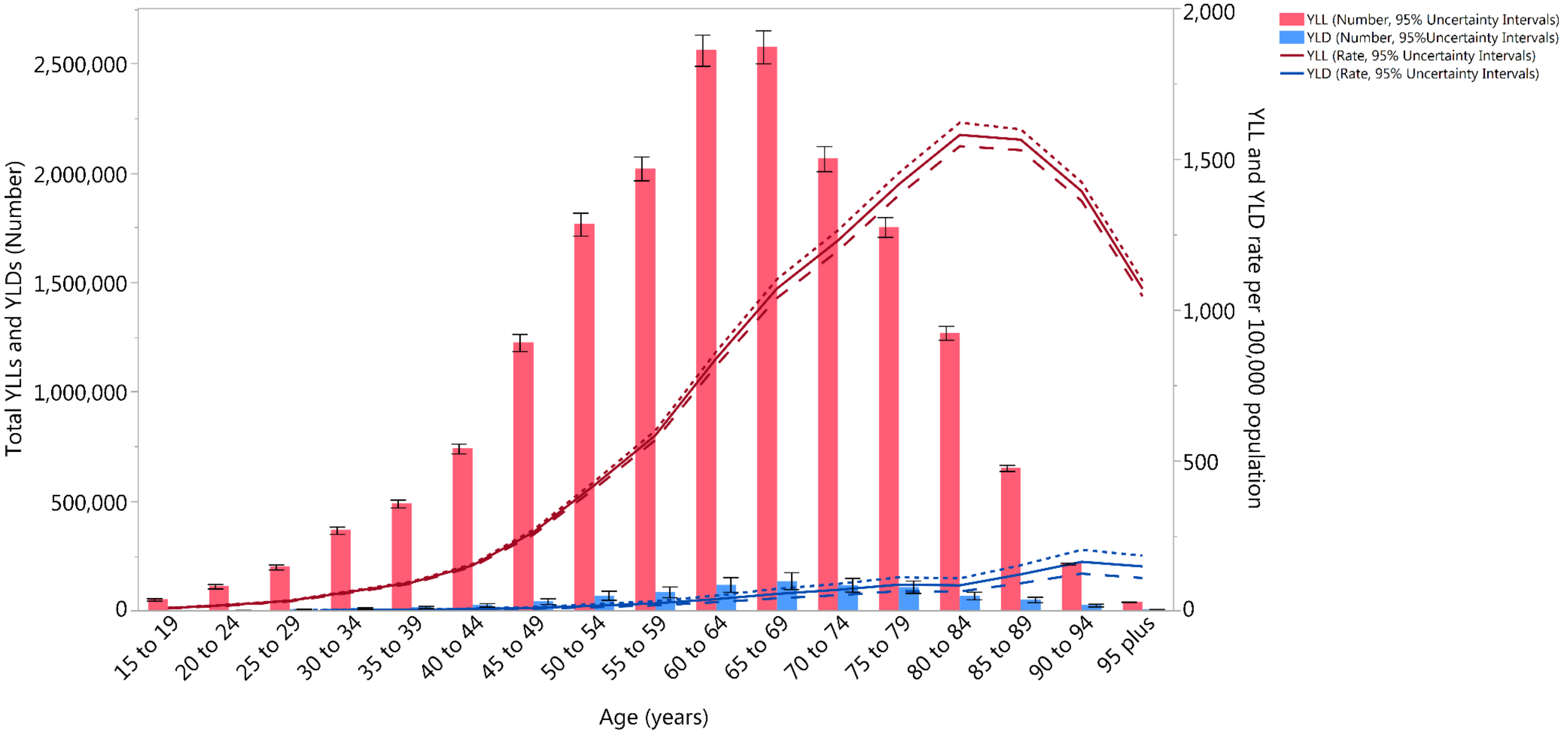

Dotted and dashed lines indicate 95% upper and lower uncertainty intervals, respectively. YLDs=years lived with disability. YLLs=years of life lost.

Appendix Figure 6: Percentage of DALYs due to colorectal cancer attributable to risk factors for 21 Global Burden of Disease regions, 2017, males.

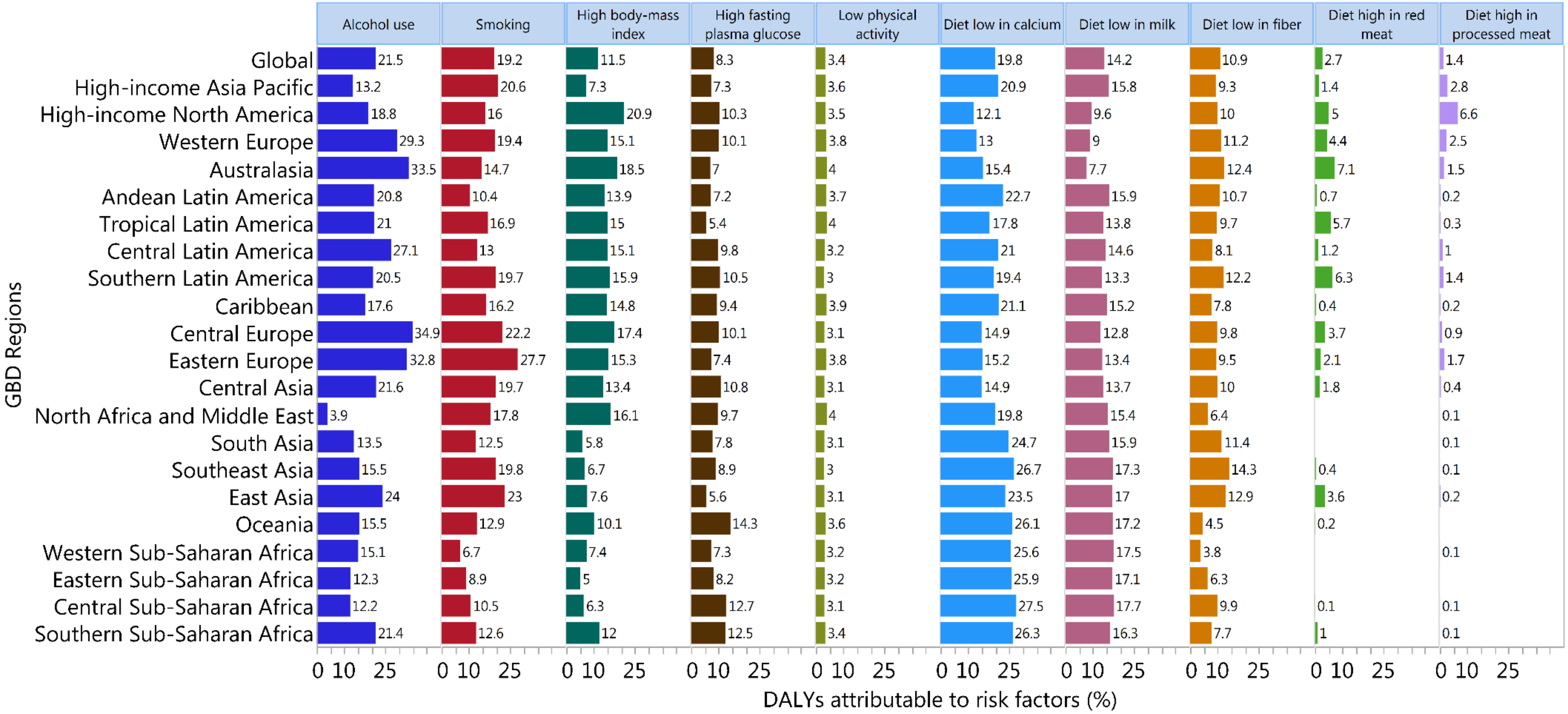

DALY=disability-adjusted life-year.

Appendix Figure 7: Percentage of DALYs due to colorectal cancer attributable to risk factors for 21 Global Burden of Disease regions, 2017, females.

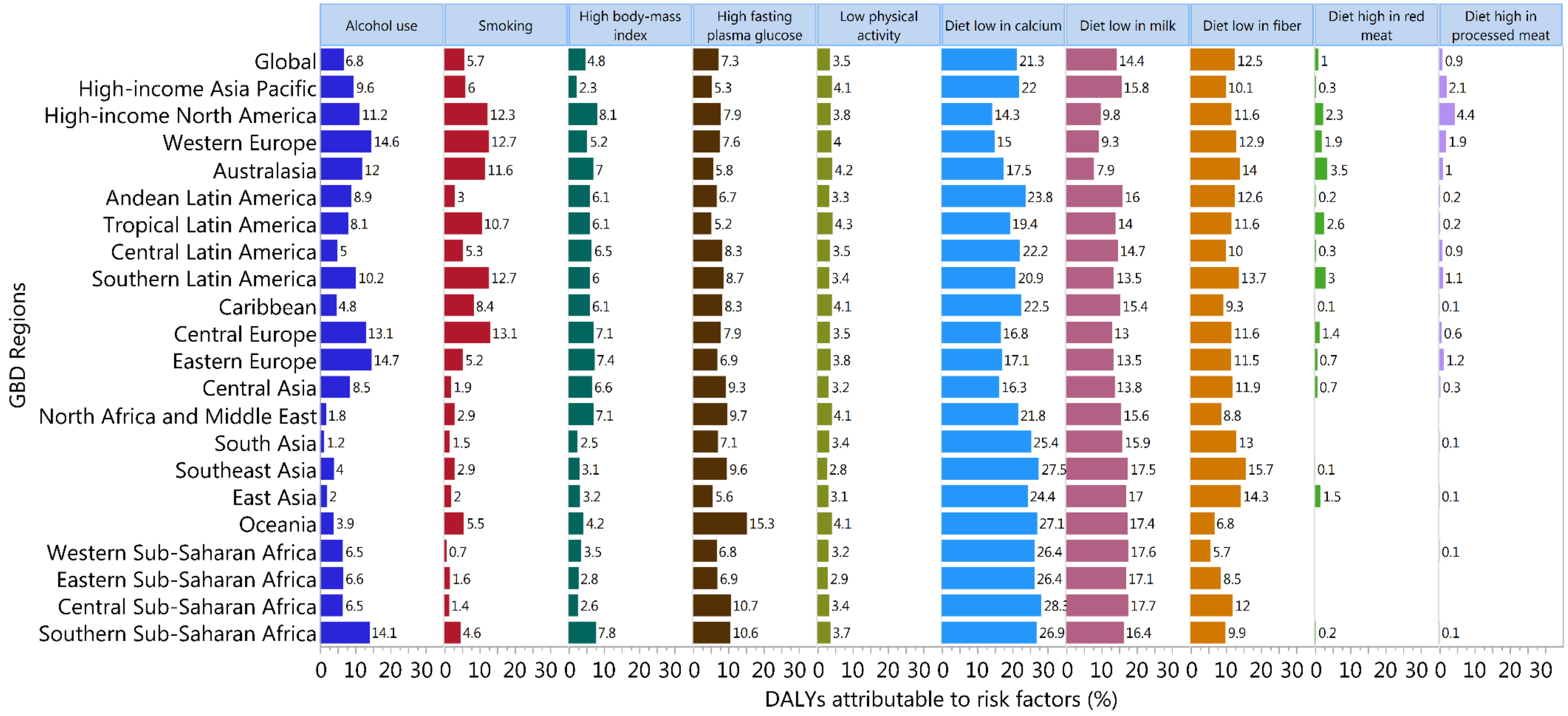

DALY=disability-adjusted life-year.

Appendix Figure 8: Percentage of DALYs due to colorectal cancer attributable to risk factors by age for both sexes, 2017

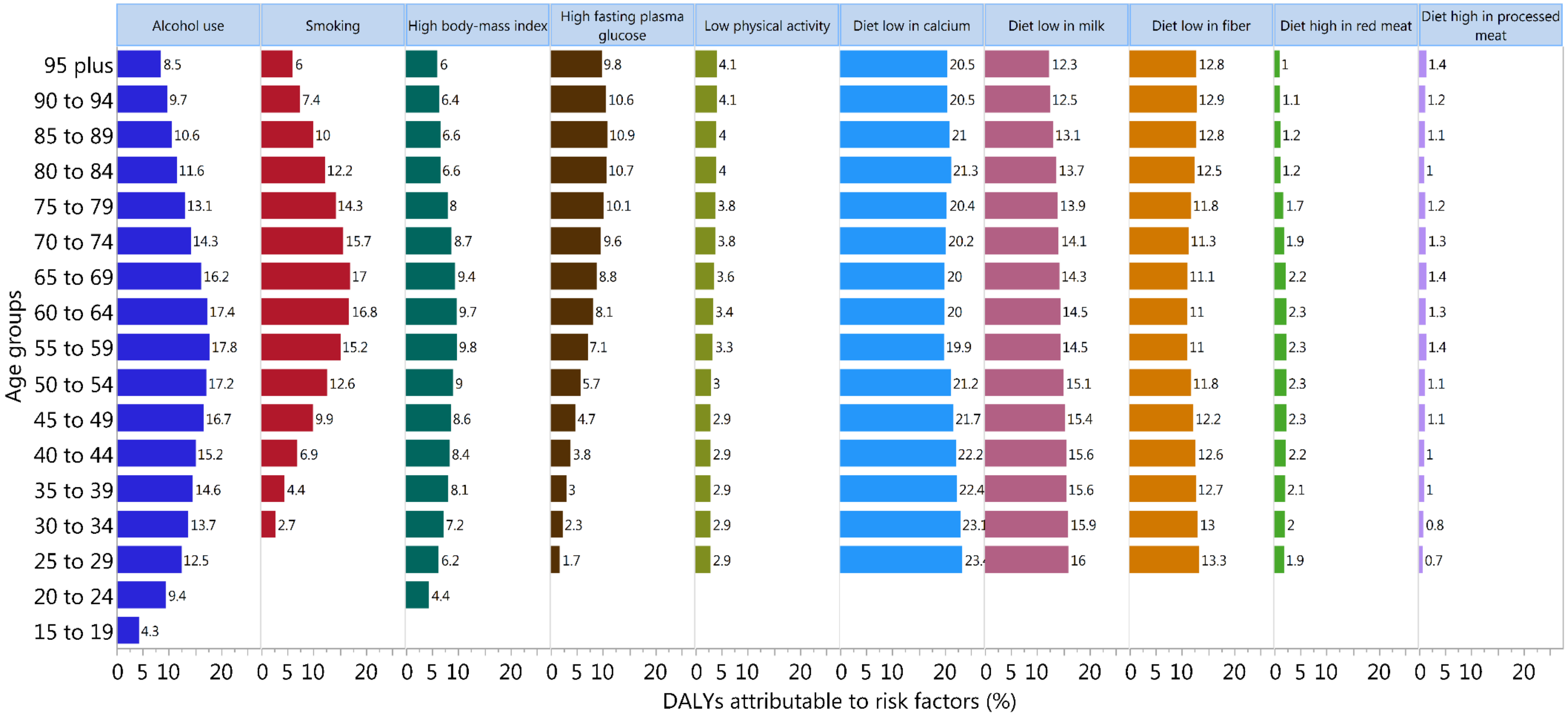

DALY=disability-adjusted life-year.

Appendix Figure 9: Percentage of DALYs due to colorectal cancer attributable to risk factors by age for males, 2017

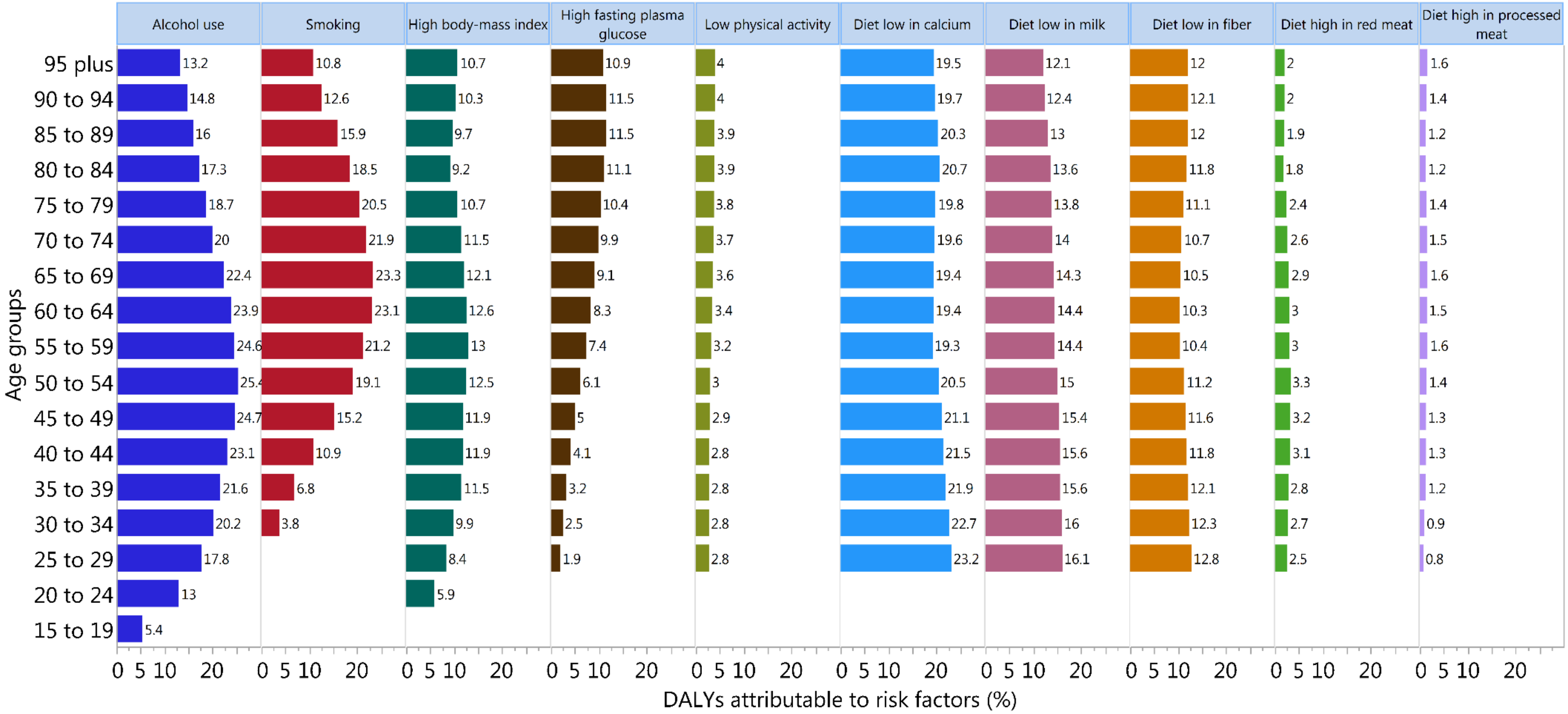

DALY=disability-adjusted life-year.

Appendix Figure 10: Percentage of DALYs due to colorectal cancer attributable to risk factors by age for females, 2017

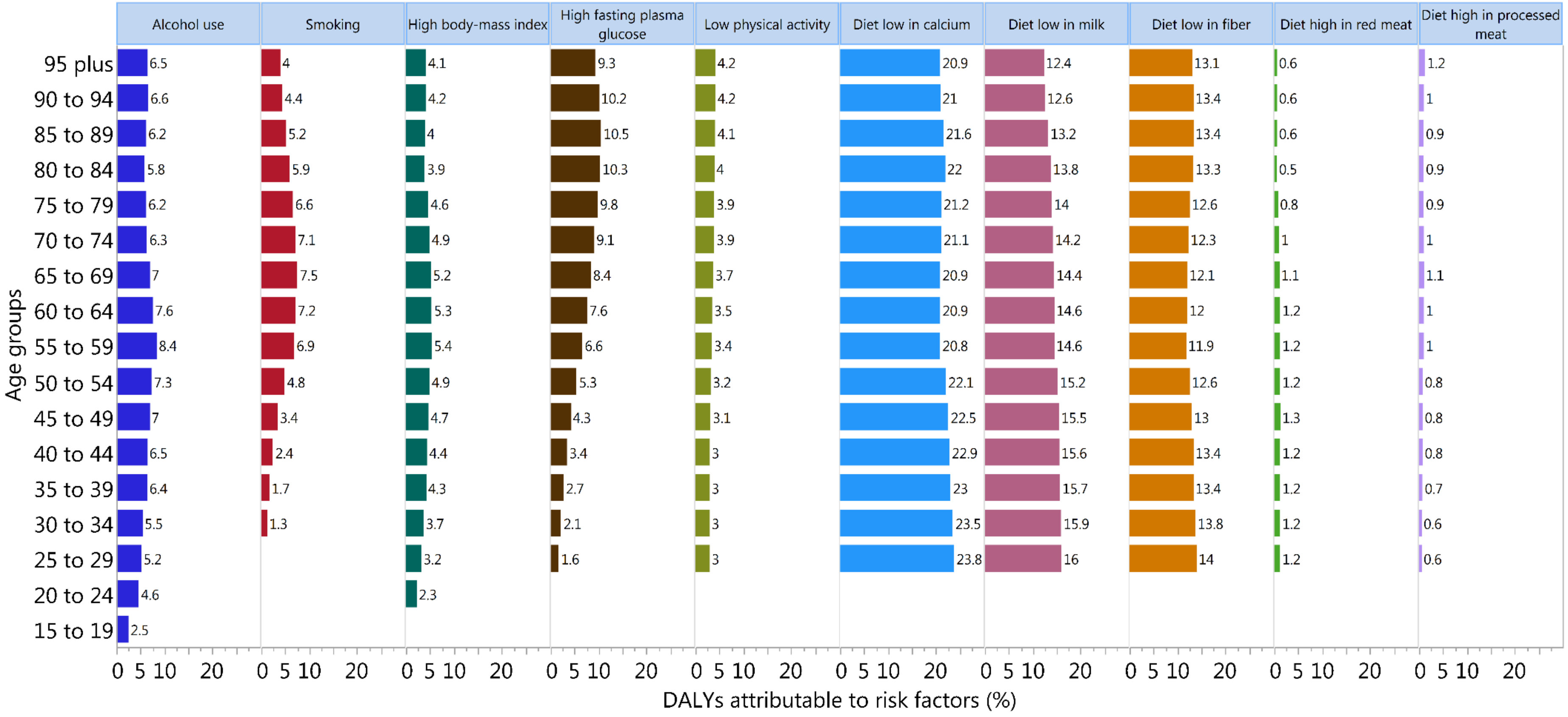

DALY=disability-adjusted life-year.

Appendix Figure 11: Percentage and UIs of DALYs due to colorectal cancer attributable to risk factors for 21 GBD regions, both sexes, 2017.

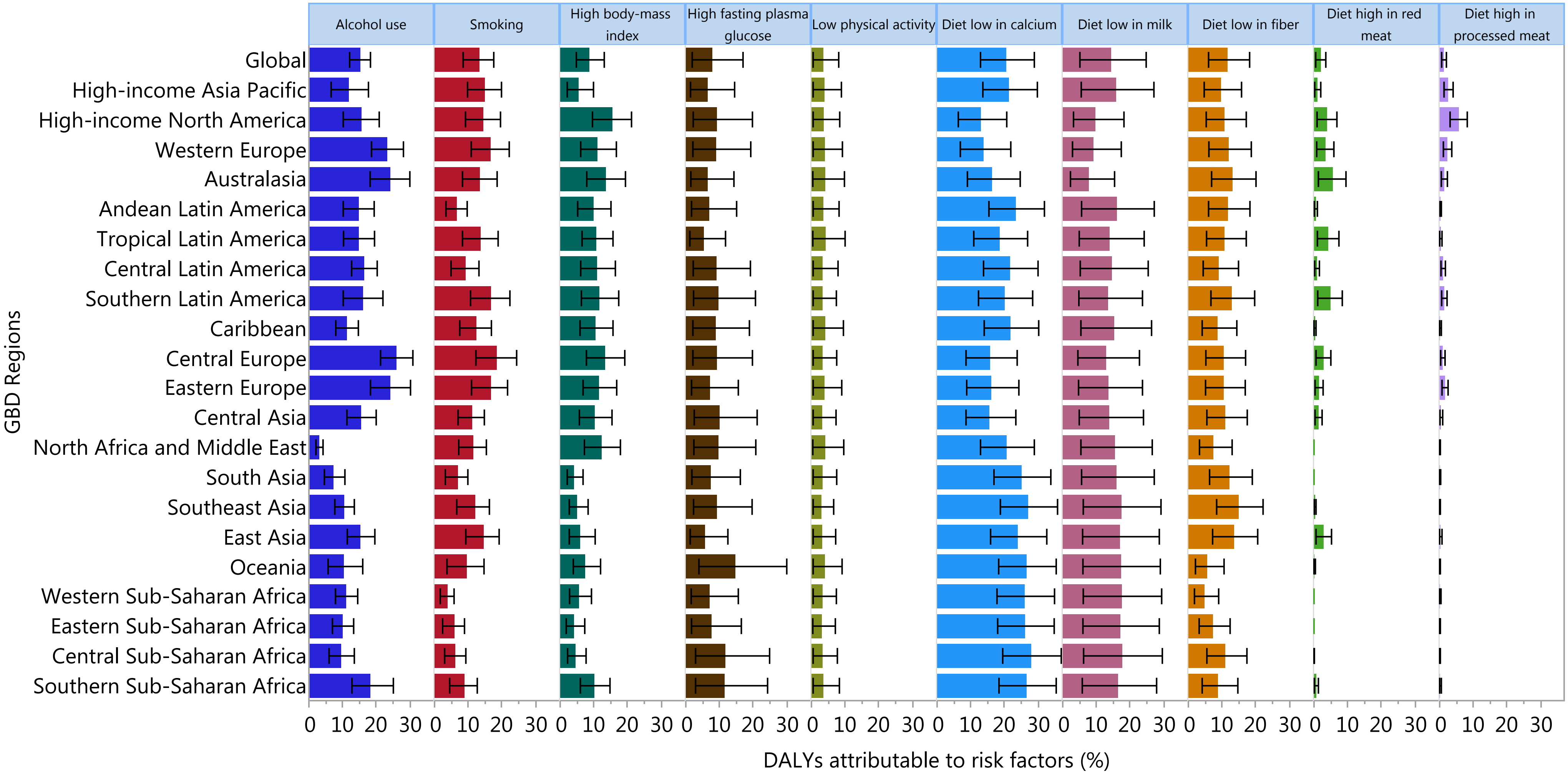

A supplement to figure 7 in the manuscript, which does not include UIs. DALY=disability-adjusted life-year. UI=uncertainty interval. GBD=Global Burden of Disease.

Appendix Table 1. Data quality rating from 0 to 5 stars, maximum percent well-certified per 5-year interval and percent well-certified across time series for 195 countries, 1980–2017. [1]

Country	Data Quality Rating	1980-1984	1985-1989	1990-1994	1995-1999	2000-2004	2005-2009	2010-2017	1980-2017
Afghanistan	1	0.0	0.0	0.0	0.0	4.6	38.2	0.0	6.1
Albania	3	0.0	66.1	68.6	70.7	71.8	72.5	54.7	57.8
Algeria	1	0.0	0.0	0.0	0.0	0.0	17.3	0.0	2.5
American Samoa	3	0.0	0.0	0.0	73.7	78.2	83.4	79.4	45.0
Andorra	0	0.0	0.0	0.0	0.0	0.0	0.0	0.0	0.0
Angola	1	0.0	0.0	0.0	0.0	0.0	0.0	4.4	0.6
Antigua and Barbuda	4	60.9	72.0	75.1	79.5	79.3	80.0	74.7	74.5
Argentina	4	77.4	70.9	69.7	68.5	66.7	66.6	69.9	70.0
Armenia	5	74.2	78.1	85.0	88.8	88.9	92.7	91.9	85.6
Australia	5	93.2	93.6	92.9	92.5	91.1	90.5	90.0	91.9
Austria	5	89.9	90.9	90.0	88.6	91.9	90.8	89.2	90.2
Azerbaijan	3	75.5	75.4	78.1	72.0	68.2	41.8	0.0	58.7
Bahrain	3	0.0	78.1	0.0	61.6	64.6	55.9	56.8	45.3
Bangladesh	1	2.8	4.5	25.7	4.4	12.4	6.3	6.3	8.9
Barbados	4	75.0	75.8	77.6	77.4	75.6	82.1	82.0	77.9
Belarus	4	84.0	88.9	80.1	82.9	85.4	84.5	85.0	84.4
Belgium	4	77.8	77.6	81.3	84.1	82.8	82.7	80.4	81.0
Belize	4	47.5	52.4	49.8	76.6	78.3	87.1	89.1	68.7
Benin	1	0.0	0.6	0.0	0.0	0.0	0.0	0.0	0.1
Bermuda	5	90.4	88.7	90.2	93.5	91.1	86.4	90.3	90.1
Bhutan	0	0.0	0.0	0.0	0.0	0.0	0.0	0.0	0.0
Bolivia	1	0.0	0.0	0.0	0.0	14.7	0.0	0.0	2.1
Bosnia and Herzegovina	2	0.0	65.8	67.1	0.0	0.0	0.0	71.4	29.2
Botswana	0	0.0	0.0	0.0	0.0	0.0	0.0	0.0	0.0
Brazil	4	54.0	58.9	65.2	68.9	74.6	80.7	82.5	69.3
Brunei	3	0.0	0.0	0.0	66.7	73.2	76.1	74.5	41.5

Bulgaria	4	81.7	82.3	79.6	76.5	71.8	73.5	70.2	76.5
Burkina Faso	1	0.2	0.0	0.0	4.6	6.3	4.6	0.3	2.3
Burundi	1	0.0	0.0	2.2	0.0	0.0	0.0	0.0	0.3
Cambodia	1	0.0	0.0	0.0	0.0	1.6	4.4	0.0	0.9
Cameroon	0	0.0	0.0	0.0	0.0	0.0	0.0	0.0	0.0
Canada	5	89.4	90.0	89.1	88.6	89.3	89.4	90.1	89.4
Cape Verde	2	56.1	0.0	0.1	0.0	0.0	0.0	67.7	17.7
Central African Republic	0	0.0	0.0	0.0	0.0	0.0	0.0	0.0	0.0
Chad	0	0.0	0.0	0.0	0.0	0.0	0.0	0.0	0.0
Chile	4	75.7	75.5	81.7	84.7	90.8	90.8	89.8	84.1
China	3	0.0	0.9	73.8	75.1	70.3	73.0	73.5	52.4
Colombia	4	72.4	74.4	77.3	88.0	88.6	88.9	88.5	82.6
Comoros	0	0.0	0.0	0.0	0.0	0.0	0.0	0.0	0.0
Congo	0	0.0	0.0	0.0	0.0	0.0	0.0	0.0	0.0
Costa Rica	5	81.6	83.4	82.5	91.1	92.3	90.6	90.5	87.4
Côte d’Ivoire	1	0.0	1.2	1.2	0.0	0.0	0.2	0.2	0.4
Croatia	4	0.0	84.4	85.3	82.6	86.1	88.3	92.3	74.1
Cuba	5	84.7	85.1	84.9	88.9	90.2	91.0	91.5	88.0
Cyprus	2	0.0	0.0	0.0	26.8	53.8	59.5	60.9	28.7
Czech Republic	4	0.0	90.7	89.6	84.8	84.7	85.8	87.8	74.8
DR Congo	1	0.0	2.3	3.0	0.0	0.0	0.0	0.0	0.8
Denmark	4	84.5	82.9	83.1	85.8	85.4	84.5	84.6	84.4
Djibouti	0	0.0	0.0	0.0	0.0	0.0	0.0	0.0	0.0
Dominica	4	71.6	63.1	62.1	65.8	68.6	81.7	83.9	71.0
Dominican Republic	3	53.6	53.5	44.5	53.8	57.2	56.8	59.9	54.2
Ecuador	4	73.0	72.4	72.9	69.8	66.4	62.9	70.4	69.7
Egypt	3	35.0	48.2	46.8	0.0	43.9	42.7	48.2	37.8
El Salvador	3	73.3	0.0	60.4	67.2	69.4	70.8	67.4	58.4
Equatorial Guinea	0	0.0	0.0	0.0	0.0	0.0	0.0	0.0	0.0
Eritrea	0	0.0	0.0	0.0	0.0	0.0	0.0	0.0	0.0

Estonia	5	92.1	92.7	93.8	93.1	92.5	94.1	93.9	93.2
Ethiopia	1	0.0	1.2	2.5	0.6	5.0	4.6	4.6	2.6
Federated States of Micronesia	0	0.0	0.0	0.0	0.0	0.0	0.0	0.0	0.0
Fiji	2	0.0	0.0	0.0	34.8	58.0	63.9	70.0	32.4
Finland	5	83.7	91.7	91.7	95.8	95.6	94.9	95.9	92.8
France	4	76.1	78.4	79.1	79.0	79.1	79.4	77.4	78.3
Gabon	0	0.0	0.0	0.0	0.0	0.0	0.0	0.0	0.0
Georgia	4	83.4	80.7	81.3	77.7	78.7	62.2	58.9	74.7
Germany	4	78.4	80.5	84.1	83.9	83.5	83.8	84.0	82.6
Ghana	1	0.0	0.1	1.5	0.8	7.2	16.7	0.5	3.8
Greece	4	80.2	81.6	71.7	72.0	72.6	77.9	76.2	76.0
Greenland	3	0.0	0.0	0.0	90.2	89.7	89.6	87.6	51.0
Grenada	4	67.7	63.4	64.0	63.2	77.0	79.9	85.9	71.6
Guam	3	0.0	0.0	85.9	80.7	76.7	79.9	79.2	57.5
Guatemala	4	80.1	75.0	73.9	72.6	73.3	75.2	78.0	75.4
Guinea	1	0.0	0.0	0.0	3.2	0.0	0.0	0.0	0.5
Guinea-Bissau	1	0.0	0.0	0.1	1.2	0.0	0.0	0.0	0.2
Guyana	4	55.5	76.2	69.8	78.3	84.3	85.5	78.6	75.5
Haiti	1	22.3	1.3	1.0	13.9	6.1	0.0	0.0	6.4
Honduras	2	41.2	46.0	43.2	0.0	0.0	17.5	19.1	23.8
Hungary	5	91.0	89.8	89.9	91.0	92.1	92.6	93.8	91.5
Iceland	5	92.6	93.8	94.2	94.1	93.5	92.9	91.4	93.2
India	2	2.8	3.0	2.9	5.2	6.3	52.7	49.0	17.4
Indonesia	2	1.4	0.0	1.3	0.4	0.3	53.7	62.3	17.1
Iran	2	0.0	0.0	0.0	0.0	52.8	63.7	80.3	28.1
Iraq	2	0.0	0.0	0.0	0.0	0.0	45.8	56.7	14.6
Ireland	5	90.3	91.6	91.7	90.9	90.2	92.4	92.3	91.3
Israel	4	81.2	82.6	83.2	82.7	81.8	80.1	80.7	81.8
Italy	5	88.8	88.4	88.5	87.8	88.1	88.7	88.3	88.4
Jamaica	4	70.5	72.8	64.6	0.0	83.0	87.5	89.1	66.8

Japan	4	82.6	81.0	81.5	87.6	85.7	84.4	81.3	83.4
Jordan	2	0.0	0.0	0.0	1.9	70.1	76.5	74.1	31.8
Kazakhstan	5	80.0	84.9	89.1	88.6	85.0	84.2	84.1	85.1
Kenya	1	0.0	2.7	0.0	0.4	4.9	5.4	0.8	2.0
Kiribati	2	0.0	0.0	47.9	66.6	35.6	0.0	0.0	21.4
Kuwait	4	82.1	81.9	76.4	77.5	83.1	84.9	84.6	81.5
Kyrgyzstan	4	67.5	75.9	72.5	74.4	87.4	92.9	93.2	80.6
Laos	1	0.0	1.3	0.0	0.0	0.0	0.0	0.0	0.2
Latvia	5	92.5	92.7	89.3	92.0	91.1	88.7	94.3	91.5
Lebanon	1	0.0	3.5	0.0	0.0	0.0	0.0	0.0	0.5
Lesotho	0	0.0	0.0	0.0	0.0	0.0	0.0	0.0	0.0
Liberia	1	2.1	2.1	3.2	0.0	0.0	0.0	0.0	1.1
Libya	1	0.0	0.0	0.0	0.0	0.0	12.6	0.0	1.8
Lithuania	5	90.1	94.0	93.7	94.8	92.9	93.3	94.6	93.3
Luxembourg	4	86.4	85.8	85.5	84.5	80.6	77.9	82.5	83.3
Macedonia	3	0.0	0.0	82.5	83.8	84.5	81.7	79.6	58.9
Madagascar	1	2.8	3.5	2.4	2.4	0.0	0.0	0.0	1.6
Malawi	1	0.0	2.5	0.0	0.6	2.5	4.5	0.6	1.5
Malaysia	2	46.9	0.0	0.0	32.1	37.3	42.0	40.2	28.4
Maldives	2	0.0	0.0	0.0	0.0	44.0	46.6	60.1	21.5
Mali	1	4.0	0.0	0.1	0.0	0.0	0.0	0.0	0.6
Malta	5	81.2	86.3	88.7	90.0	90.3	92.8	89.5	88.4
Marshall Islands	0	0.0	0.0	0.0	0.0	0.0	0.0	0.0	0.0
Mauritania	0	0.0	0.0	0.0	0.0	0.0	0.0	0.0	0.0
Mauritius	4	75.2	79.4	78.7	78.2	83.2	85.1	86.3	80.9
Mexico	4	68.3	77.7	78.5	79.7	82.4	84.1	88.1	79.8
Moldova	5	88.8	91.2	81.9	87.8	85.0	83.5	83.7	86.0
Mongolia	2	0.0	0.0	61.2	0.0	19.6	21.4	83.4	26.5
Montenegro	2	0.0	0.0	0.0	0.0	69.2	69.2	0.0	19.8
Morocco	2	0.0	15.4	0.0	0.0	10.2	33.3	13.7	10.4

Mozambique	2	0.0	0.0	0.0	0.1	16.2	63.4	0.0	11.4
Myanmar	1	0.0	0.0	0.0	0.0	0.0	2.9	49.5	7.5
Namibia	0	0.0	0.0	0.0	0.0	0.0	0.0	0.0	0.0
Nepal	1	2.8	2.6	0.0	0.6	0.6	9.2	0.0	2.3
Netherlands	4	88.5	86.7	85.2	83.9	82.3	83.2	83.3	84.7
New Zealand	5	95.3	95.2	95.1	96.8	96.4	96.3	95.7	95.8
Nicaragua	3	0.0	54.3	56.9	69.0	77.2	84.9	90.5	61.8
Niger	1	0.0	0.0	0.0	0.0	0.0	35.7	0.0	5.1
Nigeria	1	0.0	0.0	3.3	0.0	0.0	0.1	32.3	5.1
North Korea	0	0.0	0.0	0.0	0.0	0.0	0.0	0.0	0.0
Northern Mariana Islands	3	0.0	0.0	0.0	75.3	71.4	80.3	82.5	44.2
Norway	5	88.6	89.4	88.6	88.5	86.3	84.2	84.1	87.1
Oman	2	0.0	0.0	0.0	0.0	0.0	73.2	34.4	15.4
Pakistan	1	0.0	2.8	1.6	0.0	1.5	17.8	1.9	3.7
Palestine	2	0.0	0.0	0.0	29.7	32.2	34.2	74.1	24.3
Panama	4	72.3	75.3	0.0	83.5	86.6	86.3	84.0	69.7
Papua New Guinea	1	9.4	3.4	0.0	0.0	0.0	0.0	28.8	6.0
Paraguay	3	51.3	51.2	57.2	62.7	63.6	68.8	74.0	61.3
Peru	3	54.9	36.1	37.2	51.1	63.3	65.4	65.9	53.4
Philippines	4	68.2	71.1	65.9	68.6	74.3	74.1	74.8	71.0
Poland	4	61.8	59.5	59.9	71.6	73.7	73.5	72.6	67.5
Portugal	4	76.9	77.1	76.3	76.2	79.1	77.7	80.1	77.6
Puerto Rico	4	78.6	76.7	83.9	83.2	83.6	83.6	84.4	82.0
Qatar	3	14.7	18.9	0.0	55.1	56.3	64.9	46.1	36.6
Romania	4	76.0	78.0	83.3	84.8	85.9	86.2	85.4	82.8
Russian Federation	5	93.0	87.3	90.7	87.6	88.0	89.3	88.8	89.3
Rwanda	1	0.0	0.0	0.0	0.0	0.0	22.6	0.0	3.2
Saint Lucia	4	66.8	68.2	73.2	72.1	80.0	79.0	85.6	75.0
Saint Vincent and the Grenadines	4	72.3	63.4	61.2	85.3	85.1	87.6	87.3	77.5
Samoa	0	0.0	0.0	0.0	0.0	0.0	0.0	0.0	0.0

São Tomé and Príncipe	1	0.0	69.6	0.0	0.0	0.0	0.0	0.0	9.9
Saudi Arabia	2	0.0	0.0	0.0	23.1	27.5	29.4	28.8	15.5
Senegal	1	2.0	2.5	2.6	2.5	0.0	0.0	0.0	1.4
Serbia	3	0.0	0.0	0.0	66.7	70.8	74.9	69.6	40.3
Seychelles	3	74.4	71.5	0.0	0.0	75.9	76.9	78.1	53.8
Sierra Leone	1	0.0	0.0	3.4	0.0	0.0	0.0	0.0	0.5
Singapore	5	91.2	92.0	94.6	95.1	95.3	94.9	95.7	94.1
Slovakia	3	0.0	0.0	82.7	81.9	84.9	90.2	92.9	61.8
Slovenia	4	0.0	91.6	92.5	91.8	89.3	88.3	88.5	77.4
Solomon Islands	1	0.0	0.0	0.0	0.0	0.0	0.0	34.6	4.9
Somalia	0	0.0	0.0	0.0	0.0	0.0	0.0	0.0	0.0
South Africa	3	0.0	0.0	1.0	67.8	72.0	72.3	70.7	40.5
South Korea	4	0.0	57.8	74.2	75.4	84.5	82.2	81.9	65.1
South Sudan	0	0.0	0.0	0.0	0.0	0.0	0.0	0.0	0.0
Spain	4	77.3	79.4	82.6	83.8	83.1	84.0	84.8	82.1
Sri Lanka	3	52.0	50.2	47.7	55.4	63.7	67.4	64.8	57.3
Sudan	0	0.0	0.0	0.0	0.0	0.0	0.0	0.0	0.0
Suriname	4	60.0	63.7	61.2	66.4	74.3	73.3	73.9	67.5
Swaziland	1	0.0	0.0	0.0	0.0	0.0	0.0	0.0	0.0
Sweden	5	87.4	88.6	88.2	87.1	85.7	85.9	84.8	86.8
Switzerland	4	73.8	74.2	73.4	84.6	84.4	86.7	86.1	80.5
Syria	3	29.5	13.2	0.0	54.3	66.7	74.4	59.8	42.5
Taiwan (Province of China)	4	84.3	82.4	79.2	83.4	84.4	84.8	83.7	83.2
Tajikistan	3	74.9	71.4	71.6	58.5	51.4	50.6	50.7	61.3
Tanzania	1	0.0	2.8	1.8	2.1	6.3	2.5	0.0	2.2
Thailand	3	29.9	28.3	35.9	62.6	52.0	61.6	62.3	47.5
The Bahamas	4	76.2	82.6	83.0	89.8	87.7	86.7	86.3	84.6
The Gambia	1	2.8	2.2	2.1	0.9	0.7	1.0	0.0	1.4
Timor-Leste	0	0.0	0.0	0.0	0.0	0.0	0.0	0.0	0.0
Togo	0	0.0	0.0	0.0	0.0	0.0	0.0	0.0	0.0

Tonga	1	0.0	0.0	0.0	0.0	53.6	0.0	0.0	7.7
Trinidad and Tobago	5	79.2	83.4	85.5	89.5	90.7	89.6	90.1	86.9
Tunisia	1	0.0	0.0	0.0	0.0	0.0	28.9	24.8	7.7
Turkey	3	16.8	19.6	20.7	24.0	59.7	70.3	83.9	42.2
Turkmenistan	4	88.4	88.1	83.0	80.9	85.1	81.0	83.4	84.3
Uganda	1	0.0	0.0	0.0	0.0	0.0	2.7	0.0	0.4
Ukraine	5	87.4	90.0	83.5	86.0	86.4	90.9	92.2	88.1
United Arab Emirates	1	0.0	0.0	0.0	0.0	0.0	41.5	0.0	5.9
United Kingdom	5	94.2	94.1	94.1	92.0	91.4	91.5	92.4	92.8
USA	5	88.5	88.1	88.7	88.1	88.0	87.2	86.7	87.9
Uruguay	4	77.3	76.0	79.1	79.1	79.6	78.5	77.5	78.1
Uzbekistan	4	83.1	86.3	85.0	79.0	68.3	72.2	79.8	79.1
Vanuatu	0	0.0	0.0	0.0	0.0	0.0	0.0	0.0	0.0
Venezuela	5	81.3	75.2	83.3	87.8	89.8	89.5	88.9	85.1
Vietnam	1	0.0	0.7	0.2	1.3	0.9	62.0	4.8	10.0
Virgin Islands	4	82.0	0.0	90.6	90.5	84.1	81.9	74.0	71.8
Yemen	0	0.0	0.0	0.0	0.0	0.0	0.0	0.0	0.0
Zambia	1	0.0	0.0	0.0	0.0	0.0	6.3	9.5	2.3
Zimbabwe	2	0.0	0.0	45.3	74.2	0.0	45.3	0.0	23.5

The value of 0 shows lowest quality and 5 denotes highest quality. The percent of well-certified sources were the proportion of cause of death data that didn’t have garbage codes at levels 1 and 2.

Appendix Table 2. Covariates used in Cause of Death Ensemble model and assumed direction of relationship with colorectal cancer

Level	Covariate	Direction
1	Alcohol (litres per capita)	+
	Mean BMI	+
	Smoking prevalence	+
	Tobacco (cigarettes per capita)	+
	Log-transformed summary exposure value (SEV) scalar: Colorect C	+
	Red meats adjusted (g)	+
2	Milk adjusted (g)	-
	Fruits adjusted (g)	-
	Nuts seeds adjusted (g)	-
	whole grains adjusted (g)	-
	Polyunsaturated fatty acid adjusted (percent)	-
	Vegetables adjusted (g)	-
	Cumulative cigarettes (5 years)	+
	Cumulative cigarettes (10 years)	+
	Cumulative cigarettes (15 years)	+
	Cumulative cigarettes (20 years)	+
	Diabetes age-specific prevalence (proportion)	+
	Healthcare Access and Quality Index	-
3	Education (years per capita)	-
	Lag distributed income (I$ per capita)	0
	Socio-demographic Index	0

The SEV scalar reflects the exposure to risk factors related to CRC weighted by their relative risk value, methods for the SEV scalar have been described previously. [2]

Appendix Table 3: Sequelae for colorectal cancer and associated disability weights from GBD 2017

Sequela	Health state name	Lay description	Disability weight (95% CI)
Diagnosis and primary therapy phase	Cancer, diagnosis and primary therapy	Has pain, nausea, fatigue, weight loss, and high anxiety.	0.288 (0.193-0.399)
Controlled phase, with stoma	Stoma and generic medication	Combined disability weight.	--
Controlled phase, without stoma	Generic uncomplicated disease: worry and daily medication	Has a chronic disease that requires medication every day and causes some worry but minimal interference with daily activities.	0.049 (0.031-0.072)
Metastatic phase	Cancer, metastatic	Has severe pain, extreme fatigue, weight loss, and high anxiety.	0.451 (0.307-0.600)
Terminal phase	Terminal phase, with medication (for cancers, end-stage kidney/liver disease)	Has lost a lot of weight and regularly uses strong medication to avoid constant pain. The person has no appetite, feels nauseated, and needs to spend most of the day in bed.	0.540 (0.377-0.687)
Stoma due to colon and rectum cancer, beyond 10 years	Stoma	Has a pouch attached to an opening in the belly to collect and empty stools.	0.095 (0.063 – 0.131)

GBD=Global Burden of Disease

Appendix Table 4: Deaths of colorectal cancer in 1990 and 2017 for both sexes and percentage change of age-standardised rates by 195 countries and territories.

	1990		2017		Percentage change in age-standardised rates between 1990 and 2017
	Counts (95% UI)	Rate (95% UI)	Counts (95% UI)	Rate (95% UI)	
**Global**	502869 (488388, 526175)	13·3 (13, 13·9)	896040 (876279, 915720)	11·5 (11·3, 11·8)	-13·5 (-18·4, -10)
**High-income North America**	75773 (75035, 76693)	20·5 (20·3, 20·8)	89893 (87480, 92289)	14·4 (14, 14·8)	-30 (-32·2, -27·8)
**Canada**	6637 (6497, 6785)	19·9 (19·5, 20·4)	10145 (9454, 10779)	14·5 (13·5, 15·4)	-27·3 (-32·5, -22·4)
**Greenland**	11 (10, 12)	37 (32·9, 41·1)	16 (15, 18)	26·5 (24·2, 28·8)	-28·4 (-38·5, -17·3)
**USA**	69124 (68425, 70000)	20·6 (20·4, 20·9)	79730 (77620, 82035)	14·4 (14, 14·8)	-30·4 (-32·6, -28)
**Australasia**	5500 (5392, 5613)	23 (22·6, 23·5)	7614 (7036, 8224)	15·2 (14, 16·4)	-34 (-39·2, -28·6)
**Australia**	4374 (4274, 4476)	22 (21·5, 22·5)	6059 (5496, 6642)	14·4 (13, 15·8)	-34·6 (-40·9, -28·2)
**New Zealand**	1127 (1093, 1162)	28·2 (27·4, 29·1)	1555 (1457, 1668)	19·7 (18·4, 21·1)	-30·3 (-35·2, -25·4)
**High-income Asia-Pacific**	29995 (29642, 30375)	15·2 (15, 15·3)	68204 (65810, 71047)	14·1 (13·6, 14·7)	-7·1 (-10·4, -3·4)
**Brunei**	20 (17, 23)	20·6 (17·6, 23·7)	62 (57, 68)	21·7 (19·7, 23·9)	5·5 (-11·6, 24·5)
**Japan**	26786 (26471, 27148)	15·9 (15·7, 16·1)	57307 (55396, 59567)	14·7 (14·2, 15·3)	-7·5 (-10·6, -3·9)
**Singapore**	433 (420, 447)	20·3 (19·6, 20·9)	846 (785, 919)	12·7 (11·8, 13·8)	-37·5 (-42·2, -31·8)
**South Korea**	2756 (2666, 2853)	9·8 (9·5, 10·2)	9989 (9142, 10833)	12 (11, 13)	21·8 (10·6, 32·9)
**Western Europe**	121400 (120148, 122748)	19·9 (19·7, 20·1)	144456 (138821, 149986)	14·7 (14·1, 15·3)	-26·1 (-29·1, -23·1)
**Andorra**	10 (8, 12)	18·1 (14·9, 21·8)	21 (17, 24)	14·6 (11·8, 17·3)	-19·7 (-34·2, -3·8)
**Austria**	2718 (2650, 2786)	21·8 (21·3, 22·4)	2384 (2230, 2543)	12·5 (11·7, 13·3)	-42·7 (-46·7, -38·8)
**Belgium**	3617 (3512, 3718)	22·4 (21·8, 23)	3605 (3337, 3885)	14·4 (13·4, 15·6)	-35·5 (-40·4, -30·2)
**Cyprus**	111 (98, 128)	13·1 (11·6, 15·1)	214 (193, 238)	11·1 (10, 12·4)	-15 (-28·9, 2)
**Denmark**	1442 (1404, 1483)	16·9 (16·5, 17·3)	2260 (2100, 2415)	19 (17·7, 20·4)	12·7 (4, 21·7)
**Finland**	988 (962, 1013)	13·5 (13·1, 13·8)	1366 (1275, 1464)	10·8 (10, 11·6)	-20 (-25·8, -13·5)
**France**	18250 (17753, 18775)	20·5 (19·9, 21)	21135 (19529, 22794)	13·8 (12·7, 14·9)	-32·8 (-37·9, -26·8)
**Germany**	32150 (31358, 32904)	23·9 (23·3, 24·4)	30911 (27874, 34210)	15·3 (13·8, 16·9)	-35·8 (-42·4, -28·6)

**Greece**	1529 (1472, 1593)	9·8 (9·5, 10·2)	3150 (2923, 3374)	11·9 (11·1, 12·7)	20·9 (11·8, 30·9)
**Iceland**	42 (39, 44)	13·9 (13·2, 14·6)	64 (60, 69)	11·5 (10·7, 12·3)	-17·3 (-24·1, -10·2)
**Ireland**	984 (953, 1016)	23·6 (22·9, 24·4)	1164 (1061, 1283)	15·7 (14·3, 17·3)	-33·6 (-39·6, -27)
**Israel**	870 (836, 915)	17·9 (17·2, 18·8)	1658 (1539, 1787)	14 (13, 15·1)	-21·6 (-27·2, -15·4)
**Italy**	16127 (15698, 16549)	17·5 (17·1, 17·9)	20982 (19498, 22621)	13·3 (12·3, 14·3)	-24·2 (-29·8, -18·2)
**Luxembourg**	129 (123, 135)	22·8 (21·8, 23·8)	162 (144, 188)	15·9 (14·1, 18·5)	-30 (-38·3, -19·1)
**Malta**	66 (62, 70)	15·6 (14·8, 16·4)	132 (122, 142)	14·4 (13·3, 15·4)	-7·7 (-15·8, 0·2)
**Netherlands**	4448 (4334, 4560)	21·4 (20·9, 21·9)	6580 (6179, 7002)	18·8 (17·6, 20·1)	-12·1 (-17·9, -6·2)
**Norway**	1572 (1550, 1596)	21·9 (21·6, 22·3)	1771 (1694, 1853)	17·9 (17·1, 18·7)	-18·5 (-22·3, -14·6)
**Portugal**	2678 (2590, 2759)	19·6 (19, 20·2)	4529 (4205, 4875)	17·8 (16·6, 19·2)	-9·2 (-16·3, -0·9)
**Spain**	9042 (8814, 9267)	16 (15·6, 16·4)	16955 (15815, 18236)	16·1 (15, 17·3)	0·7 (-6·8, 8·7)
**Sweden**	2756 (2689, 2825)	17·1 (16·7, 17·5)	3163 (2990, 3341)	14·3 (13·5, 15·1)	-16·2 (-21·2, -11)
**Switzerland**	1000 (972, 1031)	9·1 (8·8, 9·3)	1859 (1712, 2023)	10·2 (9·4, 11·2)	12·9 (2·6, 23·6)
**United Kingdom**	20755 (20510, 21014)	21·7 (21·5, 22)	20242 (19811, 20684)	15·1 (14·8, 15·5)	-30·3 (-32·1, -28·3)
**Southern Latin America**	7253 (7083, 7432)	15·8 (15·5, 16·2)	13402 (12431, 14491)	16·1 (14·9, 17·4)	1·5 (-6·4, 10·3)
**Argentina**	5349 (5185, 5517)	16·6 (16·1, 17·1)	9286 (8332, 10284)	17·1 (15·3, 19)	2·9 (-7·6, 14·8)
**Chile**	1033 (996, 1072)	10·7 (10·3, 11·1)	2898 (2620, 3203)	12·5 (11·3, 13·8)	16·3 (3·7, 30)
**Uruguay**	870 (842, 898)	21·6 (21, 22·3)	1218 (1092, 1353)	21·1 (18·9, 23·5)	-2·5 (-13·1, 8·7)
**Eastern Europe**	44989 (44037, 46443)	15·8 (15·5, 16·3)	57216 (55845, 58856)	16·4 (16, 16·9)	3·9 (1·6, 6·5)
**Belarus**	1928 (1876, 1985)	14·5 (14·2, 14·9)	2428 (2219, 2762)	15 (13·7, 17)	3 (-6·2, 16·5)
**Estonia**	358 (345, 371)	16·9 (16·3, 17·5)	439 (380, 498)	15·3 (13·3, 17·4)	-9·5 (-21·7, 3·9)
**Latvia**	618 (600, 637)	16·6 (16·1, 17·1)	714 (638, 801)	16·6 (14·8, 18·7)	0·1 (-11·7, 13)
**Lithuania**	708 (688, 727)	15·2 (14·7, 15·6)	984 (912, 1051)	16 (14·9, 17·2)	5·7 (-2, 13·9)
**Moldova**	701 (675, 726)	15·4 (14·9, 15·9)	898 (847, 956)	15·7 (14·7, 16·7)	1·6 (-5·1, 8·5)
**Russia**	28579 (27862, 29819)	15·6 (15·2, 16·3)	38591 (37903, 39269)	16·4 (16·1, 16·7)	5 (1·1, 8·4)

**Ukraine**	12097 (11725, 12485)	16·4 (15·9, 16·9)	13161 (12384, 14111)	16·8 (15·8, 18)	2·5 (-3·9, 9·4)
**Central Europe**	31145 (30745, 31541)	20·9 (20·7, 21·2)	45941 (44622, 47394)	20·9 (20·3, 21·6)	-0·1 (-3·1, 3·4)
**Albania**	138 (127, 169)	6·4 (5·8, 8·1)	293 (239, 359)	7·1 (5·8, 8·6)	11·2 (-9·8, 36·4)
**Bosnia and Herzegovina**	524 (490, 614)	13·4 (12·5, 15·8)	1215 (1114, 1328)	20·4 (18·8, 22·2)	52·4 (36·1, 67·5)
**Bulgaria**	2119 (2049, 2192)	16·7 (16·1, 17·2)	2914 (2709, 3113)	19·2 (17·9, 20·5)	15·5 (6·7, 23·8)
**Croatia**	1390 (1345, 1441)	22 (21·3, 22·7)	2181 (2037, 2339)	23·7 (22·2, 25·4)	8 (0·2, 16·3)
**Czech Republic**	4690 (4562, 4806)	33·4 (32·5, 34·2)	4411 (4135, 4713)	20·6 (19·3, 22)	-38·3 (-42·6, -33·4)
**Hungary**	4493 (4372, 4607)	29·9 (29·2, 30·7)	5194 (4864, 5526)	26·1 (24·5, 27·8)	-12·6 (-18·2, -6·8)
**Macedonia**	222 (207, 250)	12·1 (11·2, 13·9)	511 (454, 571)	15 (13·4, 16·8)	24·4 (3·4, 42·8)
**Montenegro**	84 (76, 92)	13·7 (12·4, 15)	146 (132, 162)	14·5 (13·2, 16·1)	6·1 (-6·5, 20·8)
**Poland**	9668 (9400, 9935)	21·5 (21, 22·1)	15486 (14506, 16534)	21·6 (20·2, 23·1)	0·5 (-6·3, 7·7)
**Romania**	3445 (3339, 3554)	12·2 (11·8, 12·5)	6651 (6248, 7088)	17·6 (16·5, 18·7)	44·1 (35·2, 54·3)
**Serbia**	2439 (2237, 2685)	21·9 (20·2, 24·1)	3863 (3582, 4156)	23·9 (22·1, 25·8)	9 (-1·8, 20)
**Slovakia**	1392 (1336, 1449)	22·9 (22, 23·8)	2222 (1987, 2404)	24·5 (21·9, 26·4)	6·7 (-6·2, 16·9)
**Slovenia**	541 (521, 564)	21·9 (21·1, 22·8)	852 (790, 922)	19 (17·6, 20·6)	-13·4 (-20·7, -5·5)
**Central Asia**	4124 (4053, 4203)	8·7 (8·5, 8·9)	6105 (5822, 6372)	9 (8·6, 9·4)	3·7 (-0·6, 7·9)
**Armenia**	303 (289, 319)	11·2 (10·6, 11·7)	529 (500, 557)	12·8 (12·1, 13·4)	14·3 (6·3, 22·5)
**Azerbaijan**	406 (383, 429)	7·9 (7·4, 8·3)	780 (668, 885)	9 (7·8, 10·2)	14·9 (-1·3, 32)
**Georgia**	507 (481, 535)	8·2 (7·7, 8·6)	634 (585, 679)	10·7 (9·9, 11·4)	31 (21·1, 41·5)
**Kazakhstan**	1531 (1481, 1583)	11·9 (11·5, 12·3)	1807 (1677, 1955)	11·3 (10·5, 12·2)	-5·2 (-11·7, 1·2)
**Kyrgyzstan**	299 (283, 318)	9·9 (9·3, 10·5)	269 (249, 300)	6·5 (6·1, 7·2)	-33·8 (-39·5, -26·1)
**Mongolia**	80 (72, 89)	7·7 (7, 8·7)	135 (117, 153)	6·8 (5·9, 7·7)	-11·7 (-27·4, 4·3)
**Tajikistan**	187 (177, 197)	6·4 (6·1, 6·8)	328 (296, 367)	6·8 (6·1, 7·5)	5·5 (-5·4, 17)
**Turkmenistan**	121 (115, 127)	6·2 (5·9, 6·6)	253 (232, 274)	7·5 (6·9, 8·2)	21·2 (9·1, 33·6)
**Uzbekistan**	691 (664, 716)	5·9 (5·6, 6·1)	1368 (1216, 1522)	7·4 (6·7, 8·3)	26·8 (13·5, 40·6)

**Central Latin America**	5441 (5358, 5544)	6·7 (6·6, 6·8)	18272 (17589, 18925)	8 (7·7, 8·3)	20·4 (15, 25·4)
**Colombia**	1421 (1375, 1473)	8·5 (8·2, 8·8)	4413 (3979, 4918)	8·2 (7·4, 9·1)	-3·7 (-13·6, 7·9)
**Costa Rica**	161 (154, 167)	9·4 (9, 9·8)	642 (581, 698)	13·2 (11·9, 14·3)	40·5 (25·5, 54·8)
**El Salvador**	148 (138, 160)	5 (4·7, 5·5)	495 (419, 578)	8·6 (7·3, 10·1)	71 (40·3, 100·9)
**Guatemala**	156 (149, 163)	4·7 (4·5, 4·9)	687 (619, 757)	6·6 (5·9, 7·3)	40·5 (24·5, 57·5)
**Honduras**	105 (92, 118)	5·1 (4·4, 5·7)	409 (313, 501)	7·1 (5·5, 8·7)	38·2 (7·4, 73·6)
**Mexico**	2448 (2400, 2505)	5·8 (5·7, 5·9)	8471 (8172, 8704)	7·6 (7·3, 7·8)	31·6 (25·3, 36)
**Nicaragua**	86 (79, 94)	5·5 (5·1, 6)	294 (260, 333)	6·6 (5·8, 7·5)	19·4 (2·5, 38·4)
**Panama**	127 (121, 133)	8·7 (8·3, 9·2)	369 (340, 399)	9·3 (8·5, 10)	6·4 (-3·2, 16·6)
**Venezuela**	788 (760, 817)	8·4 (8·1, 8·7)	2491 (2161, 2854)	9·3 (8, 10·7)	10·7 (-5·1, 28·2)
**Andean Latin America**	1425 (1293, 1628)	7·2 (6·6, 8·2)	4352 (3952, 4733)	8·2 (7·4, 8·9)	13·3 (-1·7, 28·7)
**Bolivia**	293 (183, 472)	9·8 (6·3, 15·6)	844 (620, 1122)	10·5 (7·8, 13·9)	7·2 (-23·1, 47·3)
**Ecuador**	354 (342, 368)	6·8 (6·6, 7·1)	1243 (1137, 1364)	8·7 (7·9, 9·5)	27 (14·4, 41·1)
**Peru**	777 (716, 842)	6·7 (6·2, 7·3)	2266 (1942, 2616)	7·4 (6·3, 8·5)	10 (-7·6, 30)
**Caribbean**	3153 (3040, 3335)	12·3 (11·9, 13)	6735 (6263, 7276)	13·3 (12·3, 14·3)	7·6 (1·3, 14·4)
**Antigua and Barbuda**	5 (5, 6)	10·2 (9·6, 11)	11 (10, 12)	11·6 (10·6, 12·6)	13 (1·5, 25·3)
**The Bahamas**	23 (21, 24)	14·9 (13·9, 15·9)	55 (50, 61)	15·7 (14·1, 17·4)	5·2 (-7·8, 20·3)
**Barbados**	49 (46, 52)	15·7 (14·8, 16·6)	89 (81, 97)	18·2 (16·5, 19·8)	16·3 (3·4, 28·8)
**Belize**	6 (5, 7)	6·5 (5·9, 7·1)	21 (19, 22)	8·3 (7·6, 9)	26·6 (11·6, 41·2)
**Bermuda**	14 (13, 14)	22 (20·5, 23·5)	21 (19, 23)	16·1 (14·7, 17·8)	-26·7 (-35, -16·3)
**Cuba**	1526 (1480, 1570)	14·8 (14·3, 15·2)	2953 (2643, 3294)	15·4 (13·8, 17·2)	4·4 (-6·2, 16·3)
**Dominica**	7 (7, 8)	9·4 (8·8, 10·1)	11 (10, 12)	11·7 (10·7, 12·7)	23·5 (9·3, 37·7)
**Dominican Republic**	240 (222, 260)	6·6 (6·1, 7·1)	834 (717, 956)	9·2 (7·9, 10·6)	39·7 (16·8, 63·1)
**Grenada**	9 (9, 10)	12·8 (12, 13·6)	21 (19, 23)	13 (11·9, 14)	1 (-8·8, 11·3)
**Guyana**	35 (33, 37)	9·4 (8·9, 9·9)	58 (51, 65)	10·2 (9, 11·4)	8·3 (-5·4, 23·8)

**Haiti**	309<br>(221, 478)	10·6<br>(7·9, 15·8)	700<br>(509, 1015)	11·9<br>(8·8, 16·8)	11·7<br>(-9·6, 41·5)
**Jamaica**	196<br>(184, 209)	10·6<br>(10, 11·3)	414<br>(352, 478)	14·2<br>(12, 16·4)	34·3<br>(11·5, 58·5)
**Puerto Rico**	452<br>(435, 469)	12·2<br>(11·7, 12·7)	930<br>(866, 995)	12·9<br>(12, 13·9)	6·2<br>(-1·6, 14·1)
**Saint Lucia**	9<br>(9, 10)	10·4<br>(9·7, 11)	20<br>(19, 22)	9·8<br>(9, 10·6)	-5·4<br>(-14·1, 4)
**Saint Vincent and the Grenadines**	8<br>(7, 8)	10·5<br>(9·8, 11·2)	16<br>(15, 17)	11·6<br>(10·6, 12·6)	10·5<br>(-1·4, 23·8)
**Suriname**	27<br>(25, 29)	11·2<br>(10·5, 12)	78<br>(70, 87)	14·1<br>(12·7, 15·6)	25·8<br>(11, 42·7)
**Trinidad and Tobago**	114<br>(109, 121)	13·9<br>(13·2, 14·6)	216<br>(180, 256)	12·3<br>(10·2, 14·6)	-11·2<br>(-26·4, 6·3)
**Virgin Islands**	16<br>(15, 18)	20·6<br>(18·7, 22·6)	42<br>(36, 48)	23·3<br>(20·2, 26·3)	13·3<br>(-5·1, 31·8)
**Tropical Latin America**	7428<br>(7255, 7637)	8·7<br>(8·5, 8·9)	23374<br>(22683, 24019)	10·3<br>(10, 10·6)	18·2<br>(12·9, 22·6)
**Brazil**	7293<br>(7122, 7501)	8·8<br>(8·5, 9)	22851<br>(22188, 23506)	10·3<br>(10, 10·6)	17·4<br>(12·2, 21·9)
**Paraguay**	135<br>(123, 147)	6·5<br>(5·9, 7·1)	522<br>(437, 619)	10·4<br>(8·7, 12·3)	59·5<br>(29·8, 92·8)
**East Asia**	80464<br>(75923, 88259)	9·4<br>(8·9, 10·3)	199982<br>(189925, 208686)	10·3<br>(9·8, 10·7)	9·5<br>(-3·7, 19·6)
**China**	75876<br>(71332, 83540)	9·3<br>(8·8, 10·3)	187078<br>(177047, 195514)	10·1<br>(9·6, 10·6)	8·2<br>(-5·6, 18·6)
**North Korea**	1394<br>(1129, 1687)	8·9<br>(7·3, 10·7)	2910<br>(2352, 3516)	9·5<br>(7·7, 11·4)	6·8<br>(-16·2, 37·8)
**Taiwan (Province of China)**	1852<br>(1809, 1898)	12·2<br>(11·9, 12·5)	6773<br>(6398, 7196)	17·7<br>(16·7, 18·8)	45·1<br>(36·3, 54·3)
**Southeast Asia**	22038<br>(19071, 26703)	9·3<br>(8·1, 11·1)	55681<br>(52786, 59309)	10·3<br>(9·8, 11)	11<br>(-8·1, 28·3)
**Cambodia**	506<br>(300, 893)	11·8<br>(7·2, 20·4)	1152<br>(869, 1530)	11·2<br>(8·6, 14·7)	-5<br>(-33, 40·1)
**Indonesia**	6487<br>(5443, 8189)	7·1<br>(6, 8·9)	14064<br>(13101, 15267)	7·6<br>(7·1, 8·3)	6·9<br>(-17·8, 29·6)
**Laos**	224<br>(140, 364)	11·4<br>(7·4, 18·2)	393<br>(308, 519)	10·2<br>(8·1, 13·4)	-10·5<br>(-34·2, 26)
**Malaysia**	1462<br>(1242, 1757)	18·2<br>(15·1, 21·8)	3707<br>(3249, 4191)	16·6<br>(14·6, 18·7)	-8·8<br>(-26·5, 8)
**Maldives**	6<br>(4, 9)	6·8<br>(4·8, 10·2)	14<br>(12, 16)	5·1<br>(4·4, 5·7)	-25·1<br>(-54·2, 10·8)
**Mauritius**	51<br>(48, 54)	7·4<br>(7, 7·8)	175<br>(161, 189)	11·1<br>(10·2, 12)	49·9<br>(36, 65·4)
**Myanmar**	2950<br>(1709, 4861)	13·4<br>(8, 21·8)	5185<br>(3912, 7136)	12·5<br>(9·5, 17·2)	-7<br>(-29·5, 35·9)
**Philippines**	1584<br>(1483, 1678)	5·6<br>(5·3, 5·9)	9007<br>(7875, 10206)	13·4<br>(11·8, 15·1)	139·8<br>(109·4, 176·2)
**Sri Lanka**	490<br>(456, 528)	4·9<br>(4·5, 5·3)	1312<br>(1029, 1567)	5·8<br>(4·5, 6·9)	18·4<br>(-7·6, 45·7)

**Seychelles**	7<br>(6, 8)	12<br>(10·9, 14·4)	22<br>(20, 24)	21·9<br>(19·7, 23·8)	82·9<br>(40·7, 107·9)
**Thailand**	3452<br>(3175, 3813)	10·1<br>(9·3, 11·1)	8445<br>(7641, 9362)	8·8<br>(8, 9·8)	-12·5<br>(-25·3, -0·2)
**East Timor**	17<br>(13, 27)	6·9<br>(5·3, 10)	63<br>(50, 81)	8·6<br>(7, 11)	25·6<br>(-9·5, 68·7)
**Vietnam**	4773<br>(4134, 5455)	12·2<br>(10·6, 13·8)	12068<br>(10561, 13786)	13·9<br>(12·3, 15·8)	14·6<br>(-7·3, 39·6)
**Oceania**	239<br>(198, 349)	8·9<br>(7·6, 12·4)	546<br>(457, 760)	9·3<br>(8·1, 12·3)	5·4<br>(-8·8, 20·1)
**American Samoa**	3<br>(2, 3)	13<br>(11·6, 14·5)	5<br>(4, 5)	12·7<br>(11·3, 14·1)	-2·8<br>(-17·5, 15·1)
**Federated States of Micronesia**	5<br>(4, 7)	11·1<br>(9·1, 14·2)	7<br>(5, 9)	11·6<br>(9·4, 14)	4·6<br>(-13·6, 26·1)
**Fiji**	26<br>(22, 31)	8<br>(6·9, 9·4)	59<br>(49, 68)	9·4<br>(7·8, 10·7)	17·2<br>(-7·5, 44·7)
**Guam**	9<br>(8, 10)	12·6<br>(11·3, 14·1)	23<br>(20, 25)	13·3<br>(12, 14·8)	5·1<br>(-10·7, 25·2)
**Kiribati**	3<br>(3, 3)	9<br>(8, 10·1)	6<br>(5, 7)	9·6<br>(7·9, 11·2)	6·1<br>(-15·7, 29·7)
**Marshall Islands**	2<br>(1, 3)	12·6<br>(9·6, 17·8)	4<br>(3, 5)	14·1<br>(11·5, 17·9)	11·8<br>(-6·6, 35·7)
**Northern Mariana Islands**	2<br>(1, 2)	11·5<br>(9·9, 13·7)	4<br>(4, 5)	9·6<br>(8·6, 10·8)	-16·6<br>(-31·5, 0·4)
**Papua New Guinea**	147<br>(111, 235)	8·3<br>(6·6, 12·9)	351<br>(270, 550)	8·6<br>(6·9, 13)	4·3<br>(-13·7, 25·6)
**Samoa**	7<br>(6, 9)	8·8<br>(7·2, 11)	11<br>(9, 13)	8·8<br>(7·3, 10·6)	0·4<br>(-20·7, 26·1)
**Solomon Islands**	9<br>(7, 14)	7·2<br>(5·6, 10·6)	23<br>(19, 30)	8<br>(6·6, 10·4)	11·6<br>(-9·3, 34·1)
**Tonga**	3<br>(3, 4)	7·2<br>(6·4, 8·3)	6<br>(5, 7)	7·6<br>(6·4, 8·8)	5·2<br>(-18, 29·9)
**Vanuatu**	7<br>(5, 9)	10·5<br>(8·2, 14·6)	18<br>(13, 24)	11·6<br>(9, 15·4)	10·9<br>(-15·1, 41·1)
**North Africa and Middle East**	12511<br>(10704, 16105)	7·6<br>(6·5, 9·7)	31342<br>(29922, 32855)	8<br>(7·6, 8·3)	4·2<br>(-19·1, 22·8)
**Afghanistan**	692<br>(298, 1542)	10·2<br>(4·7, 21·6)	1249<br>(713, 2330)	12<br>(7·4, 20·8)	18<br>(-11, 85·8)
**Algeria**	711<br>(630, 794)	6·1<br>(5·4, 6·8)	1915<br>(1683, 2122)	6·1<br>(5·4, 6·8)	0·9<br>(-17·2, 19·4)
**Bahrain**	17<br>(14, 19)	9·7<br>(8·3, 11·5)	57<br>(50, 64)	7<br>(6·3, 7·9)	-27·4<br>(-40·1, -8·1)
**Egypt**	1196<br>(1110, 1315)	4·4<br>(4·1, 4·9)	2880<br>(2354, 3313)	5·3<br>(4·3, 6·1)	20·8<br>(-5·6, 44·1)
**Iran**	1659<br>(1427, 2034)	6·9<br>(6, 8·4)	5592<br>(4979, 5856)	8·5<br>(7·6, 8·9)	22·6<br>(-3, 44·7)
**Iraq**	527<br>(427, 663)	7·1<br>(5·8, 8·8)	982<br>(893, 1067)	4·5<br>(4·1, 4·9)	-36·3<br>(-50·1, -19·4)
**Jordan**	139<br>(113, 167)	10<br>(8·1, 11·8)	535<br>(447, 621)	10·3<br>(8·6, 11·9)	3<br>(-22·8, 34·7)

**Kuwait**	34<br>(32, 37)	5·4<br>(5·1, 5·9)	131<br>(114, 155)	5·7<br>(4·9, 6·6)	4·6<br>(-8·5, 24·6)
**Lebanon**	286<br>(241, 335)	13·7<br>(11·6, 16·1)	741<br>(620, 869)	13·1<br>(11, 15·3)	-4·6<br>(-24·6, 17·5)
**Libya**	212<br>(172, 264)	11·4<br>(9·4, 14·1)	583<br>(496, 683)	13·2<br>(11·2, 15·4)	16·2<br>(-12·8, 52·9)
**Morocco**	783<br>(671, 921)	5·6<br>(4·9, 6·6)	2016<br>(1642, 2407)	6·7<br>(5·5, 8)	19·3<br>(-11·9, 55·7)
**Palestine**	116<br>(92, 146)	13·1<br>(10·4, 16·4)	296<br>(264, 325)	12·6<br>(11·2, 13·8)	-3·7<br>(-28·3, 27·9)
**Oman**	39<br>(31, 49)	6·1<br>(4·9, 7·7)	109<br>(90, 129)	6·5<br>(5·5, 7·6)	7<br>(-23·5, 41·3)
**Qatar**	12<br>(10, 15)	13·8<br>(11·6, 16·7)	63<br>(53, 75)	9·6<br>(8·1, 11·2)	-30·5<br>(-46·8, -9·5)
**Saudi Arabia**	337<br>(263, 440)	5·5<br>(4·3, 7·2)	1356<br>(1152, 1571)	9·3<br>(8, 10·5)	67·4<br>(19·1, 127·4)
**Sudan**	564<br>(373, 961)	6·3<br>(4·3, 10·3)	1209<br>(911, 1644)	7·1<br>(5·5, 9·6)	13·8<br>(-19·5, 63·4)
**Syria**	302<br>(251, 374)	5·8<br>(4·9, 7·2)	764<br>(633, 928)	6·4<br>(5·3, 7·8)	9·6<br>(-19·1, 40·8)
**Tunisia**	340<br>(300, 394)	7·4<br>(6·5, 8·6)	911<br>(719, 1126)	7·9<br>(6·2, 9·7)	6·1<br>(-23·5, 40)
**Turkey**	4181<br>(3346, 5391)	11·9<br>(9·6, 15·2)	8562<br>(7720, 9768)	9·9<br>(8·9, 11·3)	-16·6<br>(-38·2, 4·1)
**United Arab Emirates**	42<br>(30, 59)	11·4<br>(8, 15·9)	370<br>(296, 455)	13·1<br>(10·8, 15·6)	14·7<br>(-23·6, 67)
**Yemen**	313<br>(175, 547)	6·6<br>(4, 10·9)	992<br>(706, 1432)	8·3<br>(6·1, 11·6)	26·8<br>(-11·9, 101·5)
**South Asia**	32224<br>(28530, 38770)	5·9<br>(5·2, 7)	86712<br>(77540, 93391)	7·1<br>(6·4, 7·6)	20·4<br>(-6·2, 42·8)
**Bangladesh**	4476<br>(3701, 5683)	9·3<br>(7·8, 12)	8147<br>(6954, 9748)	7·1<br>(6·1, 8·6)	-23·3<br>(-42·2, -2·4)
**Bhutan**	15<br>(11, 23)	6·6<br>(4·9, 9·5)	40<br>(30, 51)	7·1<br>(5·5, 9)	8·3<br>(-27·2, 57·5)
**India**	23879<br>(20895, 29179)	5·5<br>(4·8, 6·7)	68596<br>(61647, 74084)	7<br>(6·2, 7·5)	26<br>(-2·4, 50·5)
**Nepal**	501<br>(346, 770)	5·7<br>(4, 8·6)	1267<br>(1020, 1631)	6·4<br>(5·2, 8·3)	13·1<br>(-16·8, 52·1)
**Pakistan**	3352<br>(2996, 3708)	6<br>(5·3, 6·6)	8662<br>(6511, 10650)	8·5<br>(6·4, 10·3)	41·1<br>(6·4, 74·2)
**Southern sub-Saharan Africa**	2226<br>(2046, 2413)	8·5<br>(7·7, 9·3)	4824<br>(4423, 5138)	9·4<br>(8·6, 10)	10·8<br>(3·3, 18·1)
**Botswana**	45<br>(37, 54)	8·6<br>(7·2, 10·1)	107<br>(90, 133)	9·1<br>(7·7, 11)	5·7<br>(-18·1, 31·2)
**Lesotho**	65<br>(53, 87)	7·2<br>(6, 9·4)	110<br>(86, 138)	10·2<br>(8, 12·8)	42·1<br>(10·6, 78·4)
**Namibia**	52<br>(43, 67)	7·6<br>(6·3, 9·7)	103<br>(88, 121)	7·8<br>(6·6, 9·1)	1·7<br>(-25·9, 34)
**South Africa**	1695<br>(1521, 1888)	8·4<br>(7·4, 9·4)	3757<br>(3338, 4056)	9·1<br>(8·1, 9·9)	9<br>(1·7, 16·8)

**Swaziland**	27<br>(22, 34)	10·3<br>(8·6, 12·9)	61<br>(48, 76)	12·2<br>(9·7, 15)	18·7<br>(-9·3, 51·5)
**Zimbabwe**	343<br>(302, 388)	9<br>(7·9, 10·1)	687<br>(587, 805)	11·1<br>(9·5, 12·9)	23·6<br>(3·9, 47·6)
**Western sub-Saharan Africa**	6615<br>(5380, 8667)	8·2<br>(6·7, 10·8)	13259<br>(11407, 15582)	8·4<br>(7·3, 9·8)	2·7<br>(-22·7, 31·1)
**Benin**	127<br>(102, 152)	6·6<br>(5·3, 7·9)	317<br>(255, 399)	7·6<br>(6·2, 9·5)	15·9<br>(-7·8, 47·6)
**Burkina Faso**	478<br>(385, 557)	12·4<br>(10·2, 14·3)	1009<br>(845, 1188)	13<br>(11, 15·2)	5·3<br>(-17, 35·6)
**Cameroon**	337<br>(284, 394)	8·4<br>(7·1, 9·7)	908<br>(667, 1154)	9·1<br>(6·8, 11·4)	8·7<br>(-15·4, 33·5)
**Cape Verde**	8<br>(7, 9)	3·5<br>(3·2, 3·9)	33<br>(30, 37)	7·4<br>(6·6, 8·1)	108·5<br>(80·7, 143·9)
**Chad**	177<br>(137, 244)	6·5<br>(5·1, 8·9)	392<br>(299, 520)	8·1<br>(6·3, 10·7)	24·8<br>(3·8, 51·3)
**Côte d'Ivoire**	198<br>(173, 224)	5·6<br>(5, 6·3)	494<br>(394, 622)	5·7<br>(4·6, 7)	0·6<br>(-22·7, 28·5)
**The Gambia**	19<br>(15, 22)	5·7<br>(4·8, 6·7)	54<br>(38, 71)	6·3<br>(4·5, 8·2)	9·9<br>(-28·2, 52·4)
**Ghana**	437<br>(360, 549)	7·8<br>(6·5, 9·6)	1166<br>(938, 1386)	8·7<br>(7, 10·2)	11·7<br>(-22·9, 47·7)
**Guinea**	193<br>(171, 215)	6<br>(5·4, 6·7)	376<br>(303, 474)	7·5<br>(6·1, 9·4)	24·6<br>(-1·6, 58·4)
**Guinea-Bissau**	42<br>(23, 67)	11·3<br>(6·5, 17·7)	64<br>(45, 87)	10·6<br>(7·8, 14·1)	-6·7<br>(-30·6, 33·6)
**Liberia**	83<br>(66, 109)	7·7<br>(6·1, 10·1)	148<br>(110, 206)	8·7<br>(6·6, 12·1)	13·2<br>(-10·3, 41·6)
**Mali**	277<br>(247, 315)	7·5<br>(6·7, 8·4)	547<br>(400, 743)	7·3<br>(5·4, 9·8)	-2·5<br>(-30·6, 35·7)
**Mauritania**	86<br>(64, 117)	8·8<br>(6·6, 12)	157<br>(120, 203)	8·7<br>(6·7, 11·2)	-1·4<br>(-24·2, 34·8)
**Niger**	171<br>(118, 249)	6·8<br>(4·8, 9·7)	405<br>(294, 587)	6·5<br>(4·8, 9·3)	-4·1<br>(-19·8, 14·3)
**Nigeria**	3473<br>(2400, 5192)	8·5<br>(6, 12·6)	6162<br>(4559, 8246)	8·6<br>(6·5, 11·3)	0·7<br>(-32·7, 53)
**São Tomé and Príncipe**	5<br>(5, 6)	8·3<br>(7·5, 9·4)	12<br>(9, 15)	12·8<br>(9·8, 16·5)	52·9<br>(15·6, 100·2)
**Senegal**	277<br>(218, 359)	9·2<br>(7·3, 12)	520<br>(402, 643)	8<br>(6·2, 9·8)	-12·7<br>(-41·1, 26)
**Sierra Leone**	153<br>(110, 212)	8·1<br>(5·9, 11·3)	277<br>(211, 366)	9<br>(6·9, 11·9)	10·8<br>(-11·9, 39·4)
**Togo**	73<br>(59, 89)	6·5<br>(5·2, 7·8)	218<br>(169, 277)	7·4<br>(5·8, 9·2)	13·6<br>(-7, 37·3)
**Eastern sub-Saharan Africa**	7182<br>(5763, 9202)	10·6<br>(8·7, 13·3)	14245<br>(13187, 15152)	10·3<br>(9·5, 11)	-2·9<br>(-22·7, 20·4)
**Burundi**	173<br>(132, 231)	8·8<br>(6·7, 11·5)	296<br>(234, 386)	8·3<br>(6·8, 10·6)	-5·1<br>(-23·6, 18·9)
**Comoros**	22<br>(17, 29)	11·7<br>(9·4, 15·3)	47<br>(38, 57)	11·4<br>(9·4, 13·9)	-2·2<br>(-24·3, 27·9)

**Djibouti**	18<br>(11, 27)	13·3<br>(8·9, 19·7)	66<br>(45, 93)	13·6<br>(9·7, 18·7)	2·2<br>(-28·5, 50·8)
**Eritrea**	114<br>(81, 165)	13·1<br>(9·7, 18·6)	279<br>(217, 354)	13·9<br>(11·1, 17·2)	5·8<br>(-22·9, 51)
**Ethiopia**	2392<br>(1332, 3581)	14·5<br>(8·8, 21)	3973<br>(3518, 4380)	11·4<br>(10·2, 12·6)	-20·9<br>(-46·2, 36·3)
**Kenya**	600<br>(495, 738)	7·7<br>(6·3, 9·6)	1618<br>(1434, 1857)	8·7<br>(7·7, 9·9)	12·4<br>(-2·3, 26·8)
**Madagascar**	486<br>(371, 666)	9·9<br>(7·6, 13·4)	898<br>(678, 1218)	9·6<br>(7·4, 12·8)	-3·3<br>(-20·2, 18·1)
**Malawi**	187<br>(128, 226)	5·1<br>(3·6, 6)	405<br>(328, 484)	5·9<br>(4·8, 7)	15·9<br>(-9·9, 66·4)
**Mozambique**	667<br>(571, 770)	12·6<br>(10·7, 14·5)	1332<br>(1101, 1607)	14·1<br>(11·7, 16·9)	12·1<br>(-15, 46·2)
**Rwanda**	225<br>(168, 301)	8·4<br>(6·3, 11·3)	392<br>(251, 527)	7·8<br>(5·1, 10·4)	-7·9<br>(-29·8, 19·4)
**Somalia**	261<br>(138, 440)	11·4<br>(6·7, 18·5)	707<br>(456, 1092)	12·8<br>(8·5, 19·7)	12·5<br>(-16·8, 66·3)
**South Sudan**	228<br>(140, 352)	10·5<br>(6·8, 15·7)	361<br>(254, 527)	10·8<br>(7·7, 15·7)	3·2<br>(-22·8, 45·1)
**Tanzania**	994<br>(779, 1235)	10·4<br>(8·5, 12·8)	2079<br>(1761, 2436)	9·7<br>(8·3, 11·3)	-6·3<br>(-28·6, 20·3)
**Uganda**	475<br>(404, 552)	7·7<br>(6·7, 8·9)	1122<br>(937, 1326)	9<br>(7·6, 10·6)	16·8<br>(-6·6, 45·7)
**Zambia**	337<br>(270, 412)	13·2<br>(10·9, 16)	662<br>(565, 760)	12<br>(10·3, 13·8)	-9·2<br>(-31·2, 18·4)
**Central sub-Saharan Africa**	1745<br>(1388, 2306)	8·7<br>(7·2, 11)	3885<br>(3296, 4744)	8·8<br>(7·7, 10·3)	1<br>(-15·4, 20)
**Angola**	340<br>(226, 505)	9·6<br>(6·9, 13·7)	897<br>(729, 1090)	9·7<br>(7·9, 11·6)	0·3<br>(-29·7, 43·1)
**Central African Republic**	102<br>(60, 165)	9·7<br>(6·3, 15·1)	185<br>(107, 307)	9·7<br>(6·2, 15·1)	-0·2<br>(-20·2, 23·4)
**Congo**	119<br>(90, 155)	12<br>(9·6, 14·9)	261<br>(207, 329)	12·1<br>(9·7, 14·6)	1<br>(-21·5, 31·3)
**DR Congo**	1105<br>(883, 1424)	8<br>(6·6, 9·9)	2386<br>(1884, 3075)	8·1<br>(6·6, 10)	0·9<br>(-18·3, 24·7)
**Equatorial Guinea**	18<br>(11, 28)	10<br>(6·7, 15)	42<br>(28, 60)	10·3<br>(6·9, 14·4)	3·4<br>(-44·8, 73·4)
**Gabon**	62<br>(48, 80)	11·6<br>(9·4, 14·9)	114<br>(86, 143)	12<br>(8·9, 14·7)	2·7<br>(-26·2, 32·3)

Appendix Table 5: DALYs of colorectal cancer in 1990 and 2017 for both sexes and percentage change of age-standardised rates by 195 countries and territories.

	1990		2017		Percentage change in age-standardised rates between 1990 and 2017
	Counts (95% UI)	Rate (95% UI)	Counts (95% UI)	Rate (95% UI)	
**Global**	11538601 (11121326, 12222899)	275·6 (265·9, 291)	18984324 (18498172, 19486073)	235·7 (229·7, 242)	-14·5 (-20·4, -10·3)
**High-income North America**	1509702 (1483445, 1540998)	432·4 (424·8, 441·3)	1799369 (1740468, 1861903)	315·1 (304, 326·7)	-27·1 (-30, -24·4)
**Canada**	133615 (130309, 137102)	406·7 (396·8, 416·9)	186571 (174073, 200093)	293·5 (273·8, 314·5)	-27·8 (-33·1, -22·3)
**Greenland**	280 (252, 310)	751·7 (673·4, 833·4)	363 (329, 397)	519·8 (472·8, 567·4)	-30·8 (-40·8, -19·9)
**USA**	1375774 (1351283, 1404620)	435·2 (427·4, 444)	1612404 (1556587, 1671285)	317·5 (306, 329·5)	-27 (-30, -24·3)
**Australasia**	117155 (114230, 120078)	495·3 (483, 507·5)	143424 (131344, 155933)	316 (288·5, 343·6)	-36·2 (-41·7, -30·4)
**Australia**	93200 (90619, 95907)	472·2 (459·3, 485·3)	115419 (103690, 127616)	302·9 (271·5, 336·1)	-35·9 (-42·3, -28·9)
**New Zealand**	23956 (23178, 24811)	611·8 (591·8, 633·2)	28005 (26066, 30096)	386·1 (359·4, 414·4)	-36·9 (-41·4, -32·3)
**High-income Asia-Pacific**	676238 (663050, 691114)	324·4 (318·1, 331·5)	1135282 (1085385, 1201298)	287·1 (274·3, 304·5)	-11·5 (-14·8, -7·4)
**Brunei**	518 (447, 614)	437·7 (373·2, 516·5)	1706 (1554, 1873)	484·6 (443·1, 533·2)	10·7 (-9, 31·8)
**Japan**	592074 (580303, 606043)	341·8 (335, 349·8)	910770 (871289, 959408)	303·9 (290·3, 321·2)	-11·1 (-14·3, -7·2)
**Singapore**	10456 (10109, 10835)	423 (409·2, 438·2)	18033 (16621, 19815)	258·2 (238·4, 283·5)	-39 (-43·8, -33)
**South Korea**	73190 (70728, 75795)	216·7 (209·6, 224·2)	204774 (186466, 223813)	242·1 (221·1, 264·6)	11·7 (0·8, 22·6)
**Western Europe**	2268290 (2231706, 2307130)	394 (387·8, 400·7)	2426173 (2321415, 2536376)	290 (277·3, 303·6)	-26·4 (-29·5, -23·2)
**Andorra**	215 (175, 266)	360 (293·3, 443·1)	380 (303, 457)	288·6 (228·7, 347)	-19·9 (-35·6, -1·7)
**Austria**	51252 (49764, 52753)	440·7 (428·4, 453·8)	40531 (37671, 43436)	241·2 (224, 258·8)	-45·3 (-49·3, -41·4)
**Belgium**	64056 (62178, 66160)	415·9 (404·2, 429·5)	58975 (54510, 63722)	275 (254·3, 297·1)	-33·9 (-38·9, -28·4)
**Cyprus**	2308 (2031, 2669)	269·8 (237·3, 311·9)	4229 (3786, 4729)	227·1 (203·4, 254)	-15·8 (-30·6, 2·7)
**Denmark**	27545 (26778, 28355)	350·9 (340·6, 361·4)	39461 (36453, 42435)	362·4 (334·9, 389·9)	3·3 (-4·8, 11·9)
**Finland**	19054 (18485, 19642)	267·1 (259·1, 275·5)	24125 (22426, 26034)	216·4 (200·6, 234·7)	-19 (-25·3, -11·8)
**France**	320578 (311879, 330065)	388·7 (378·4, 399·7)	331644 (305455, 359837)	264·8 (243·8, 288·9)	-31·9 (-37·4, -25·7)

**Germany**	599093 (584953, 614734)	472·4 (461·1, 484·3)	528763 (474164, 586387)	305·7 (273·8, 339·2)	-35·3 (-42, -27·8)
**Greece**	28952 (27804, 30156)	189·2 (181·7, 197)	50520 (46864, 54363)	230·9 (214·3, 249·6)	22·1 (12·7, 32·2)
**Iceland**	804 (761, 847)	282·1 (267·5, 297·6)	1146 (1065, 1231)	222·5 (206·5, 238·3)	-21·1 (-27·7, -14·1)
**Ireland**	19406 (18718, 20037)	474·9 (458·3, 490·8)	21572 (19581, 24126)	303·3 (275·1, 339·5)	-36·1 (-42·1, -29)
**Israel**	16591 (15923, 17444)	336·8 (323·7, 354·1)	28400 (26141, 30694)	261·6 (241·2, 282·8)	-22·3 (-28·3, -15·9)
**Italy**	314644 (305889, 323828)	357·2 (347·1, 367·2)	345224 (319024, 376298)	264·7 (244·4, 288·7)	-25·9 (-31·7, -19·6)
**Luxembourg**	2452 (2340, 2570)	442·9 (422·8, 464·4)	2812 (2490, 3286)	299·2 (264·6, 350·5)	-32·5 (-40·9, -21·1)
**Malta**	1324 (1256, 1399)	303·2 (287·9, 319·9)	2391 (2210, 2588)	278·7 (257·7, 300)	-8·1 (-15·9, -0·6)
**Netherlands**	83813 (81528, 86305)	419·7 (408·5, 432·1)	116910 (109358, 125154)	365·5 (341·8, 391·3)	-12·9 (-19, -6·7)
**Norway**	28697 (28125, 29266)	441·9 (432·8, 450·6)	31032 (29534, 32786)	346·9 (329·8, 367·1)	-21·5 (-25·3, -17·2)
**Portugal**	52811 (51124, 54430)	382·8 (370·9, 394·3)	77701 (71988, 84607)	359·2 (332·9, 392)	-6·2 (-13·7, 3·2)
**Spain**	176861 (172312, 181803)	323·2 (314·9, 331·9)	283313 (262905, 306025)	319·1 (295·6, 346·1)	-1·3 (-8·7, 7·1)
**Sweden**	49740 (48440, 51065)	344·8 (336·1, 353·9)	53413 (50290, 56738)	279·8 (261·9, 297·2)	-18·9 (-23·8, -13·5)
**Switzerland**	19513 (18855, 20152)	191·8 (185·2, 198·1)	32347 (29681, 35692)	202·2 (185·6, 224·1)	5·4 (-4·1, 17)
**United Kingdom**	386393 (379232, 393649)	434·1 (426·4, 442·2)	348772 (338077, 360091)	298·4 (289·1, 308·4)	-31·3 (-33·3, -29·1)
**Southern Latin America**	152213 (148748, 156040)	319·8 (312·4, 327·5)	262320 (241353, 284521)	326·5 (300·2, 354·1)	2·1 (-6·4, 11·7)
**Argentina**	112689 (109366, 116151)	339·1 (329·2, 349·4)	184956 (164823, 205907)	354·9 (315·5, 396)	4·7 (-6·5, 17·6)
**Chile**	22398 (21442, 23336)	213·5 (204·7, 222·4)	56066 (50271, 62631)	243·2 (218, 271·6)	13·9 (1·1, 28·4)
**Uruguay**	17120 (16535, 17711)	437·1 (422·5, 452·1)	21286 (19015, 23792)	417·3 (372·1, 467·7)	-4·5 (-15·2, 7·2)
**Eastern Europe**	1056513 (1030734, 1100691)	365·7 (356·7, 381·2)	1233374 (1198127, 1275464)	367·7 (356·8, 380·9)	0·5 (-1·9, 3·2)
**Belarus**	45245 (43867, 46556)	342·1 (331·7, 352)	50267 (45745, 58162)	321·5 (292·5, 371·3)	-6 (-14·6, 8·1)
**Estonia**	7797 (7514, 8103)	372·7 (359·3, 387·2)	7718 (6652, 8869)	304·9 (261·3, 354·2)	-18·2 (-30·1, -5·5)
**Latvia**	13513 (13081, 13939)	367·6 (356·1, 378·8)	12937 (11422, 14668)	338 (296·9, 383·3)	-8 (-19·8, 4·9)
**Lithuania**	15518 (15051, 16008)	335·1 (324·9, 345·5)	18191 (16871, 19533)	334·3 (309·8, 359)	-0·2 (-7·8, 7·3)

**Moldova**	18019 (17334, 18672)	380·8 (366·8, 393·9)	21264 (19985, 22783)	375 (352·4, 401·2)	-1·5 (-8·7, 5·7)
**Russia**	667525 (647818, 705118)	356·2 (345·6, 376·4)	825663 (808189, 843306)	361·5 (353·8, 369·4)	1·5 (-3·3, 5)
**Ukraine**	288897 (279499, 298050)	396·5 (383·9, 408·7)	297334 (278528, 320404)	402·5 (377·1, 434·5)	1·5 (-4·9, 9)
**Central Europe**	688352 (677404, 698606)	451·7 (444·6, 458·2)	890774 (862730, 919500)	432·6 (418·9, 446·3)	-4·2 (-7·3, -0·7)
**Albania**	3649 (3403, 4092)	152·6 (141·9, 177·3)	6339 (5156, 7704)	159 (130·2, 193·2)	4·2 (-16·3, 27·6)
**Bosnia and Herzegovina**	13412 (12547, 15141)	303 (283·7, 346·1)	25028 (22891, 27275)	428·9 (392·6, 467·5)	41·6 (26·6, 56·3)
**Bulgaria**	50273 (48632, 51886)	391·1 (378·4, 403·1)	59194 (54757, 63345)	430·6 (398·9, 461·7)	10·1 (1·1, 18·7)
**Croatia**	29608 (28597, 30722)	451·6 (436·9, 468)	39528 (37014, 42356)	468·3 (439, 501·5)	3·7 (-3·9, 11·7)
**Czech Republic**	96834 (93887, 99258)	695·6 (674·3, 712·9)	82259 (76705, 88302)	408·8 (381·5, 438·5)	-41·2 (-45·4, -36·1)
**Hungary**	92061 (89502, 94576)	614·7 (598·2, 631·1)	100042 (93728, 106830)	545·1 (510·9, 582·2)	-11·3 (-17·1, -5·1)
**Macedonia**	5484 (5118, 5914)	275·5 (257, 300·2)	11488 (10171, 12903)	343·8 (304·6, 385·7)	24·8 (4·9, 42·8)
**Montenegro**	1861 (1683, 2043)	287·3 (260·5, 313·6)	2930 (2635, 3266)	294·9 (265·6, 328·3)	2·6 (-9·2, 16·8)
**Poland**	209768 (203779, 215728)	460·6 (448·1, 473·4)	292727 (274072, 312103)	433·8 (405·8, 463)	-5·8 (-12·3, 1·1)
**Romania**	84786 (81948, 87547)	293·7 (284·4, 303·1)	135371 (127054, 144669)	390·4 (367·1, 416·5)	32·9 (24·2, 42·5)
**Serbia**	58145 (53166, 64485)	492·5 (451·9, 543·4)	75937 (70329, 82173)	492·9 (454·9, 534·5)	0·1 (-10·2, 10·4)
**Slovakia**	31145 (29793, 32567)	512·3 (490·4, 536)	44977 (40500, 48963)	500·7 (451·8, 544·4)	-2·3 (-12·8, 7·5)
**Slovenia**	11326 (10896, 11830)	450·7 (433·5, 470·3)	14953 (13769, 16223)	368·1 (338·5, 400·2)	-18·3 (-25·1, -10·7)
**Central Asia**	111728 (109584, 114217)	217 (213·1, 221·4)	149884 (142576, 158028)	193·5 (184·4, 203·1)	-10·8 (-14·8, -6·7)
**Armenia**	8215 (7802, 8654)	276·3 (262, 290·4)	11059 (10418, 11676)	268·3 (252·7, 282·9)	-2·9 (-9·7, 3·9)
**Azerbaijan**	11715 (11018, 12496)	205·2 (193·4, 218)	20976 (17806, 24185)	207·8 (177·1, 237·2)	1·3 (-13·4, 16)
**Georgia**	13155 (12465, 13931)	207·1 (196·3, 219·1)	13922 (12881, 14890)	249·1 (230·7, 265·8)	20·3 (10·3, 30·7)
**Kazakhstan**	39857 (38531, 41265)	287·6 (277·6, 297·9)	42840 (39681, 46707)	244·8 (227·7, 265·8)	-14·9 (-21·1, -8·6)
**Kyrgyzstan**	7946 (7492, 8438)	245·8 (231·9, 260·7)	6712 (6176, 7641)	144 (132·9, 161·8)	-41·4 (-47, -33·3)
**Mongolia**	2092 (1899, 2324)	180·9 (164·3, 201·5)	3832 (3295, 4320)	152·4 (131·4, 171·8)	-15·8 (-30·2, -1·5)

**Tajikistan**	5379 (5028, 5747)	165 (155·5, 175)	9145 (8109, 10268)	150·8 (134·2, 168·8)	-8·6 (-19, 2)
**Turkmenistan**	3565 (3390, 3746)	157·7 (149·6, 165·8)	6286 (5772, 6835)	156·4 (143·8, 169·6)	-0·8 (-10·4, 9·5)
**Uzbekistan**	19803 (19081, 20554)	150·9 (145·5, 156·4)	35113 (30981, 39430)	149·8 (133, 167·3)	-0·7 (-12, 11·2)
**Central Latin America**	132803 (130620, 135407)	141·3 (139, 144·2)	421623 (404702, 437765)	176·7 (169·5, 183·3)	25 (19·4, 30·3)
**Colombia**	34940 (33859, 36117)	178·1 (172·7, 184·3)	97313 (86783, 109256)	181·1 (161·6, 203·2)	1·7 (-9·3, 14)
**Costa Rica**	3637 (3482, 3785)	192·6 (184·3, 200·5)	13744 (12485, 14866)	277·2 (251·7, 300·2)	43·9 (29·3, 58·1)
**El Salvador**	3559 (3313, 3957)	110·1 (102·8, 121)	10839 (9040, 12875)	189·8 (158·1, 226)	72·4 (39·3, 106·4)
**Guatemala**	4041 (3868, 4226)	98·2 (93·9, 102·7)	16611 (14812, 18562)	141·5 (126·7, 157·8)	44·2 (27·6, 62·6)
**Honduras**	2776 (2434, 3116)	116·8 (102, 130·9)	9699 (7197, 12136)	153·6 (115, 191·3)	31·6 (0·8, 68·1)
**Mexico**	59298 (58139, 60752)	122 (119·7, 125·1)	199997 (192808, 206324)	168·6 (162·8, 174)	38·2 (31·9, 42·7)
**Nicaragua**	2221 (2025, 2444)	120·9 (110·3, 132·2)	6652 (5841, 7604)	138·5 (121·8, 158)	14·6 (-2·3, 34)
**Panama**	2883 (2748, 3027)	181·6 (173, 190·5)	7716 (7091, 8374)	194·8 (179, 211·5)	7·3 (-2·3, 18·1)
**Venezuela**	19448 (18705, 20228)	179·9 (172·8, 186·6)	59053 (50765, 68065)	203·4 (175·2, 234)	13·1 (-3·7, 31·2)
**Andean Latin America**	33634 (30166, 38869)	150·2 (135·2, 172·7)	91524 (82564, 100036)	167·3 (150·9, 183)	11·4 (-4·4, 28·3)
**Bolivia**	7287 (4464, 11967)	207·8 (128·5, 336·8)	18501 (13477, 24744)	209·5 (152·4, 279·8)	0·8 (-29·7, 41·7)
**Ecuador**	8090 (7793, 8397)	137·7 (132·5, 143·1)	26076 (23477, 28833)	173·4 (156·4, 191·6)	25·9 (12·7, 40·5)
**Peru**	18257 (16753, 19845)	140·1 (128·7, 152·3)	46947 (40061, 54795)	152·6 (130·5, 178)	8·9 (-9·4, 29·9)
**Caribbean**	68337 (65265, 73353)	251·9 (240·8, 270)	138301 (127898, 150530)	272·4 (251·8, 296·4)	8·1 (1·6, 15·2)
**Antigua and Barbuda**	111 (104, 120)	214·7 (200·4, 230·7)	234 (215, 254)	230·6 (211·1, 249·4)	7·4 (-3·3, 19·2)
**The Bahamas**	572 (528, 614)	334·2 (309·3, 358·3)	1343 (1198, 1489)	343·4 (307·9, 380·3)	2·8 (-10·8, 18·1)
**Barbados**	923 (870, 975)	323·6 (306, 341·5)	1708 (1539, 1866)	365·7 (329·2, 400·5)	13 (0·6, 25·9)
**Belize**	129 (117, 141)	131 (118·8, 143·9)	508 (468, 553)	180·9 (166·6, 196·7)	38 (21·9, 55·1)
**Bermuda**	284 (265, 302)	439·5 (409·6, 467·5)	366 (330, 405)	301·3 (271·1, 333·8)	-31·4 (-39·4, -21·1)
**Cuba**	31441 (30492, 32447)	298 (289, 307·4)	55309 (49299, 61774)	302·8 (270, 337·8)	1·6 (-9·1, 13·7)

**Dominica**	137 (127, 147)	193·9 (180·7, 207·7)	214 (196, 233)	239·2 (218, 260·9)	23·3 (9·7, 37·5)
**Dominican Republic**	5831 (5327, 6341)	138·6 (127·6, 150·4)	19119 (16315, 22004)	202 (172·5, 232·5)	45·8 (20·6, 71·8)
**Grenada**	189 (176, 201)	272·5 (254·3, 290·1)	398 (367, 431)	276·7 (255·1, 299·8)	1·6 (-8·8, 12·5)
**Guyana**	878 (829, 927)	205·8 (194·2, 217·3)	1478 (1297, 1677)	227 (199·9, 256·7)	10·3 (-4·1, 26·2)
**Haiti**	8207 (5722, 12937)	231·5 (164·6, 358)	17652 (12522, 26340)	246·4 (177, 359)	6·4 (-14·6, 38·8)
**Jamaica**	3748 (3511, 4021)	207·4 (194·5, 222·7)	8914 (7447, 10429)	309·1 (258·1, 360·5)	49·1 (21·8, 77)
**Puerto Rico**	9601 (9223, 9997)	258·8 (248·5, 269·5)	17840 (16594, 19213)	283 (263·4, 304·5)	9·3 (0·8, 17·7)
**Saint Lucia**	200 (188, 211)	217 (204·9, 229·5)	431 (397, 470)	205·7 (189·3, 223·3)	-5·2 (-14, 4·9)
**Saint Vincent and the Grenadines**	161 (151, 172)	215·2 (201·1, 229·4)	348 (318, 379)	257 (234·8, 279·7)	19·4 (6·5, 34·4)
**Suriname**	643 (595, 694)	236·1 (219·4, 254·2)	1769 (1574, 1967)	298·6 (266·9, 331·9)	26·5 (10·3, 44·3)
**Trinidad and Tobago**	2570 (2446, 2707)	287·3 (273·1, 302·8)	4817 (3966, 5792)	267·1 (219, 321·9)	-7 (-23·9, 11·8)
**Virgin Islands**	375 (338, 413)	416·3 (376·9, 456·6)	880 (751, 992)	481·3 (413·7, 542·4)	15·6 (-3·6, 35·6)
**Tropical Latin America**	183745 (179037, 189654)	183·3 (178·8, 188·9)	535234 (518062, 551007)	225·5 (218·3, 232)	23 (17·2, 27·8)
**Brazil**	180751 (176115, 186696)	184·6 (180·2, 190·4)	523766 (506827, 539532)	225·9 (218·6, 232·6)	22·3 (16·5, 27·1)
**Paraguay**	2994 (2714, 3292)	127·3 (115·8, 139·6)	11467 (9426, 13797)	210·2 (173, 252·1)	65 (32·5, 102·7)
**East Asia**	2247023 (2114932, 2455021)	221·3 (208·5, 241·6)	4539232 (4296171, 4749878)	219·5 (207·8, 229·5)	-0·8 (-12·1, 8·6)
**China**	2123210 (1993440, 2327462)	220·4 (206·8, 241·2)	4254095 (4013807, 4465821)	216·2 (203·9, 226·9)	-1·9 (-13·7, 7·8)
**North Korea**	38849 (31288, 47361)	214 (173·2, 259·1)	73129 (57895, 90993)	228·3 (181·6, 282·9)	6·7 (-18·5, 40·2)
**Taiwan (Province of China)**	47528 (46323, 48788)	272·6 (265·8, 279·5)	138884 (130578, 148287)	372·3 (350·6, 397)	36·6 (28·2, 45·5)
**Southeast Asia**	595032 (512296, 729874)	205·9 (178·1, 250·7)	1360792 (1286955, 1449817)	221 (209·3, 235·4)	7·3 (-13, 25·6)
**Cambodia**	13967 (8105, 25166)	272·3 (161·1, 483·7)	28983 (21523, 39397)	241·1 (180·6, 324·3)	-11·4 (-38·4, 33·3)
**Indonesia**	187414 (157128, 237584)	163 (137·1, 205·9)	350920 (326310, 376313)	157·4 (146·7, 169·5)	-3·5 (-26·8, 18·6)
**Laos**	6248 (3764, 10458)	266·5 (163·8, 438·2)	10360 (7818, 13951)	223·3 (172·1, 297·5)	-16·2 (-40·1, 22·7)
**Malaysia**	34163 (29733, 41524)	351·7 (302·5, 424·1)	83511 (73148, 95285)	322·1 (282·1, 366·2)	-8·4 (-26·5, 8·4)

**Maldives**	164 (98, 263)	156·2 (100·2, 243·4)	339 (296, 379)	105·3 (92·4, 117)	-32·6 (-60·2, 10·7)
**Mauritius**	1272 (1201, 1342)	159·8 (151·3, 168·6)	4013 (3679, 4381)	236·8 (217·3, 257·9)	48·2 (34·2, 63·7)
**Myanmar**	81606 (46324, 137028)	314·7 (180·5, 523·7)	127324 (94173, 176361)	270·4 (201·5, 372·8)	-14·1 (-37·1, 28·8)
**Philippines**	43963 (40988, 46550)	126 (117·6, 133·2)	245291 (212524, 278936)	309·1 (268·8, 350·8)	145·4 (111·1, 185·1)
**Sri Lanka**	12347 (11523, 13243)	104·6 (97·7, 112·4)	28796 (22506, 35090)	116·1 (91, 140·7)	10·9 (-13·4, 38·7)
**Seychelles**	159 (144, 193)	273·8 (247, 332·4)	524 (461, 575)	475 (419·7, 519·3)	73·5 (28·6, 99·9)
**Thailand**	94593 (86714, 105123)	228·3 (209·7, 252·8)	191793 (172618, 213989)	195·5 (176·1, 218·2)	-14·4 (-26·5, -2·8)
**East Timor**	525 (382, 819)	151·4 (112·7, 230)	1481 (1136, 1936)	178·6 (139·1, 232)	17·9 (-19·1, 63·7)
**Vietnam**	117821 (101574, 136533)	271·1 (234, 312·7)	285667 (242821, 333064)	299 (256, 346·5)	10·3 (-13·8, 38)
**Oceania**	7597 (6101, 11183)	212 (175·7, 306·6)	16944 (13657, 24444)	216·3 (180·8, 301·9)	2·1 (-12·8, 19·1)
**American Samoa**	71 (64, 79)	273·1 (243·1, 303)	121 (108, 136)	266·9 (238·9, 299·2)	-2·3 (-16·5, 16·5)
**Federated States of Micronesia**	132 (105, 178)	245·5 (196·9, 326·9)	189 (138, 246)	248·2 (190·5, 314·8)	1·1 (-20·2, 24·9)
**Fiji**	731 (617, 869)	172·5 (146·3, 203·7)	1530 (1259, 1770)	197·9 (163·9, 227·6)	14·7 (-11·1, 43·4)
**Guam**	244 (216, 278)	273 (244, 306·3)	556 (500, 622)	304·9 (275·7, 339·8)	11·7 (-6, 33·6)
**Kiribati**	89 (78, 99)	208·7 (183·8, 230·9)	165 (132, 200)	216 (173·4, 257·7)	3·5 (-19, 27)
**Marshall Islands**	56 (41, 79)	286·7 (211, 409·7)	118 (90, 158)	316·2 (248·9, 413·8)	10·3 (-8·8, 34·8)
**Northern Mariana Islands**	55 (46, 66)	235·6 (200·7, 283·6)	105 (92, 119)	195·6 (174·3, 220·1)	-17 (-32·9, 0·5)
**Papua New Guinea**	4969 (3671, 8022)	214·8 (164, 340·2)	11691 (8655, 18683)	212 (164, 330·2)	-1·3 (-19·3, 21·7)
**Samoa**	185 (150, 233)	202 (164·7, 253·6)	269 (217, 322)	190·4 (155·3, 228·4)	-5·8 (-27·3, 20·2)
**Solomon Islands**	286 (219, 438)	172·1 (133·8, 261·6)	663 (522, 892)	180·4 (147, 237·8)	4·8 (-15·6, 29·2)
**Tonga**	80 (70, 94)	139·8 (123·2, 162·9)	121 (102, 141)	149·1 (125·6, 172·9)	6·6 (-17·2, 32·2)
**Vanuatu**	197 (144, 281)	248 (187·8, 348·6)	483 (347, 671)	262·2 (192·6, 359·5)	5·7 (-21·9, 39·3)
**North Africa and Middle East**	336467 (283595, 442289)	174·5 (148·2, 226·7)	792334 (752239, 837918)	174·5 (166·2, 183·7)	0 (-22·8, 18·6)
**Afghanistan**	18694 (6997, 43686)	252·4 (96·2, 586·2)	38211 (20013, 75669)	290·1 (163·4, 539·9)	14·9 (-16, 112·7)

**Algeria**	17875<br>(15915, 19808)	130·8<br>(116·4, 144·7)	44968<br>(39104, 49938)	130·3<br>(113·3, 144·9)	-0·4<br>(-17·9, 17·4)
**Bahrain**	467<br>(414, 520)	206·5<br>(178·3, 239)	1673<br>(1462, 1902)	145·7<br>(129, 164·5)	-29·4<br>(-41·9, -11·3)
**Egypt**	37087<br>(33971, 40679)	108·4<br>(100·4, 118·8)	87183<br>(71754, 100712)	126·2<br>(103·3, 145·2)	16·5<br>(-8·2, 37·9)
**Iran**	46010<br>(39414, 56750)	153·7<br>(132·2, 188·4)	131737<br>(117790, 137842)	178·2<br>(159·1, 186·5)	15·9<br>(-9·5, 37·7)
**Iraq**	13759<br>(10809, 17890)	159·8<br>(127·2, 205·1)	26354<br>(23807, 28919)	99·6<br>(90·3, 109)	-37·7<br>(-52·9, -19)
**Jordan**	4106<br>(3306, 4956)	243<br>(197·2, 292·3)	14022<br>(11706, 16401)	213·3<br>(178·7, 247·4)	-12·2<br>(-35·3, 16)
**Kuwait**	1083<br>(1007, 1168)	120·6<br>(112·9, 129·8)	3558<br>(3056, 4270)	119·8<br>(102·6, 142·7)	-0·7<br>(-13·2, 19·3)
**Lebanon**	7186<br>(6043, 8460)	300·6<br>(253·4, 351·2)	17244<br>(14100, 20449)	276·6<br>(227·1, 328·3)	-8<br>(-29·1, 15·4)
**Libya**	5607<br>(4476, 7079)	263·2<br>(212·6, 329·9)	16136<br>(13649, 19062)	306·2<br>(260·5, 359·5)	16·3<br>(-13·3, 54·1)
**Morocco**	19352<br>(16566, 22989)	125·3<br>(107·2, 148·2)	47547<br>(38148, 57383)	143·9<br>(116·1, 173·1)	14·9<br>(-17·4, 51·1)
**Palestine**	3094<br>(2398, 4051)	317·1<br>(246·7, 410·5)	8073<br>(7192, 8862)	289·4<br>(257·8, 318·3)	-8·7<br>(-33·6, 23·7)
**Oman**	1053<br>(822, 1347)	129·7<br>(101·9, 164·7)	3040<br>(2451, 3652)	128·9<br>(105·9, 152·6)	-0·6<br>(-31, 35·1)
**Qatar**	380<br>(306, 479)	261·3<br>(217·5, 319·8)	1984<br>(1654, 2392)	181<br>(152, 215·2)	-30·7<br>(-47·9, -8·1)
**Saudi Arabia**	8842<br>(6813, 11661)	121<br>(93·5, 159)	41405<br>(34495, 49906)	196·8<br>(167·5, 225·5)	62·6<br>(13·4, 126·2)
**Sudan**	14819<br>(9265, 25907)	142·1<br>(92, 243·3)	30909<br>(22281, 44107)	154·1<br>(113·6, 213·3)	8·5<br>(-25·6, 61·3)
**Syria**	8348<br>(6803, 10540)	136·4<br>(112·4, 170·1)	19155<br>(15791, 23272)	139·2<br>(114·9, 168·5)	2<br>(-26·2, 34·7)
**Tunisia**	7658<br>(6767, 8842)	144·4<br>(127·4, 166·4)	19203<br>(15007, 23789)	155·6<br>(121·8, 192·8)	7·8<br>(-22·6, 42)
**Turkey**	110723<br>(86593, 144357)	281·6<br>(221·1, 365·7)	199670<br>(179919, 223887)	225·9<br>(203·6, 253·1)	-19·8<br>(-41, 1·8)
**United Arab Emirates**	1352<br>(956, 1873)	228·6<br>(160·7, 320·5)	12903<br>(10252, 16115)	261·8<br>(212·5, 316·7)	14·5<br>(-24·9, 68·7)
**Yemen**	8754<br>(4431, 16069)	151·6<br>(82·4, 270·8)	26618<br>(18215, 40273)	184·1<br>(129·1, 270·6)	21·4<br>(-19, 108·1)
**South Asia**	897358<br>(786161, 1090998)	134·4<br>(118·6, 162·2)	2129682<br>(1902148, 2300720)	150·6<br>(134·7, 162·5)	12·1<br>(-14·5, 34)
**Bangladesh**	132207<br>(105656, 171016)	230·3<br>(189·4, 292)	208296<br>(178567, 241444)	158·6<br>(135·5, 185·4)	-31·1<br>(-50·2, -9)
**Bhutan**	415<br>(294, 621)	146·2<br>(105·5, 216·9)	916<br>(690, 1184)	142·5<br>(108·3, 184)	-2·5<br>(-36·5, 44)
**India**	663174<br>(576191, 815109)	124·2<br>(108·4, 151·8)	1651581<br>(1491628, 1780292)	145·9<br>(131·5, 157·3)	17·4<br>(-10·9, 41·1)

**Nepal**	13812<br>(9319, 21595)	129·4<br>(89, 199·7)	29360<br>(23066, 37900)	132<br>(104·8, 170·1)	2<br>(-27, 41)
**Pakistan**	87751<br>(78200, 97102)	140·9<br>(126, 156·1)	239530<br>(177456, 298485)	188·6<br>(141·6, 233·2)	33·9<br>(-0·1, 67·5)
**Southern sub-Saharan Africa**	55250<br>(51814, 59245)	181·6<br>(169, 196·3)	115680<br>(106259, 123470)	198·4<br>(181·9, 211·9)	9·3<br>(-0·1, 16·9)
**Botswana**	1134<br>(916, 1381)	179<br>(147·8, 215·7)	2454<br>(2016, 3126)	172<br>(143·5, 216·1)	-3·9<br>(-27·3, 22·3)
**Lesotho**	1595<br>(1294, 2200)	153·4<br>(125·3, 208·5)	2782<br>(2158, 3543)	219·3<br>(171·2, 276·7)	43<br>(8, 84·3)
**Namibia**	1306<br>(1058, 1700)	168·4<br>(137·4, 217·3)	2317<br>(1955, 2757)	157·8<br>(133·3, 187)	-6·3<br>(-33·5, 25·3)
**South Africa**	41853<br>(38902, 45412)	180·6<br>(165·6, 199·1)	87711<br>(78349, 94952)	191<br>(170·1, 207·2)	5·8<br>(-2·7, 14·1)
**Swaziland**	711<br>(588, 904)	217·4<br>(180·5, 275·6)	1642<br>(1263, 2085)	264·3<br>(205·8, 333·3)	21·6<br>(-9·1, 57·9)
**Zimbabwe**	8651<br>(7589, 9839)	190·4<br>(168, 216·6)	18774<br>(15991, 22021)	246·6<br>(210·9, 288·4)	29·5<br>(6·9, 55·6)
**Western sub-Saharan Africa**	157130<br>(127879, 205389)	166·8<br>(135·6, 218·1)	321228<br>(273859, 381991)	167·8<br>(144, 197·6)	0·6<br>(-24·5, 29·6)
**Benin**	3001<br>(2382, 3602)	139·2<br>(111, 166·9)	7800<br>(6114, 9919)	156<br>(124·2, 197·8)	12<br>(-12·1, 44·2)
**Burkina Faso**	11933<br>(9512, 13948)	256·7<br>(206·6, 299)	25863<br>(21341, 30969)	271·3<br>(226·2, 321·6)	5·7<br>(-18, 38·5)
**Cameroon**	8724<br>(7344, 10149)	178·7<br>(150·4, 208·6)	23133<br>(16858, 29624)	187<br>(137·5, 238·4)	4·7<br>(-19·2, 30·8)
**Cape Verde**	185<br>(167, 203)	83·2<br>(75·2, 91·1)	653<br>(588, 719)	145·2<br>(130·7, 159·9)	74·5<br>(52·4, 103·2)
**Chad**	4161<br>(3211, 5768)	138·1<br>(106·2, 190·8)	9753<br>(7289, 13154)	167·8<br>(127·3, 224·9)	21·5<br>(-0·7, 50·1)
**Côte d'Ivoire**	5514<br>(4782, 6311)	119<br>(104·2, 134·6)	13139<br>(10310, 16575)	117·4<br>(93·1, 148·2)	-1·3<br>(-25, 28·4)
**The Gambia**	482<br>(395, 583)	122·7<br>(101·7, 146·3)	1277<br>(918, 1712)	130·2<br>(93·1, 174·2)	6·1<br>(-30·6, 49·8)
**Ghana**	11523<br>(9410, 14686)	163·2<br>(134·3, 205·9)	29682<br>(23860, 35715)	178·2<br>(143·6, 213·1)	9·2<br>(-25·3, 47·6)
**Guinea**	4530<br>(3993, 5033)	128·3<br>(113·4, 142·4)	9039<br>(7227, 11470)	159·4<br>(127·6, 202·6)	24·2<br>(-2·9, 61·5)
**Guinea-Bissau**	1132<br>(597, 1843)	252·3<br>(136·5, 407·4)	1769<br>(1214, 2459)	226·4<br>(159·8, 307·1)	-10·3<br>(-34·1, 32·5)
**Liberia**	1956<br>(1537, 2580)	163·9<br>(129·3, 215·7)	3727<br>(2731, 5210)	177·6<br>(130·5, 248·1)	8·4<br>(-15·8, 36·5)
**Mali**	7436<br>(6585, 8461)	167·5<br>(149, 190·2)	13958<br>(10037, 19215)	156<br>(113·3, 213·5)	-6·9<br>(-35·2, 32·8)
**Mauritania**	2050<br>(1511, 2801)	189·2<br>(140·3, 258·7)	3571<br>(2703, 4691)	172·7<br>(130·7, 226·5)	-8·7<br>(-30·9, 27·6)
**Niger**	4604<br>(3119, 6773)	144·4<br>(99·9, 210·5)	10505<br>(7589, 15427)	132·9<br>(96·7, 193·1)	-7·9<br>(-24·6, 12·1)

**Nigeria**	77487 (51958, 117393)	166·1 (113·6, 248·8)	142246 (102215, 196438)	164 (120·1, 222·6)	-1·2 (-34·9, 53·9)
**São Tomé and Príncipe**	120 (106, 138)	172 (152·1, 198·1)	279 (211, 367)	256·4 (195·1, 337·2)	49·1 (10·1, 96·9)
**Senegal**	6729 (5285, 8768)	192·6 (151·4, 250·4)	12253 (9495, 15341)	162·2 (125·5, 202·2)	-15·8 (-43·6, 23·8)
**Sierra Leone**	3603 (2582, 5029)	173·4 (124, 241·5)	6806 (5116, 9152)	184·7 (140·2, 246·9)	6·5 (-16·9, 36·7)
**Togo**	1956 (1557, 2368)	138·5 (111·1, 167·1)	5775 (4388, 7484)	152·7 (118·5, 194·9)	10·2 (-11·1, 36·3)
**Eastern sub-Saharan Africa**	195930 (153433, 255324)	232·2 (185·2, 298·4)	375287 (346163, 399939)	217·1 (200·8, 231·1)	-6·5 (-27·4, 20·1)
**Burundi**	4919 (3729, 6705)	199·8 (153·1, 267·2)	8269 (6465, 10975)	178·4 (141·1, 231·3)	-10·7 (-29·8, 14·3)
**Comoros**	611 (478, 809)	267·7 (210·9, 353·8)	1181 (956, 1441)	246 (199·3, 299·8)	-8·1 (-29·7, 21·4)
**Djibouti**	544 (341, 819)	295·6 (193·4, 441·7)	1843 (1245, 2637)	291·2 (201·1, 410·1)	-1·5 (-32·9, 51·6)
**Eritrea**	3601 (2431, 5245)	314·2 (224·5, 451·8)	8544 (6446, 10948)	316·2 (246·5, 400·6)	0·6 (-28·1, 49·5)
**Ethiopia**	67861 (36758, 103452)	309 (174, 461·2)	100689 (88689, 111252)	234·1 (206·3, 258·7)	-24·3 (-50·2, 39·9)
**Kenya**	15799 (13111, 19253)	169·9 (140·5, 208·2)	43133 (38172, 49581)	185 (163·7, 213)	8·8 (-4·7, 23)
**Madagascar**	13680 (10433, 19043)	231·3 (177·1, 318·3)	26062 (19447, 35515)	215·2 (162·4, 291·6)	-6·9 (-24·6, 16·2)
**Malawi**	4579 (2784, 5701)	104·2 (67·8, 127·4)	9570 (7653, 11485)	121·6 (97·9, 145·6)	16·7 (-12·4, 85)
**Mozambique**	17897 (15327, 20744)	263·3 (225·3, 304·3)	36928 (30176, 45204)	298·7 (246·5, 361)	13·4 (-14·6, 47·5)
**Rwanda**	6139 (4596, 8306)	188·3 (140·7, 252·6)	10224 (6459, 13847)	163·5 (104·5, 220·1)	-13·2 (-35·7, 16·4)
**Somalia**	7605 (3671, 12922)	254·2 (135·9, 428·1)	20065 (12684, 31777)	283·5 (183·5, 437·9)	11·5 (-22·3, 85)
**South Sudan**	6020 (3389, 9530)	228·6 (137·1, 360·1)	10092 (6981, 14996)	238·4 (167·7, 349·4)	4·3 (-26·3, 60·4)
**Tanzania**	25090 (18266, 31858)	211·8 (162·6, 265·4)	50756 (42833, 59993)	197·5 (166·3, 233·8)	-6·8 (-31·3, 25·8)
**Uganda**	12513 (10615, 14665)	171·5 (145·2, 200·3)	30274 (24885, 36046)	198·9 (164·8, 235·2)	16 (-8·7, 47)
**Zambia**	8972 (6941, 11247)	277·8 (222·3, 341·4)	17421 (14763, 20322)	246·5 (208·9, 284·4)	-11·3 (-34·6, 18·9)
**Central sub-Saharan Africa**	48102 (37027, 65441)	189·9 (151·2, 249·7)	105862 (87267, 133328)	189·5 (159·9, 231·8)	-0·2 (-16·7, 21·3)
**Angola**	9808 (6306, 15105)	216·6 (145·3, 321·5)	25203 (20599, 31234)	210·6 (172, 256·2)	-2·8 (-33·5, 44·7)
**Central African Republic**	2983 (1665, 4929)	225·8 (133·6, 361·4)	5500 (2984, 9448)	226·1 (132·6, 374·4)	0·2 (-21·5, 27·6)

**Congo**	3253<br>(2320, 4413)	272<br>(201·6, 359)	7060<br>(5381, 9293)	259·6<br>(204·4, 328·8)	-4·6<br>(-26·9, 29·9)
**DR Congo**	30036<br>(23309, 39900)	171·9<br>(137·1, 221·6)	64115<br>(49380, 85616)	172·8<br>(136, 223·7)	0·5<br>(-19·8, 25·6)
**Equatorial Guinea**	494<br>(282, 809)	227·9<br>(138·1, 363·3)	1107<br>(708, 1610)	215·8<br>(140·6, 307)	-5·3<br>(-50·7, 71·1)
**Gabon**	1528<br>(1138, 2054)	255·4<br>(193·4, 338·4)	2877<br>(2167, 3691)	258·8<br>(195·7, 325·7)	1·3<br>(-27·9, 34·3)

References

1. Roth GA, Abate D, Abate KH, et al. Global, regional, and national age-sex-specific mortality for 282 causes of death in 195 countries and territories, 1980–2017: a systematic analysis for the Global Burden of Disease Study 2017. *Lancet* 2018; **392**: 1736-88.
2. Global, regional, and national comparative risk assessment of 84 behavioural, environmental and occupational, and metabolic risks or clusters of risks for 195 countries and territories, 1990-2017: a systematic analysis for the Global Burden of Disease Study 2017. *Lancet* 2018; **392**: 1923-94.
